# Co-occurrence between mental disorders and physical diseases: a study of nationwide primary-care medical records

**DOI:** 10.1017/S0033291724002575

**Published:** 2024-11

**Authors:** Matthew R. Hanna, Avshalom Caspi, Renate M. Houts, Terrie E. Moffitt, Fartein Ask Torvik

**Affiliations:** 1 Department of Psychology & Neuroscience, Duke University, Durham, NC, USA; 2 Department of Psychiatry & Behavioral Sciences, Duke University School of Medicine, Durham, NC, USA; 3 Institute of Psychiatry, Psychology, & Neuroscience, King's College London, London, UK; 4 Promenta Research Center, University of Oslo, Oslo, Norway; 5 Centre for Fertility and Health, Norwegian Institute of Public Health, Oslo, Norway

**Keywords:** comorbidity, health registry data, integrated healthcare, mental disorders, mental physical comorbidity, nationwide cohort, physical-health conditions, primary care

## Abstract

**Background:**

Mental disorders and physical-health conditions frequently co-occur, impacting treatment outcomes. While most prior research has focused on single pairs of mental disorders and physical-health conditions, this study explores broader associations between multiple mental disorders and physical-health conditions.

**Methods:**

Using the Norwegian primary-care register, this population-based cohort study encompassed all 2 203 553 patients born in Norway from January 1945 through December 1984, who were full-time residents from January 2006 until December 2019 (14 years; 363 million person-months). Associations between seven mental disorders (sleep disturbance, anxiety, depression, acute stress reaction, substance-use disorders, phobia/compulsive disorder, psychosis) and 16 physical-health conditions were examined, diagnosed according to the International Classification of Primary Care.

**Results:**

Of 112 mental-disorder/physical-health condition pairs, 96% of associations yielded positive and significant ORs, averaging 1.41 and ranging from 1.05 (99.99% CI 1.00–1.09) to 2.38 (99.99% CI 2.30–2.46). Across 14 years, every mental disorder was associated with multiple different physical-health conditions. Across 363 million person-months, having any mental disorder was associated with increased subsequent risk of all physical-health conditions (HRs:1.40 [99.99% CI 1.35–1.45] to 2.85 [99.99% CI 2.81–2.89]) and vice versa (HRs:1.56 [99.99% CI 1.54–1.59] to 3.56 [99.99% CI 3.54–3.58]). Associations were observed in both sexes, across age groups, and among patients with and without university education.

**Conclusions:**

The breadth of associations between virtually every mental disorder and physical-health condition among patients treated in primary care underscores a need for integrated mental and physical healthcare policy and practice. This remarkable breadth also calls for research into etiological factors and underlying mechanisms that can explain it.

## Introduction

Mental disorders and physical diseases often co-occur in the same patients (Cunningham, Sarfati, Peterson, Stanley, & Collings, 2014; Grigoletti et al., 2009; Kisely, Smith, Lawrence, & Maaten, 2005; Lawrence, Hancock, & Kisely, 2013; Lawrence, Kisely, & Pais, 2010; Mitchell et al., 2013; Moussavi et al., 2007; Scott et al., 2016; Wahlbeck, Westman, Nordentoft, Gissler, & Laursen, 2011). This co-occurrence has prompted investigations into how mental disorders may contribute to poor physical health, and vice versa (Katon, 2003; Luppino et al., 2010; Moffitt & Caspi, 2019). Co-occurrence also has treatment implications, as mental disorders can interfere with help-seeking and treatment-adherence for physical diseases (DiMatteo, Lepper, & Croghan, 2000; Prince et al., 2007).

Despite progress in describing and explaining links between specific mental disorders and specific physical diseases, knowledge about the breadth and strength of associations between mental disorders and physical-health conditions in the population has been limited by conceptual and methodological challenges (Kessler et al., 2011; Prince et al., 2007). A conceptual challenge is that much research has focused on specific dyads of one mental disorder and one physical disease, such as depression and cardiovascular disease (Celano & Huffman, 2011; Dhar & Barton, 2016; Hare, Toukhsati, Johansson, & Jaarsma, 2014; Lett et al., 2004; Nemeroff & Goldschmidt-Clermont, 2012; Nicholson, Kuper, & Hemingway, 2006; Penninx, 2017) or schizophrenia and metabolic disorders (Henderson, Vincenzi, Andrea, Ulloa, & Copeland, 2015; Leonard, Schwarz, & Myint, 2012; Mitchell et al., 2013; Orešič et al., 2011; Papanastasiou, 2013; Ventriglio, Gentile, Stella, & Bellomo, 2015). While this dyad-focused approach has contributed valuable insights into associations between specific mental disorders and physical-health conditions, it risks perpetuating the ‘lamppost effect’, where researchers only detect associations they seek (Smith, 2011). Studying dyads masks broader patterns of mental–physical comorbidity. For instance, although the focus on the depression–cardiovascular disease dyad generated the inflammation hypothesis (Felger & Lotrich, 2013; Miller, Maletic, & Raison, 2009; Raison, Capuron, & Miller, 2006), this specific focus might not adequately reveal potential associations between depression and other physical-health conditions, or between cardiovascular disease and other mental disorders (Chen et al., 2023). Broadening knowledge of these associations is critical; doing so could reveal shared etiological factors and underlying mechanisms, informing more effective treatments for both mental disorders and physical-health conditions (Halstead et al., 2024). Studies that go beyond dyadic relationships are needed to offer a more comprehensive understanding of mental–physical comorbidity.

A methodological challenge arises from the data sources used to study mental–physical comorbidity. Valuable large-scale epidemiological surveys, such as the World Mental Health (WMH) survey (Scott et al., 2016), and nationwide hospital registry data (Chen et al., 2023; Momen et al., 2020; Richmond-Rakerd, D'Souza, Milne, Caspi, & Moffitt, 2021) have reported associations between multiple mental disorders and multiple physical-health conditions. However, epidemiological surveys are subject to recall bias, leading to underestimation of the co-occurrence of mental disorders and physical-health conditions (Takayanagi et al., 2014). Nationwide hospital registry data have been used to circumvent retrospective-recall bias (Chen et al., 2023; Momen et al., 2020; Richmond-Rakerd et al., 2021). Hospital registries are valuable for identifying severe cases requiring specialized care. However, hospital-register studies may overestimate associations between mental disorders and physical-health conditions in the population because people who receive hospital treatment typically have more severe, complex cases with multiple comorbid conditions (Cohen & Cohen, 1984).

To address these conceptual and methodological challenges, this study leveraged a nationwide dataset encompassing all primary-care medical encounters in Norway for over 2.2 million patients aged 20–60 years at baseline and followed from 2006 to 2019. In most healthcare systems, primary-care providers (PCPs) act as gatekeepers, responsible for initial diagnoses and referrals (Forrest, 2003; Rotar, Van Den Berg, Schäfer, Kringos, & Klazinga, 2018). They not only treat common ailments but also serve as a frontline defense against more complex health challenges (Starfield, Shi, & Macinko, 2005). Highlighting this essential role, a primary-care registry study in the UK provided valuable insights into the co-occurrence of physical-health conditions and the most severe mental disorders, including schizophrenia and bipolar disorder (Launders, Kirsh, Osborn, & Hayes, 2022). Our study builds on this line of research. By including all primary-care encounters for the entire Norwegian population, we provide a broader view of a wider range of mental symptoms and diagnoses, including sleep disturbance, anxiety, depression, acute stress reaction, substance-use disorders, phobia/compulsive disorder, and psychosis, and their associations with 16 physical-health conditions.

We pursued two lines of inquiry. First, we investigated the co-occurrence of multiple mental disorders and physical-health conditions over a 14-year observation period in a national primary-care register. Our goal was to move beyond a focus on individual mental-disorder/physical-health condition dyads, avoid retrospective recall failure that biases epidemiological surveys, and circumvent selection biases that typify hospital registers. Second, we tested for temporal associations to uncover the extent to which a mental disorder may signal that a patient will present with a physical-health condition in the future, and vice versa. We compared associations by sex, age, and university attendance. We generated an atlas of associations between mental disorders and physical-health conditions to highlight opportunities for future research into common etiologies and mechanisms, and the need for integrated healthcare practices.

## Methods

### Study population

This population-based cohort analysis incorporates 2 203 553 patients (1 121 267 males, 1 082 286 females) aged 20–60 years (*M* [s.d.] = 40.6 [11.3] years) at baseline, born in Norway from January 1945 through December 1984, who were full-time residents in Norway from January 2006 until December 2019 or until they died, as identified in the Norwegian Population Register.

### Identification and classification of mental disorders and physical-health conditions

Each resident of Norway is assigned a PCP, and specialist healthcare typically necessitates a referral from the PCP. For reimbursement purposes, PCPs submit at least one diagnosis or reason for the visit to the Norwegian Health Economics Administration. Thus, it is unlikely that visits to PCPs go unreported. Medical encounters are coded using the International Classification of Primary Care, 2nd edition (ICPC-2) (Verbeke, Schrans, Deroose, & De Maeseneer, 2006).

For mental disorders, we focused on those with a population prevalence of 2.5% or higher to ensure robust statistical analysis: sleep disturbance (18.9%), anxiety (13.4%), depression (25.6%), acute stress reaction (21.2%), substance-use disorders (6.8%), phobia/compulsive disorder (2.7%), and psychosis (2.7%). These conditions were identified using codes from ICPC-2 chapter P (Psychological). Although prevalent, we excluded the ‘Other’ diagnosis in ICPC-2 (18.2%) because this designation precludes identifying a disorder type. For physical-health conditions, we focused on nine disease categories (gastrointestinal, neurological, pulmonary, hematological, urogenital, endocrine, circulatory, musculoskeletal, and cancers) previously reported in a nationwide hospital registry study of mental disorders and physical-health conditions (Momen et al., 2020, 2024). Because previous hospital registry research used ICD-10 codes, we used a Norwegian Directorate of eHealth mapping system (ICPC-2e – English version: The Directorate of e-health, n.d.) to convert these ICD-10 codes into their corresponding ICPC-2 codes. In addition to these nine disease categories, we also report seven physical-health conditions: infections, pain, headaches, fractures, skin, ear and eye conditions, which were defined directly from their respective ICPC-2 codes. In total, our study encompassed 16 total physical-health conditions. Additional details, including specific diagnostic codes and prevalence rates, are available in online Supplementary Tables S1–S3. Over the 14-year observation period between 2006 and 2019, 97.5% of the population studied had an ICPC-2 code for at least one of the conditions studied here. These patients averaged 49.5 (s.d. = 70.6) primary-care visits in the 14 years between 2006 and 2019. Of these clinician–patient consultations, 15% involved telephone communications, and 0.3% involved home consultations. The average time between visits was *M* = 80.1 days (s.d. = 211.7) and the average time between first and last visit was *M* = 3881.5 days (s.d. = 1336.5). Most visits (88.7%) yielded one code; 10.3% two codes; and 1.0% three or more codes (*M* = 1.13, s.d. = 0.39). On average, patients accumulated *M* = 55.8 (s.d. = 84.0) codes between 2006 and 2019.

### Statistical analysis

We report associations (odds ratios and 99.99% confidence intervals) between mental disorders and physical-health conditions over the 14-year observation window, beginning with the presence of ‘any’ mental disorder, as indicated by a binary indicator, and then reporting each specific mental disorder. Inverse probability weighting was used to balance differences between patients who presented with a mental disorder *v.* patients without a mental disorder on four demographic variables: sex assigned at birth, and age, educational attainment, and county of residence at baseline (January 2006).

We also compared associations between mental disorders and physical-health conditions separately for men *v.* women; younger (aged 20–40 years old at baseline) *v.* older (aged 40–60 years old at baseline) adults; and among adults with *v.* without a university education. In each of these three stratified analyses, we used inverse probability weighting to balance groups on the remaining three demographic variables.

We used extended Cox proportional-hazards models to test associations between each of the 7 (+any) mental disorders and 16 physical-health conditions across the 363-million-person months covered during the 14-year observation period, 2006–2019. In each month, we determined whether or not patients had an encounter with their PCP for a mental disorder or physical-health condition. Models testing whether mental-disorder diagnoses were associated with increased risk of subsequent physical-health conditions, and models testing whether physical-health conditions were associated with increased risk of subsequent mental-disorder diagnoses, were fit separately. In both cases, independent exposures were treated as time-varying covariates and the dependent time-to-event variables were considered recurrent events. Models were estimated using the (start, stop) or counting process approach; recurrent events were treated as identical (e.g. subsequent events were not assumed to be more or less severe than the first observed event) and time-dependent exposures were parameterized to represent the overall effect on survival time (Kleinbaum & Klein, 1996). Inverse probability weights were used to account for effects due to sex, and age, educational attainment, and county of residence at baseline (January 2006). Because dependent time-to-event variables were considered recurrent events, the same individuals could contribute multiple events to the analysis. While we acknowledge that different intervals contributed by a given individual likely represent correlated observations on the same individual that must be accounted for in the analysis, we did not use a robust estimator due to computing constraints (i.e. each model took >36 h to run and running multiple such models was prohibitive). Instead, we randomly selected six models to run using robust estimation and observed that the standard errors increased an average of 1.67-fold (range: 1.30–2.02) in these models relative to models run without robust estimators. Nonetheless, given that we estimated our models on a population of over two million individuals, our standard errors were exceedingly small (generally <0.005), such that multiplying standard errors by two or even three did not result in meaningful substantive changes to our findings.

Analyses were performed in SAS v9.4 TS Level 1M4; graphs were created in R using plotly (v4.10.4). Reported results were checked for reproducibility by an independent data analyst, who recreated the code by working from the manuscript and applying it to a fresh copy of the dataset.

## Results

**Figure 1.**
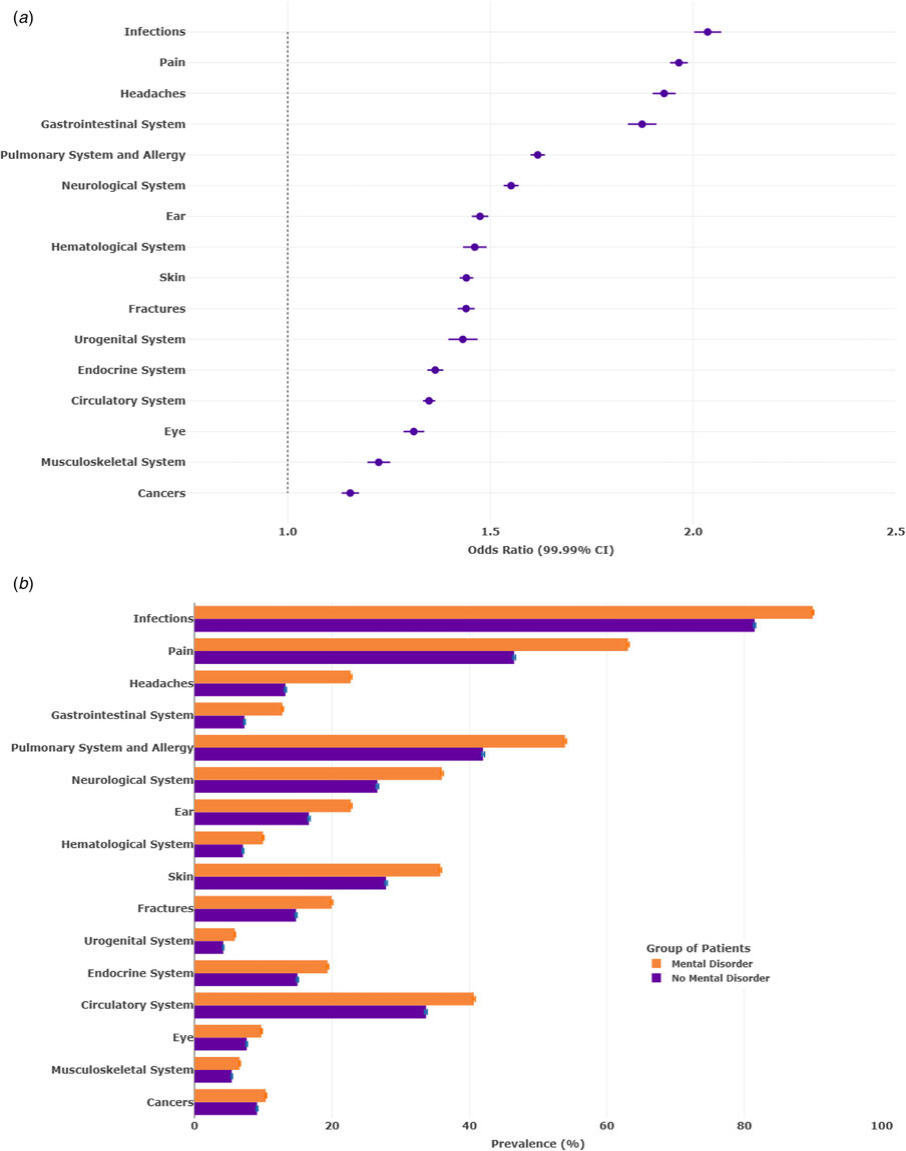
Associations between any mental disorder and 16 physical-health conditions in primary-care practices across 14 years. Panel A shows associations (odds ratios and 99.99% confidence intervals) between any mental-disorder diagnosis (a binary variable) and 16 physical-health conditions. The figure is organized from the weakest (left and bottom) to the strongest (right and top) associations. Panel B shows the risk of 16 physical-health conditions among patients who did and did not present with any mental disorder. Inverse probability weighting was used to balance on four demographic variables: age at baseline, sex assigned at birth, educational attainment, and county of residence. Results for specific mental disorders are shown in online Supplementary Figs S1–S7. (A) Associations between any mental disorder and 16 physical-health conditions. (B) Risk of 16 physical-health conditions for patients with and without any mental disorder.

Of the 2 203 553 patients in the cohort, 41.6% of males and 58.7% of females presented to primary care for mental disorders under consideration over the 14-year observation window, between 2006 and 2019. Patients who presented to primary-care practitioners with a mental disorder were significantly more likely to experience all of the 16 physical-health conditions assessed (Fig. 1a and b). The forest plot in Fig. 1a shows that patients presenting with any mental disorder, as indicated by a binary variable, were most likely to experience infections (OR = 2.04, 99.99% CI 2.00–2.07), pain (OR = 1.96, 99.99% CI 1.94–1.99), headaches (OR = 1.93, 99.99% CI 1.90–1.96), and gastrointestinal conditions (OR = 1.87, 99.99% CI 1.84–1.91). They were least likely to experience eye conditions (OR = 1.31, 99.99% CI 1.29–1.34), musculoskeletal conditions (OR = 1.22, 99.99% CI 1.20–1.25), and cancer (OR = 1.15, 99.99% CI 1.13–1.18), although these conditions were also significantly elevated among patients with a mental disorder. To delineate associations likely to be of public health significance, we note prevalent physical-health conditions affecting more than 20% of the weighted population and in which the risk difference between patients presenting with a mental disorder *v.* those without a mental disorder exceeded 10% (Fig. 1b): pain (63% *v.* 47%), pulmonary conditions (54% *v.* 42%), and headaches (23% *v.* 13%).

**Figure 2.**
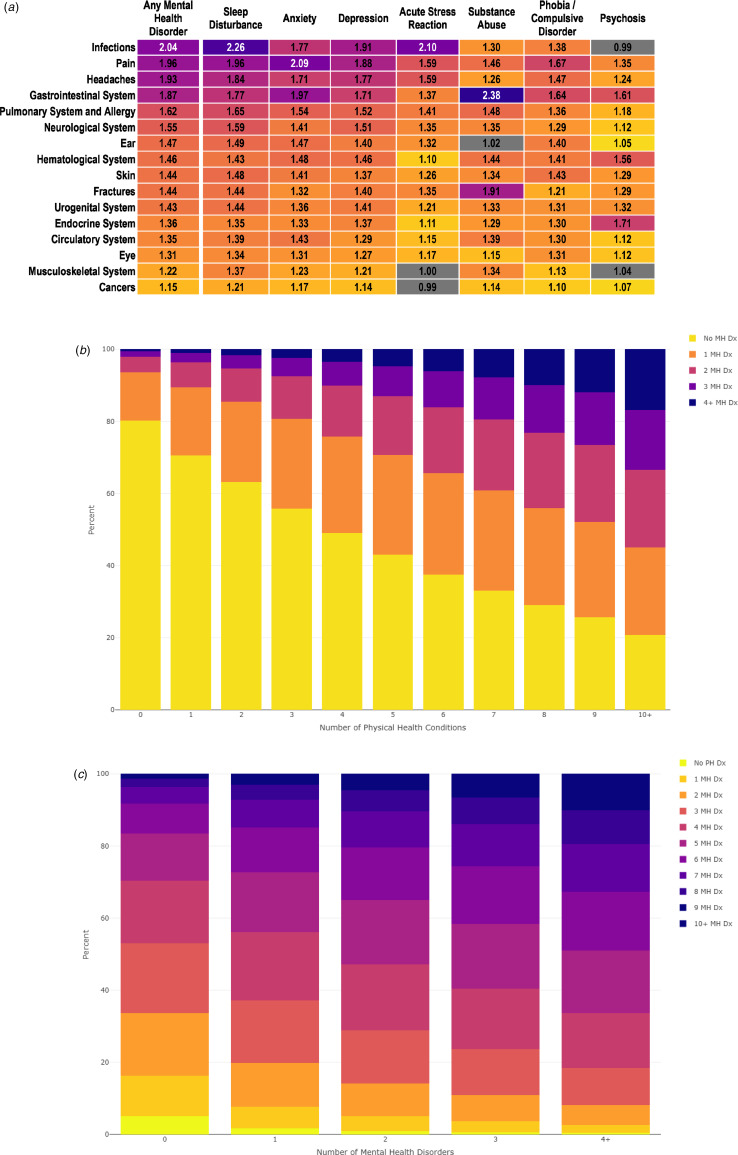
Associations between mental disorders and physical-health conditions. Panel A shows a heatmap of associations (odds ratios) between seven specific mental disorders and 16 physical-health conditions across a 14-year period in primary-care patients. The figure is organized from the strongest (top) to the weakest (bottom) associations with any mental health disorder. Inverse probability weighting was used to balance on four demographic variables: age at baseline, sex assigned at birth, educational attainment, and county of residence. The odds ratios shaded in grey are not statistically significant (i.e. the 99.99% confidence interval includes 1.0). All other odds ratios were statistically significant (i.e. 99.99% confidence interval does not include 1.0.). Panels B and C show multimorbidity associations between variety of different mental health disorders and variety of different multiple physical health conditions. Exact percentages are shown in online Supplementary Tables S7–S8. Inverse probability weighting was used to balance on four demographic variables: age at baseline, sex assigned at birth, educational attainment, and county of residence. (A) Heatmap of associations (odds ratios) between seven specific mental disorders and 16 physical-health conditions across a 14-year period in primary-care patients. (B) Multimorbidity associations between variety of different physical health conditions and variety of different mental health disorders. (C) Multimorbidity associations between variety of different mental health disorders and variety of different multiple physical health conditions. MH, mental health; PH, physical health.

We next tested associations among all 112 dyadic pairs of mental disorders and physical-health conditions (7 mental disorders × 16 physical-health conditions): 96% of the associations were positive and significant (ORs ranging from 1.05 [99.99% CI 1.00–1.09] to 2.38 [99.99% CI 2.30–2.46]) (Fig. 2a). The columns in Fig. 2a show that every specific mental disorder was associated with multiple different physical-health conditions (see also online Supplementary Figs S1–S7). Robustness analyses revealed that the associations between mental disorders and physical-health conditions were statistically significant, directionally consistent (positive), and exhibited a similar rank order among both men and women (online Supplementary Figs S8–S9, Table S4), younger and older adults (online Supplementary Figs S10–S11, Table S5), and among patients with and without a university education (online Supplementary Figs S12–S13, Table S6).

Analyses of multimorbidity revealed that mental-health disorders and physical conditions were tightly linked among primary-care patients (Fig. 2b and 2c, online Supplementary Tables S7–S8). Primary-care patients experiencing a greater variety of mental health disorders were much more likely to experience multiple physical conditions (*r* = 0.21). For example, among primary-care patients without a mental disorder 47% were likely to present with four or more physical conditions during the 14-year observation period. In contrast, among primary-care patients experiencing four or more mental disorders, the vast majority (82%) were likely to present with four or more physical conditions during the 14-year observation period.

**Figure 3.**
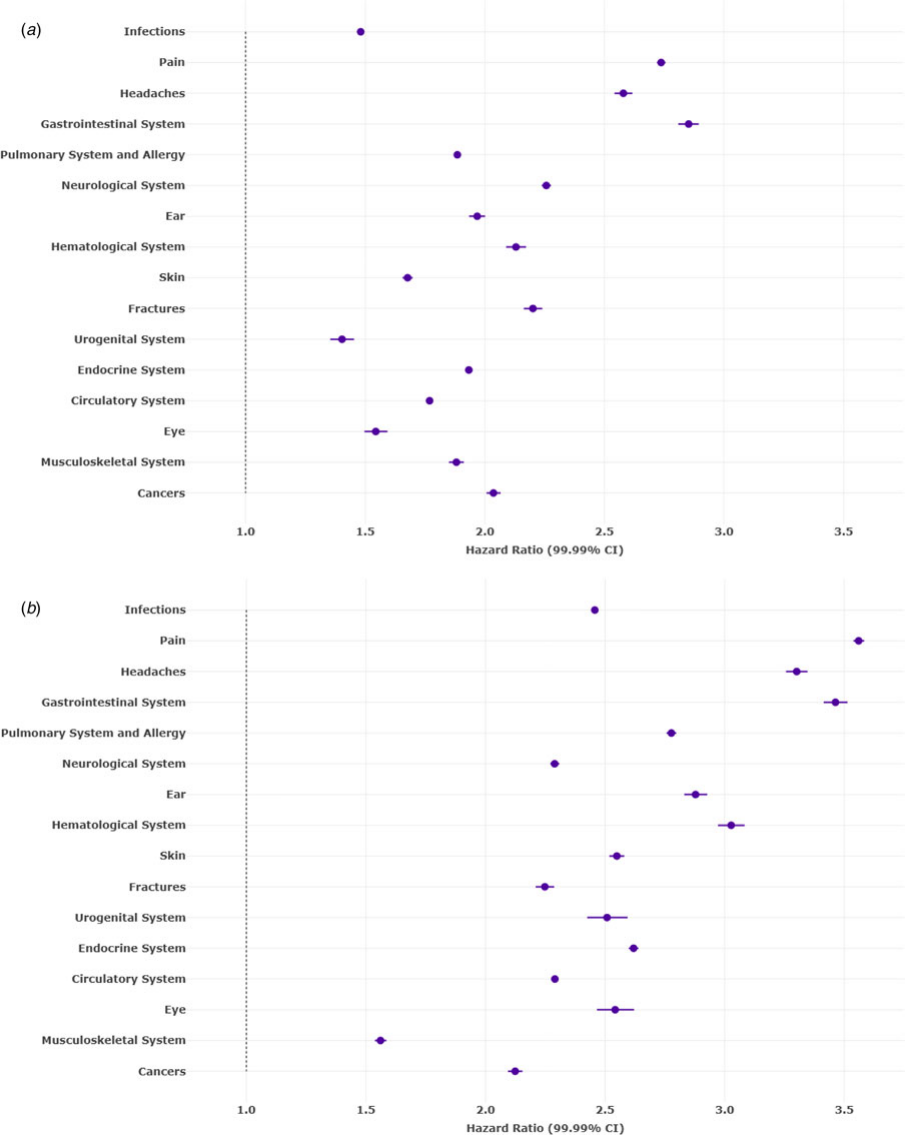
Bidirectional associations between any mental disorder and 16 physical-health conditions among primary-care patients across a 14-year observation period. Panel A shows the risk (hazard ratios) of 16 physical health conditions after a diagnosis of any mental health disorder and panel B shows the risk (hazard ratios) of any mental disorder after a diagnosis of 16 physical-health conditions. Inverse probability weights were used to balance on four demographic variables: sex, age, educational attainment, and county of residence. Within each month from January 2006 through December 2019, encounters with primary-care providers were assessed for mental health disorders and physical health conditions. Extended Cox proportional hazards models were used, treating dependent time-to-event variables and independent exposures as recurrent and time-varying, respectively. Results for specific mental disorders are shown in online Supplementary Figs S14–S15. (A) Risk of 16 physical-health conditions after any mental disorder. (B) Risk of any mental disorder after 16 physical-health conditions.

Longitudinal analyses documented that mental disorders and physical-health conditions were tightly linked bidirectionally over the 14-year observation window. Patients presenting with any mental disorder were at increased risk of subsequently presenting with each of the 16 different physical-health conditions assessed (Fig. 3a). Every specific mental disorder was significantly associated with an increased risk of subsequently presenting with multiple different physical-health conditions (online Supplementary Fig. S14). Conversely, patients presenting to PCPs with each of the 16 different physical-health conditions were at increased risk of subsequently presenting with a mental disorder (Fig. 3b). All physical-health conditions were associated with an increased risk of subsequently presenting with every specific mental disorder (online Supplementary Fig. S15).

**Figure 4.**
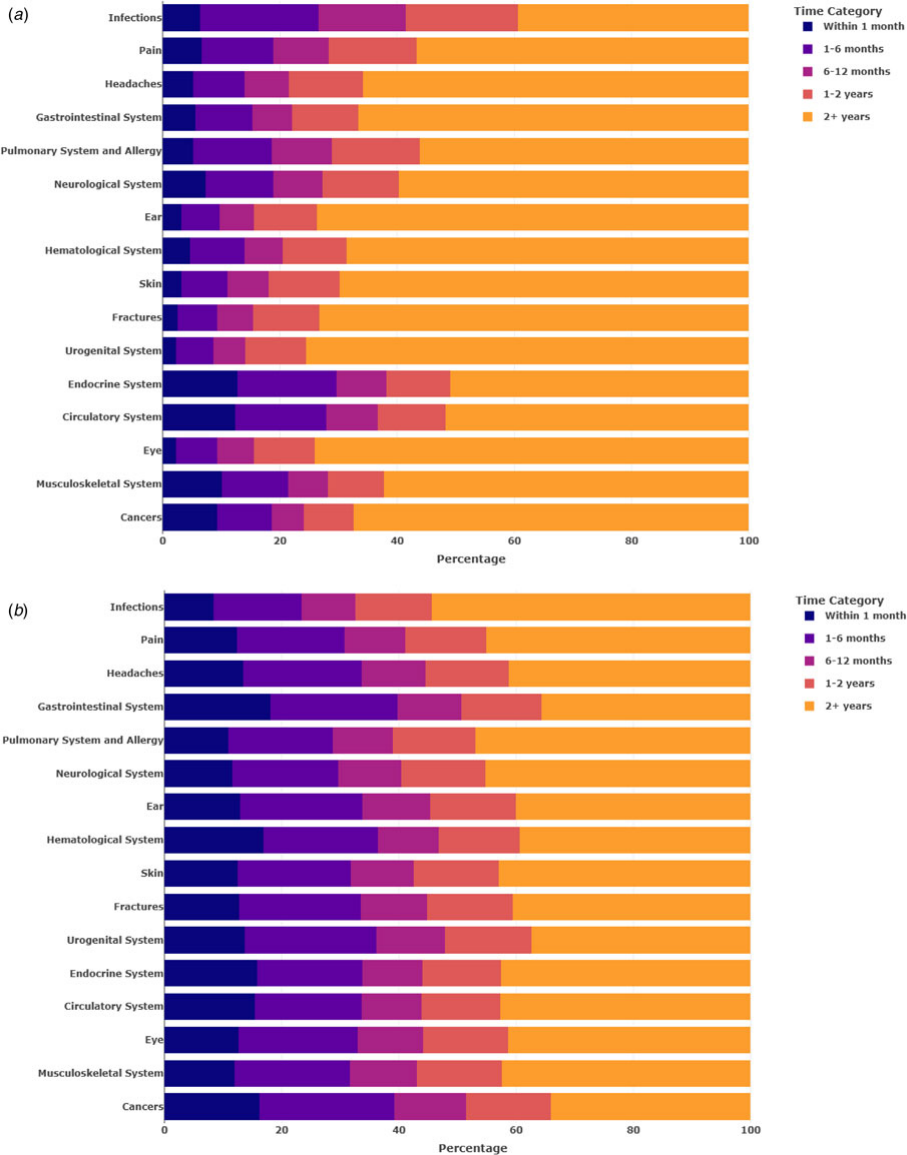
Evaluating surveillance bias in the association between any mental disorder and 16 physical-health conditions among primary-care patients. Panel A shows the distribution of time from first observed diagnosis of any mental disorder to first subsequent diagnosis of 16 physical-health conditions over the 14-year period. Panel B shows the distribution of time from first observed diagnosis of 16 physical-health conditions to first subsequent diagnosis of any mental disorder over the 14-year period. Exact percentages are shown in online Supplementary Tables S9–S10. (A) Timing of 16 physical-health conditions after any mental disorder. (B) Timing of any mental disorder after 16 physical-health conditions.

We next examined whether co-occurrence merely represented surveillance bias, in which mental-disorder and physical-health diagnoses were made very close to each other in time. However, findings suggested that mental disorders and physical-health conditions were linked over time for reasons other than surveillance bias. The increased risk of physical-health conditions after any mental-disorder diagnosis was not confined to the period immediately following the presentation of a mental-disorder diagnosis (Fig. 4a, online Supplementary Table S9); it extended over the entire 14-year period. The majority (15/16) of physical-health conditions were diagnosed at least 2 years after a mental-disorder diagnosis. Conversely, the increased risk of mental disorder after a physical-health condition extended over the entire 14-year window and over 50% of patients with a physical-health condition presented with a mental disorder more than a year after they were diagnosed with a mental disorder (Fig. 4b, online Supplementary Table S10).

To summarize the longitudinal data, Table 1 documents the physical-health histories of patients who presented in primary-care with a mental disorder during the mid-point (January 2012–December 2013) of our 14-year observation window. Patients presenting with any mental disorder in primary care were more likely than patients without any mental disorder to have a physical-health condition whether before, at the same time as, or after their mental-disorder diagnosis. This increased rate of physical-health conditions was observed across different health conditions and up to 5 years before and after the mental-disorder diagnosis.

**Table 1.** Rates of 16 physical-health conditions in primary-care patients with and without any mental disorder at three time points relative to an index date: 5, 3, and 1 year(s) before and 1, 3, and 5 year(s) after

	Time from index date													
	−5 Years		−3 Years		−1 Year		0		+1 Year		+3 Years		+5 Years	
Physical health condition	Without	With	Without	With	Without	With	Without	With	Without	With	Without	With	Without	With
Infections	21.3	33.8	21.3	34.6	22.1	35.2	21.8	34.9	19.7	32.6	20.1	31.9	11.5	19.7
Pain	6.6	15.0	6.7	15.5	7.4	17.4	7.6	18.3	7.6	17.9	8.1	18.5	4.8	12.0
Headaches	1.6	4.0	1.5	3.9	1.6	4.0	1.5	4.2	1.5	4.0	1.5	3.9	0.9	2.3
Gastrointestinal system	1.0	2.3	1.1	2.4	1.2	2.6	1.2	2.9	1.3	2.8	1.4	2.9	1.0	2.0
Pulmonary system and allergy	7.8	13.0	8.4	13.8	8.8	14.7	9.1	15.0	9.2	15.2	9.9	16.0	5.6	10.1
Neurological system	4.7	8.1	5.0	8.7	5.1	8.9	5.2	9.2	5.3	9.5	5.5	9.9	3.8	6.9
Ear	1.4	2.3	1.5	2.5	1.8	3.0	1.8	3.2	1.9	3.1	2.1	3.2	1.2	2.0
Hematological system	0.8	1.6	0.9	1.6	0.9	1.7	0.9	1.9	1.0	1.9	1.1	2.1	0.7	1.4
Skin	3.2	4.9	3.3	5.2	3.6	5.6	3.7	5.9	3.9	6.2	4.3	6.7	2.6	4.1
Fractures	1.4	2.4	1.5	2.5	1.6	2.7	1.7	2.8	1.6	2.8	1.8	2.9	1.1	1.9
Urogenital system	0.3	0.5	0.4	0.5	0.5	0.6	0.5	0.7	0.7	0.7	0.8	0.8	0.6	0.6
Endocrine system	3.9	5.7	4.7	6.7	5.3	7.6	5.6	8.2	6.1	8.5	6.9	9.5	5.8	7.9
Circulatory system	8.8	9.9	10.6	11.7	12.2	13.3	13.1	14.7	13.8	14.7	15.3	16.0	11.9	12.2
Eye	0.7	1.0	0.6	0.9	0.7	1.0	0.7	1.0	0.7	1.1	0.8	1.1	0.5	0.7
Musculoskeletal system	1.1	1.4	1.2	1.6	1.4	1.8	1.5	1.9	1.6	2.1	1.9	2.4	1.5	1.9
Cancers	1.1	1.1	1.3	1.4	1.6	1.9	1.8	2.3	2.0	2.5	2.5	3.0	2.0	2.3

For patients with a mental disorder (*N* = 1 067 183), we selected those (*N* = 421 304) who presented with a mental disorder between January 2012 and December 2013. The mental disorder closest to December 2012 (the middle of the observation window) was used as the index date. For patients without a mental disorder (*N* = 1 136 370), the index date was set to December 2012. Patients were counted as having a physical-health condition during the given time-windows if they had a physical health condition within 6 months before to 6 months after that time point.

## Discussion

In a comprehensive assessment of all primary-care medical encounters in Norway from 2006 to 2019, we observed positive bidirectional associations across all examined mental disorders and all examined physical-health conditions, illuminating the breadth of interconnectedness of mental and physical health. Patients diagnosed with a mental disorder were more likely to experience physical-health conditions over the 14-year observation period, and vice versa. These associations were similar among men and women, in younger and older adults, and also among patients who did and did not attend university. Thus, the co-occurrence was not concentrated among women, older adults, or low-education groups.

Although all mental disorders were associated with all physical-health conditions, infections, pain, headaches, gastrointestinal conditions, pulmonary conditions, and neurological conditions had the strongest associations across multiple mental disorders, rather than being specifically linked with any single mental disorder. We expected the strongest links between psychosis and physical-health conditions, as reported in prior research on multimorbidity in schizophrenia and bipolar disorder (Halstead et al., 2024). However, in our data the psychosis associations were not dissimilar to those of other mental disorders, with the exception of a stronger association between psychosis and endocrine conditions, a link consistent with prior research showing higher risk of endocrine-related adverse drug reactions associated with antipsychotic use (Firth et al., 2019; Knegtering, Van Der Moolen, Castelein, Kluiter, & Van Den Bosch, 2003; Peuskens, Pani, Detraux, & De Hert, 2014). The similarity between psychosis's and other mental disorders' associations with physical-health conditions is surprising considering the well-documented higher rates of morbidity and premature mortality linked to psychosis (De Hert et al., 2011; Firth et al., 2019; Hayes, Marston, Walters, King, & Osborn, 2017; Hjorthøj, Stürup, McGrath, & Nordentoft, 2017; O'Connor et al., 2023; Olfson, Gerhard, Huang, Crystal, & Stroup, 2015; Stubbs et al., 2016). One possibility is that psychosis is initially recognized in primary care while patients are yet young and relatively healthy, but psychosis patients are swiftly referred to specialist care where their later-emerging physical-health conditions are registered. However, in Norway's healthcare system PCP engagement is the norm for patients with schizophrenia (Hetlevik, Solheim, & Gjesdal, 2015). A second possibility is overshadowing of physical-health conditions in diagnostic practice by more pressing psychotic symptoms. There is evidence that patients with schizophrenia are likely to have physical-health conditions recorded at the time of death (Brink et al., 2019; Heiberg et al., 2019), suggesting missed physical-health conditions that are omitted in primary-care records.

This nationwide registry-based study had several strengths. First, the study offers a view of mental–physical comorbidities that is representative of the entire population of a nation. All residents in Norway are assigned a PCP and PCPs are generally the first point of contact in the healthcare system, even when contact concerns mental health (e.g. 97.3% of patients in Norway with psychiatric codes in specialist health care also had an ICPC-2 ‘psychological’ code in primary care). Second, in contrast to hospital registry data, which mainly capture severe cases, primary-care data provide a more representative view of co-occurring mental disorders and physical-health conditions that are encountered in healthcare systems (e.g. 77.5% of mental-health patients in Norway with mental-health codes in primary care did not appear in the specialist-care register). As such, the present study, which uses primary-care data rather than hospital-treatment data, represents a design complement to initial research based on hospital registry data (Momen et al., 2020, 2024). The convergence of findings from unique and complementary nationwide resources is a critical part of building a cumulative evidence base about the co-occurrence of mental and physical disorders. Third, we considered the possibility that the observed associations are an artifact of increased surveillance or healthcare utilization. However, within the observation window, most patients received their first physical-health diagnosis at least 2 years after their mental-disorder diagnosis and their mental-disorder diagnosis at least 1 year after their physical-health diagnosis. Furthermore, we observed no significant elevation in the rates of physical-health conditions within the 1-year window around the mental-disorder diagnosis date (index date). The absence of such a time trend in our data further supports the notion that the observed associations between mental disorders and physical-health conditions are not due to increased surveillance or healthcare utilization. Fourth, by applying covariates at the individual level, we could rule out the possibility that the accumulation of diagnoses was due to demographic characteristics associated with health and health service utilization, like gender, age, and educational attainment.

The study also had limitations. First, the study's use of the Norwegian healthcare register raises questions about the generalizability of our findings to countries with different healthcare infrastructures and population characteristics. However, co-occurrence of mental disorders and physical-health conditions has been reported from multiple countries in the WMH survey (Scott et al., 2016). Furthermore, while Norway's healthcare system may be dissimilar to that of the USA, it more closely resembles the healthcare systems of many OECD nations (Wendt, 2014). Second, the classification of mental disorders is challenging in primary care (Lamberts, Magruder, Kathol, Pincus, & Okkes, 1998; Torvik et al., 2018). Inter-doctor and inter-practice variation influences data precision, and limited time with patients may lead to misclassification. ICPC-2, like other mental-health diagnostic systems, is imperfect (e.g. it combines phobia and compulsive disorder into one code). However, it has face validity to patients seeking care and to providers who report these codes. Moreover, comparisons of mental-disorder diagnoses made by Norwegian PCPs and by clinical psychologists administering psychiatric interviews reveal the same etiological risk factors (Lamberts et al., 1998; Torvik et al., 2018). Third, ICPC-2 psychological codes capture the reason the patient is seeking medical care. They should not be interpreted as prevalence rates of mental-health conditions in the population, but as population-level estimates of service contacts for mental health. Fourth, the classification of diagnoses into groups of physical conditions (e.g. pulmonary, neurological, hematological) results in heterogeneous categories, but this classification, which follows previous research (Momen et al., 2020), provides a useful way to test the links between different mental disorders and physical conditions across multiple body systems. Fifth, our ability to detect bidirectional associations was limited by the 14-year observation window. While substantial, this window is insufficient for identifying incident cases as both mental disorders and physical-health conditions may have onset prior to the observation window and slow-progressing or age-related diseases may not have had time to emerge by the end of the window. The ideal design for estimating precedence in mental–physical comorbidity would follow patients from birth to death. Here we can only lay claim to the observation that in any given window from age 20 to 74, multiple mental disorders and multiple physical-health conditions co-occur to a significant degree. Moreover, it is possible that the associations between some physical diseases and subsequent mental disorders may arise as a byproduct of medication. This study is thus descriptive and does not imply causation; instead, it draws attention to the broad overlap between mental disorders and physical-health conditions among primary-care patients.

The ubiquity of mental–physical comorbidities underscores the necessity of medical training that equips PCPs with the knowledge to recognize and address the coexistence of mental and physical-health problems in their patients. PCPs must understand the interconnectedness of mental and physical health, as patients suffering from physical-health conditions often simultaneously face mental-health challenges that are linked to reduced treatment adherence and help-seeking (DiMatteo et al., 2000; Prince et al., 2007), including lower rates of physical examinations (Burns & Cohen, 1998; Hippisley-Cox, Parker, Coupland, & Vinogradova, 2007), preventive screenings, intervention (Liu et al., 2017; Saxena & Maj, 2017), and access to conventional healthcare resources (Firth et al., 2019). This understanding underscores the need for a proactive, integrated approach in primary care, where routine monitoring of both mental disorders and physical-health conditions would allow PCPs to mitigate treatment barriers, improving healthcare delivery and patient outcomes (Attoe, Lillywhite, Hinchliffe, Bazley, & Cross, 2018; Coventry et al., 2015; Das, Naylor, & Majeed, 2016; De Hert et al., 2011; Ee et al., 2020; Liu et al., 2017; O'Connor et al., 2023; Saxena & Maj, 2017).

The consistent and robust bidirectional associations observed across mental disorders and physical-health conditions also warrant a reevaluation of established etiological frameworks. Existing models, such as the inflammation hypothesis (Felger & Lotrich, 2013; Miller et al., 2009; Raison et al., 2006), shed light on the connections between specific mental disorders (e.g. depression) and physical-health conditions (e.g. cardiovascular disease) (Celano & Huffman, 2011; Dhar & Barton, 2016; Hare et al., 2014; Lett et al., 2004; Nemeroff & Goldschmidt-Clermont, 2012; Nicholson et al., 2006; Penninx, 2017). However, our study found that depression is not merely linked with cardiovascular disease but is also strongly associated with many other disease conditions across different body systems. Similarly, cardiovascular diseases of the circulatory system were associated with multiple mental disorders, not just depression (Chen et al., 2023). Thus, research should uncover what genetic, physiological, or lifestyle factors can account for broad associations among mental and physical conditions. In addition, the observed associations were consistently bidirectional. Research might investigate if the inflammation hypothesis extends to explain associations observed between cardiovascular disease and compulsive disorder, sleep, and anxiety, as well as depression, or between depression and pain, infections, headache, gastrointestinal, neurological, and pulmonary conditions, as well as cardiovascular conditions. While different etiological mechanisms may connect different mental–physical dyads, mechanistic research focusing solely on specific dyads overlooks the broader picture of how brain and body are linked.

## Conclusion

Mental disorders tend to co-occur with physical-health conditions in the same primary-care patients, irrespective of demographic characteristics. This finding underscores the important role of PCPs in addressing the co-occurrence of mental disorders and physical-health conditions, highlighting the need for medical education and care approaches that integrate mental and physical health. In addition, the breadth of associations across mental disorders and physical-health conditions raises new questions for etiological models derived from focusing on mental–physical dyads, and calls for research to explain the breadth of mental–physical health comorbidities.

## Supporting information

Hanna et al. supplementary material Hanna et al. supplementary material

## Data Availability

The data for this study are primary-care records for the Norwegian population. Researchers can access the data by application to the Regional Committees for Medical and Health Research Ethics and the data owners (Statistics Norway and the Norwegian Directorate of Health). The authors cannot share these data with other researchers. However, other researchers can contact the authors if they have questions concerning the data.

## Supplementary material

The supplementary material for this article can be found at https://doi.org/10.1017/S0033291724002575.
