# Supplementary material for: Co-occurrence between mental disorders and physical diseases: a study of nationwide primary-care medical records
Source: Psychol Med. 2024 Nov 18;54(15):4274–86. doi: 10.1017/S0033291724002575 (PMC11650190; doi:10.1017/S0033291724002575)
Supplement: Hanna et al. supplementary material [file S0033291724002575sup001.docx]

**Supplemental Material to accompany Hanna, et al.**

**Co-occurrence between mental disorders and physical diseases:**

**A study of nationwide primary-care medical records**

**Table of Contents**

[**Table S1. ICPC-2 Codes for Mental disorders.** 3](#_Toc177563425)

[**Table S2: Diagnostic codes for physical-health conditions** 4](#_Toc177563426)

[**Table S3. Prevalence of seven mental disorders and 16 physical-health conditions in primary care patients across 14 years** 14](#_Toc177563427)

[**Table S4. Associations between any mental disorder and 16 physical conditions by sex (odds ratios and 99.99% CI)** 15](#_Toc177563428)

[**Table S5. Associations between any mental disorder and 16 physical conditions by age (odds ratios and 99.99% CI)** 16](#_Toc177563429)

[**Table S6. Associations between any mental disorder and 16 physical conditions by education (odds ratios and 99.99% CI)** 17](#_Toc177563430)

[**Table S7. Multimorbidity: Number of different mental health disorders as a function of the number of different physical health conditions** 18](#_Toc177563431)

[**Table S8. Multimorbidity: Number of different physical health conditions as a function of the number of different mental health disorders.** 19](#_Toc177563432)

[**Table S9. Time from first observed diagnosis of any mental disorder to first subsequent diagnosis of physical health conditions.** 20](#_Toc177563433)

[**Table S10. Time from first observed diagnosis of a physical health condition to first subsequent diagnosis of a mental health disorder** 21](#_Toc177563434)

[**Figure S1. Associations between sleep disturbance and 16 physical-health conditions.** 22](#_Toc177563435)

[**Figure S2. Associations between anxiety and 16 physical-health conditions** 23](#_Toc177563436)

[**Figure S3. Associations between depression and 16 physical-health conditions.** 24](#_Toc177563437)

[**Figure S4. Associations between acute stress reaction and 16 physical-health conditions.** 25](#_Toc177563438)

[**Figure S5. Associations between substance abuse and 16 physical-health conditions** 26](#_Toc177563439)

[**Figure S6. Associations between phobia/compulsive disorder and 16 physical-health conditions** 27](#_Toc177563440)

[**Figure S7. Associations between psychosis and 16 physical-health conditions** 28](#_Toc177563441)

[**Figure S8. Associations (odds ratios) between any mental disorder and 16 physical-health conditions across a 14-year period in male and female primary-care patients.** 29](#_Toc177563442)

[**Figure S9. Associations (odds ratios) between seven specific mental disorders and 16 physical-health conditions across a 14-year period in male and female primary-care patients.** 30](#_Toc177563443)

[**Figure S10. Associations (odds ratios) between any mental disorders and 16 physical-health conditions across a 14-year period in younger and older (20-40 years vs 40-60 years at baseline) primary-care patients.** 34](#_Toc177563444)

[**Figure S11. Associations (odds ratios) between seven specific mental disorders and 16 physical-health conditions across a 14-year period in younger and older (20-40 years vs 40-60 years at baseline) primary-care patients.** 35](#_Toc177563445)

[**Figure S12. Associations (odds ratios) between any mental disorder and 16 physical-health conditions across a 14-year period in primary-care patients who did and did not attend college.** 39](#_Toc177563446)

[**Figure S13. Associations (odds ratios) between seven specific mental disorders and 16 physical-health conditions across a 14-year period in primary-care patients who did and did not attend college.** 40](#_Toc177563447)

[**Figure S14. Risk (hazard ratios) of 16 physical-health conditions after diagnosis of seven specific mental disorders across a 14-year period in primary-care patients.** 44](#_Toc177563448)

[**Figure S15. Risk (hazard ratios) of seven specific mental disorders after diagnosis of 16 physical-health conditions across a 14-year period in primary-care patients.** 48](#_Toc177563449)

[**References** 52](#_Toc177563450)

**Table S1. ICPC-2 Codes for Mental disorders.** This table shows the mental disorders studied along with their associated ICPC-2 condition names and codes. This information comes from the ICPC-2 chapter P (Psychological). For mental disorders, we focused on mental disorders with a prevalence of 2.5% or higher to ensure robust statistical analysis: sleep disturbance (18.9%), anxiety (13.4%), depression (25.6%), acute stress reaction (21.2%), substance abuse (6.8%), phobia/compulsive disorder (2.7%), and psychosis (2.7%).

| **Mental disorder** | **ICPC-2 Condition** | **ICPC-2 Code** |
| --- | --- | --- |
| Sleep Disturbance | Sleep disturbance | P06 |
| Anxiety | Feeling anxious/nervous/tense | P01 |
|  | Anxiety disorder/anxiety state | P74 |
| Depression | Feeling depressed | P03 |
|  | Depressive disorder | P76 |
| Acute Stress Reaction | Acute stress reaction | P02 |
| Substance Abuse | Chronic alcohol abuse | P15 |
|  | Acute alcohol abuse | P16 |
|  | Tobacco abuse | P17 |
|  | Medication abuse | P18 |
|  | Drug abuse | P19 |
| Phobia/compulsive disorder | Phobia/compulsive disorder | P79 |
| Psychosis | Organic psychosis other | P71 |
|  | Schizophrenia | P72 |
|  | Affective psychosis | P73 |
|  | Psychosis NOS/other | P98 |

**Table S2: Diagnostic codes for physical-health conditions**. This table shows the physical-health conditions studied and their diagnostic codes in both the ICD-10 and ICPC-2 coding systems. For physical-health conditions, we focused on nine disease categories previously reported in a nationwide hospital registry study of mental disorders and physical diseases^1^. Because previous registry research used ICD-10 codes, we used a Norwegian Directorate of eHealth mapping system^2^ to convert these ICD-10 codes into their corresponding ICPC-2 codes. In addition to these nine disease categories, we report seven additional physical-health conditions: infections, pain, headaches, fractures, skin, ear and eye conditions, which were defined directly from their respective ICPC-2 codes. In total, our study encompassed 16 total physical-health conditions, represented by centered subtitles, which comprise individual physical-health conditions listed underneath.

| **Physical-health condition** | **ICD-10 Codes** | **ICPC-2 Condition** | **ICPC-2 Code** |
| --- | --- | --- | --- |
| **Circulatory System** | | | |
| Hypertension | I10 | Hypertension uncomplicated | K86 |
|  | I11-I13, I15 | Hypertension complicated | K87 |
| Dyslipidemia | E78 | Lipid disorder | T93 |
| Ischemic heart disease | I20, I24 | Ischaemic heart disease with angina | K74 |
|  | I21-I24 | Acute myocardial infarction | K75 |
|  | I25 | Ischaemic heart disease without angina | K76 |
| Atrial fibrillation | I48 | Atrial fibrillation/flutter | K78 |
| Heart failure | I50 | Heart failure | K77 |
| Peripheral artery occlusive disease | I70, I73-I74 | Atherosclerosis/peripheral vascular disease | K92 |
| Stroke | I60-I64 | Stroke/cerebrovascular accident | K90 |
|  | I69 | Cerebrovascular disease | K91 |
| Valvular disease^3^ | I34-I37, I39 | Heart valve disease NOS | K83 |
| Pulmonary circulatory disease^3^ | I27-I28 | Pulmonary heart disease | K82 |
| Cardiac arrhythmia^3^ | I49 | Cardiac arrhythmia NOS | K80 |

| **Physical-health condition** | **ICD-10 Codes** | **ICPC-2 Condition** | **ICPC-2 Code** |
| --- | --- | --- | --- |
| **Endocrine System** | | | |
| Diabetes mellitus | E10 | Diabetes insulin dependent | T89 |
|  | E11-E14 | Diabetes non-insulin dependent | T90 |
| Thyroid disorder | E00 | Congenital anomaly endocrine/metabolic | T80 |
|  | E01-E03 | Hypothyroidism/myxoedema | T86 |
|  | E04 | Goitre | T81 |
|  | E05 | Hyperthyroidism/thyrotoxicosis | T85 |
| Gout | M10 | Gout | T92 |
| **Pulmonary System and Allergy** | | | |
| Chronic pulmonary disease | J40 | Acute bronchitis/bronchiolitis | R78 |
|  | J41-J42 | Chronic bronchitis | R79 |
|  | J43-J44 | Chronic obstructive pulmonary disease | R95 |
|  | J45-J46 | Asthma | R96 |
| Allergy | J30 | Allergic rhinitis | R97 |
|  | L23 | Dermatitis contact/allergic | S88 |
|  | T78.0, T78.2, T78.4 | Allergy/allergic reaction NOS | A92 |
| **Gastrointestinal System** | | | |
| Ulcer/chronic gastritis | K25, K27-K28 | Peptic ulcer other | D86 |
|  | K26 | Duodenal ulcer | D85 |
|  | K29.3, K29.5 | Stomach function disorder | D87 |
| Chronic liver disease | B16-B19 | Viral hepatitis | D72 |
|  | K70, K74, K76.6 | Liver disease NOS | D97 |
| Inflammatory bowel disease | K50, K51 | Chronic enteritis/ulcerative colitis | D94 |
| Diverticular disease of intestine | K57 | Diverticular disease | D92 |

| **Physical-health condition** | **ICD-10 Codes** | **ICPC-2 Condition** | **ICPC-2 Code** |
| --- | --- | --- | --- |
| **Urogenital System** | | | |
| Chronic kidney disease | N03 | Glomerulonephritis/nephrosis | U88 |
|  | N11 | Pyelonephritis/pyelitis | U70 |
| Prostate disorders | N40 | Benign prostatic hypertrophy | Y85 |
| **Musculoskeletal System** | | | |
| Connective tissue disorders | M05-M06, M08 | Rheumatoid/seropositive arthritis | L88 |
| Osteoporosis | M80-M82 | Osteoporosis | L95 |
| **Hematological System** | | | |
| HIV/AIDS | B20-B24 | HIV-infection/AIDS | B90 |
| Anemias | D50 | Iron deficiency anaemia | B80 |
|  | D51-D52 | Anaemia vit B12/folate deficiency | B81 |
|  | D53, D55, D59-D61, D63-D64 | Anaemia other/unspecified | B82 |
|  | D56-D58 | Hereditary haemolytic anaemia | B78 |
| Coagulopathy^3^ | D65-D69 | Purpura/coagulation defect | B83 |
| **Cancers** | | | |
| General & Unspecified | C38, C45, C76, C78-C80, C97 | Malignancy NOS | A79 |
| Blood, Blood Forming Organs & Immune Mechanism | C81-C86 | Hodgkin Lymphoma | B72 |
|  | C91-C95 | Leukaemia | B73 |
|  | C26, C37, C77, C88, C90, C94, C96 | Malignant neoplasm blood other | B74 |
| Digestive | C00-C08, C14-C15, C17, C22-C24, C26, C45, C48 | Malignant digestive neoplasm other/NOS | D77 |
|  | C16 | Malignant neoplasm stomach | D74 |
| **Physical-health condition** | **ICD-10 Codes** | **ICPC-2 Condition** | **ICPC-2 Code** |
| **Cancers, continued** | | | |
|  | C18-C21 | Malignant neoplasm colon/rectum | D75 |
|  | C25 | Malignant neoplasm pancreas | D76 |
| Eye | C69 | Neoplasm of eye/adnexa | F74 |
| Ear | C30, C49 | Neoplasm of ear | H75 |
| Cardiovascular | C38, C45 | Neoplasm cardiovascular | K72 |
| Musculoskeletal | C40-C41, C49 | Malignant neoplasm musculoskeletal | L71 |
| Neurological | C47, C70-C72 | Malignant neoplasm nervous system | N74 |
| Respiratory | C33-C34, C45 | Malignant neoplasm bronchus/lung | R84 |
|  | C09-C14, C31-C32, C38-C39, C45 | Malignant neoplasm respiratory other | R85 |
| Skin | C43, C46 | Malignant neoplasm of skin | S77 |
| Endocrine/Metabolic and Nutritional | C73 | Malignant neoplasm thyroid | T71 |
|  | C74 | Neoplasm endocrine other/unspecified | T73 |
| Urological | C64-C65 | Malignant neoplasm of kidney | U75 |
|  | C66, C68 | Malignant neoplasm urinary tract other | U77 |
|  | C67 | Malignant neoplasm of bladder | U76 |
| Pregnancy, Childbearing, Family Planning | C58 | Malignant neoplasm related to pregnancy | W72 |
|  | C50 | Malignant neoplasm breast female | X76 |
| Female Genital | C51, C54-C57 | Malignant neoplasm genital female other | X77 |
|  | C53 | Malignant neoplasm cervix | X75 |
| Male Genital | C61 | Malignant neoplasm prostate | Y77 |
|  | C50, C60, C62-C63 | Malignant neoplasm male genital other | Y78 |

| **Physical-health condition** | **ICD-10 Codes** | **ICPC-2 Condition** | **ICPC-2 Code** |
| --- | --- | --- | --- |
| **Neurological System** | | | |
| Migraine | G43 | Migraine | N89 |
| Epilepsy | G40-G41 | Epilepsy | N88 |
| Parkinson's disease | G20-G22 | Parkinsonism | N87 |
| Multiple schlerosis | G35 | Multiple sclerosis | N86 |
| Neuropathies | G50 | Trigeminal neuralgia | N92 |
|  | G51, G53 | Facial paralysis/bell's palsy | N91 |
|  | G54-G64 | Peripheral neuritis/neuropathy | N94 |
|  | G55 | Back syndrome with radiating pain | L86 |
|  | G56 | Carpal tunnel syndrome | N93 |
| Paralysis or paresis^3^ | G81-G83, G98 | Paralysis/weakness | N18 |
| **Infections** | | | |
| General & Unspecified |  | Tuberculosis | A70 |
|  |  | Measles | A71 |
|  |  | Chickenpox | A72 |
|  |  | Malaria | A73 |
|  |  | Rubella | A74 |
|  |  | Infectious mononucleosis | A75 |
|  |  | Viral exanthem other | A76 |
|  |  | Viral disease other/NOS | A77 |
|  |  | Infectious disease other/NOS | A78 |
| Blood, Blood Forming Organs & Immune Mechanism |  | Lymphadenitis acute | B70 |
|  |  | Lymphadenitis non-specific | B71 |
| Digestive |  | Gastrointestinal infection | D70 |
|  |  | Mumps | D71 |
|  |  | Gastroenteritis presumed infection | D73 |

| **Physical-health condition** | **ICD-10 Codes** | **ICPC-2 Condition** | **ICPC-2 Code** |
| --- | --- | --- | --- |
| **Infections, continued** | | | |
| Eye |  | Conjunctivitis infectious | F70 |
|  |  | Conjunctivitis allergic | F71 |
|  |  | Blepharitis/stye/chalazion | F72 |
|  |  | Eye infection/inflammation other | F73 |
| Ear |  | Otitis externa | H70 |
|  |  | Acute otitis media/myringitis | H71 |
|  |  | Serous otitis media | H72 |
|  |  | Eustachian salpingitis | H73 |
|  |  | Chronic otitis media | H74 |
| Cardiovascular |  | Infection of circulatory system | K70 |
|  |  | Rheumatic fever/heart disease | K71 |
| Musculoskeletal |  | Infections musculoskeletal system | L70 |
| Neurological |  | Poliomyelitis | N70 |
|  |  | Meningitis/encephalitis | N71 |
|  |  | Tetanus | N72 |
|  |  | Neurological infection other | N73 |
| Respiratory |  | Whooping cough | R71 |
|  |  | Strep throat | R72 |
|  |  | Boil/abscess nose | R73 |
|  |  | Upper respiratory infection acute | R74 |
|  |  | Sinusitis acute/chronic | R75 |
|  |  | Tonsillitis acute | R76 |
|  |  | Laryngitis/tracheitis acute | R77 |
|  |  | Influenza | R80 |
|  |  | Pneumonia | R81 |
|  |  | Pleurisy/pleural effusion | R82 |
|  |  | Respiratory infection other | R83 |

| **Physical-health condition** | **ICD-10 Codes** | **ICPC-2 Condition** | **ICPC-2 Code** |
| --- | --- | --- | --- |
| **Infections, continued** | | | |
| Skin |  | Warts | S03 |
|  |  | Infected finger/toe | S09 |
|  |  | Boil/carbuncle | S10 |
|  |  | Skin infection post-traumatic | S11 |
|  |  | Herpes zoster | S70 |
|  |  | Herpes simplex | S71 |
|  |  | Scabies/other acariasis | S72 |
|  |  | Pediculosis/skin infestation other | S73 |
|  |  | Dermatophytosis | S74 |
|  |  | Moniliasis/candidiasis skin | S75 |
|  |  | Skin infection other | S76 |
|  |  | Impetigo | S84 |
|  |  | Molluscum contagiosum | S95 |
| Endocrine/Metabolic & Nutritional |  | Endocrine infection | T70 |
| Urological |  | Cystitis/urinary infection other | U71 |
|  |  | Urethritis | U72 |
| Pregnancy, Childbearing, Family Planning |  | Puerperal infection/sepsis | W70 |
|  |  | Infection complicating pregnancy | W71 |
| Female Genital |  | Syphilis female | X70 |
|  |  | Gonorrhoea female | X71 |
|  |  | Genital candidiasis female | X72 |
|  |  | Genital trichomoniasis female | X73 |
|  |  | Pelvic inflammatory disease | X74 |
|  |  | Genital herpes female | X90 |
|  |  | Condylomata acuminata female | X91 |
|  |  | Chlamydia infection genital (f) | X92 |

| **Physical-health condition** | **ICD-10 Codes** | **ICPC-2 Condition** | **ICPC-2 Code** |
| --- | --- | --- | --- |
| **Infections, continued** | | | |
| Male Genital |  | Syphilis male | Y70 |
|  |  | Gonorrhoea male | Y71 |
|  |  | Genital herpes male | Y72 |
|  |  | Prostatitis/seminal vesiculitis | Y73 |
|  |  | Orchitis/epididymitis | Y74 |
|  |  | Balanitis | Y75 |
|  |  | Condylomata acuminata male | Y76 |
| **Pain** | | | |
| General & Unspecified |  | Pain general/multiple sites | A01 |
|  |  | Chest pain NOS | A11 |
| Blood, Blood Forming Organs & Immune Mechanisms |  | Lymph gland(s) enlarged/painful | B02 |
| Digestive |  | Abdominal pain/cramps general | D01 |
|  |  | Abdominal pain epigastric | D02 |
|  |  | Rectal/anal pain | D04 |
|  |  | Abdominal pain localized other | D06 |
| Eye |  | Eye pain | F01 |
| Ear |  | Ear pain/earache | H01 |
| Cardiovascular |  | Heart pain | K01 |
|  |  | Cardiovascular pain NOS | K03 |
| Muscle Pain |  | Muscle pain | L18 |
| Face Pain |  | Pain face | N03 |
| Respiratory |  | Pain respiratory system | R01 |
| Skin |  | Pain/tenderness of skin | S01 |
| Urological |  | Dysuria/painful urination | U01 |

| **Physical-health condition** | **ICD-10 Codes** | **ICPC-2 Condition** | **ICPC-2 Code** |
| --- | --- | --- | --- |
| **Pain, continued** | | | |
| Female Genital |  | Genital pain female | X01 |
|  |  | Menstrual pain | X02 |
|  |  | Intermenstrual pain | X03 |
|  |  | Painful intercourse female | X04 |
|  |  | Breast pain female | X18 |
| Male Genital |  | Pain in penis | Y01 |
|  |  | Pain in testis/scrotum | Y02 |
| **Headaches** | | | |
|  |  | Headache | N01 |
|  |  | Cluster headache | N90 |
|  |  | Tension headache | N95 |
| **Fractures** | | | |
|  |  | Fracture: radius/ulna | L72 |
|  |  | Fracture: tibia/fibula | L73 |
|  |  | Fracture: hand/foot bone | L74 |
|  |  | Fracture: femur | L75 |
|  |  | Fracture: other | L76 |
| **Skin** | | | |
|  |  | Pruritus | S02 |
|  |  | Pilonidal cyst/fistula | S85 |
|  |  | Dermatitis seborrhoeic | S86 |
|  |  | Dermatitis/atopic eczema | S87 |
|  |  | Pityriasis rosea | S90 |
|  |  | Psoriasis | S91 |
|  |  | Sweat gland disease | S92 |
|  |  | Sebaceous cyst | S93 |
|  |  | Ingrowing nail | S94 |
| **Physical-health condition** | **ICD-10 Codes** | **ICPC-2 Condition** | **ICPC-2 Code** |
| **Skin, continued** | | | |
|  |  | Acne | S96 |
|  |  | Chronic ulcer skin | S97 |
|  |  | Urticaria | S98 |
|  |  | Skin disease, other | S99 |
| **Ear** | | | |
|  |  | Hearing complaint | H02 |
|  |  | Tinnitus, ringing/buzzing ear | H03 |
|  |  | Ear discharge | H04 |
|  |  | Bleeding ear | H05 |
|  |  | Plugged feeling ear | H13 |
|  |  | Ear symptom/complaint other | H29 |
|  |  | Vertiginous Syndrome | H82 |
|  |  | Otosclerosis | H83 |
|  |  | Presbyacusis | H84 |
|  |  | Acoustic trauma | H85 |
|  |  | Ear/mastoid disease, other | H99 |
| **Eye** | | | |
|  |  | Red eye | F02 |
|  |  | Eye discharge | F03 |
|  |  | Detached retina | F82 |
|  |  | Retinopathy | F83 |
|  |  | Macular degeneration | F84 |
|  |  | Corneal ulcer | F85 |
|  |  | Trachoma | F86 |
|  |  | Refractive error | F91 |
|  |  | Cataract | F92 |
|  |  | Glaucoma | F93 |
|  |  | Eye/adnexa disease, other | F99 |

**Table S3. Prevalence of seven mental disorders and 16 physical-health conditions in primary care patients across 14 years**. Prevalence is shown for all primary care patients; females and males; younger patients (20-40 years old at baseline) and older patients (40-60 years old at baseline); and among adults with and without a college education.

|  | Overall | | Female | | Male | | 20-40y | | 40-60y | | College | | No College | |
| --- | --- | --- | --- | --- | --- | --- | --- | --- | --- | --- | --- | --- | --- | --- |
|  | N = 2,203,553 | | N = 1,082,286 | | N = 1,121,267 | | N = 1,065,449 | | N = 1,138,104 | | N = 693,646 | | N = 1,509,907 | |
|  | N | % | N | % | N | % | N | % | N | % | N | % | N | % |
| **Mental Health Conditions** |  |  |  |  |  |  |  |  |  |  |  |  |  |  |
| Any Mental Health Disorder | 1,101,415 | 50.0 | 635,415 | 58.7 | 466,000 | 41.6 | 561,224 | 52.7 | 540,191 | 47.5 | 798,793 | 52.9 | 302,622 | 43.6 |
| Sleep Disturbance | 417,276 | 18.9 | 240,212 | 22.2 | 177,064 | 15.8 | 186,491 | 17.5 | 230,785 | 20.3 | 309,912 | 20.5 | 107,364 | 15.5 |
| Anxiety | 294,281 | 13.4 | 180,204 | 16.7 | 114,077 | 10.2 | 156,307 | 14.7 | 137,974 | 12.1 | 227,912 | 15.1 | 66,369 | 9.6 |
| Depression | 563,891 | 25.6 | 344,608 | 31.8 | 219,283 | 19.6 | 300,531 | 28.2 | 263,360 | 23.1 | 413,153 | 27.4 | 150,738 | 21.7 |
| Acute Stress Reaction | 467,444 | 21.2 | 304,081 | 28.1 | 163,363 | 14.6 | 275,756 | 25.9 | 191,688 | 16.8 | 326,430 | 21.6 | 141,014 | 20.3 |
| Substance Abuse | 148,795 | 6.8 | 58,841 | 5.4 | 89,954 | 8.0 | 69,779 | 6.5 | 79,016 | 6.9 | 130,914 | 8.7 | 17,881 | 2.6 |
| Phobia / Compulsive Disorder | 59,796 | 2.7 | 32,973 | 3.0 | 26,823 | 2.4 | 38,313 | 3.6 | 21,483 | 1.9 | 46,637 | 3.1 | 13,159 | 1.9 |
| Psychosis | 65,213 | 3.0 | 32,437 | 3.0 | 32,776 | 2.9 | 34,265 | 3.2 | 30,948 | 2.7 | 51,881 | 3.4 | 13,332 | 1.9 |
| **Physical Health Conditions** |  |  |  |  |  |  |  |  |  |  |  |  |  |  |
| Infections | 1,886,370 | 85.6 | 982,140 | 90.7 | 904,230 | 80.6 | 941,513 | 88.4 | 944,857 | 83.0 | 1,293,993 | 85.7 | 592,377 | 85.4 |
| Pain | 1,210,609 | 54.9 | 677,775 | 62.6 | 532,834 | 47.5 | 588,184 | 55.2 | 622,425 | 54.7 | 868,248 | 57.5 | 342,361 | 49.4 |
| Headaches | 402,056 | 18.2 | 255,062 | 23.6 | 146,994 | 13.1 | 232,483 | 21.8 | 169,573 | 14.9 | 284,304 | 18.8 | 117,752 | 17.0 |
| Gastrointestinal System | 222,080 | 10.1 | 119,045 | 11.0 | 103,035 | 9.2 | 92,043 | 8.6 | 130,037 | 11.4 | 172,187 | 11.4 | 49,893 | 7.2 |
| Pulmonary System and Allergy | 1,057,977 | 48.0 | 584,080 | 54.0 | 473,897 | 42.3 | 514,883 | 48.3 | 543,094 | 47.7 | 743,970 | 49.3 | 314,007 | 45.3 |
| Neurological System | 691,552 | 31.4 | 385,872 | 35.7 | 305,680 | 27.3 | 316,213 | 29.7 | 375,339 | 33.0 | 516,872 | 34.2 | 174,680 | 25.2 |
| Ear | 433,265 | 19.7 | 239,669 | 22.1 | 193,596 | 17.3 | 178,614 | 16.8 | 254,651 | 22.4 | 307,074 | 20.3 | 126,191 | 18.2 |
| Hematological System | 188,051 | 8.5 | 136,665 | 12.6 | 51,386 | 4.6 | 92,042 | 8.6 | 96,009 | 8.4 | 129,732 | 8.6 | 58,319 | 8.4 |
| Skin | 702,023 | 31.9 | 390,276 | 36.1 | 311,747 | 27.8 | 345,337 | 32.4 | 356,686 | 31.3 | 491,961 | 32.6 | 210,062 | 30.3 |
| Fractures | 381,865 | 17.3 | 190,876 | 17.6 | 190,989 | 17.0 | 160,269 | 15.0 | 221,596 | 19.5 | 289,572 | 19.2 | 92,293 | 13.3 |
| Urogenital System | 110,054 | 5.0 | 42,758 | 4.0 | 67,296 | 6.0 | 29,667 | 2.8 | 80,387 | 7.1 | 81,665 | 5.4 | 28,389 | 4.1 |
| Endocrine System | 376,734 | 17.1 | 208,849 | 19.3 | 167,885 | 15.0 | 116,358 | 10.9 | 260,376 | 22.9 | 290,082 | 19.2 | 86,652 | 12.5 |
| Circulatory System | 812,074 | 36.9 | 369,331 | 34.1 | 442,743 | 39.5 | 200,555 | 18.8 | 611,519 | 53.7 | 626,977 | 41.5 | 185,097 | 26.7 |
| Eye | 189,588 | 8.6 | 106,519 | 9.8 | 83,069 | 7.4 | 64,308 | 6.0 | 125,280 | 11.0 | 136,675 | 9.1 | 52,913 | 7.6 |
| Musculoskeletal System | 131,449 | 6.0 | 94,089 | 8.7 | 37,360 | 3.3 | 30,655 | 2.9 | 100,794 | 8.9 | 102,046 | 6.8 | 29,403 | 4.2 |
| Cancers | 212,227 | 9.6 | 116,522 | 10.8 | 95,705 | 8.5 | 40,067 | 3.8 | 172,160 | 15.1 | 156,811 | 10.4 | 55,416 | 8.0 |

**Table S4. Associations between any mental disorder and 16 physical conditions by sex (odds ratios and 99.99% CI)**

|  | **Any Mental Health Disorder** | | **Sleep Disturbance** | | **Anxiety** | | **Depression** | | **Acute Stress Reaction** | | **Substance Abuse** | | **Phobia / Compulsive Disorder** | | **Psychosis** | |
| --- | --- | --- | --- | --- | --- | --- | --- | --- | --- | --- | --- | --- | --- | --- | --- | --- |
| **Physical Health Disorder** | **Females** | **Males** | **Females** | **Males** | **Females** | **Males** | **Females** | **Males** | **Females** | **Males** | **Females** | **Males** | **Females** | **Males** | **Females** | **Males** |
| Infections | 2.4  (2.4, 2.5) | 1.9  (1.8, 1.9) | 2.4  (2.3, 2.5) | 2.2  (2.2, 2.3) | 2.0  (1.9, 2.1) | 1.7  (1.6, 1.8) | 2.2  (2.1, 2.3) | 1.8  (1.8, 1.9) | 2.2  (2.1, 2.3) | 2.1  (2.0, 2.2) | 1.5  (1.4, 1.7) | 1.2  (1.2, 1.3) | 1.5  (1.4, 1.7) | 1.3  (1.2, 1.4) | 1.0  (0.9, 1.1) | 1.0  (0.9, 1.0) |
| Pain | 2.1  (2.1, 2.2) | 1.9  (1.8, 1.9) | 2.0  (2.0, 2.1) | 2.0  (1.9, 2.0) | 2.2  (2.2, 2.3) | 2.0  (2.0, 2.1) | 2.0  (2.0, 2.1) | 1.8  (1.8, 1.8) | 1.6  (1.6, 1.6) | 1.6  (1.6, 1.7) | 1.6  (1.5, 1.7) | 1.4  (1.3, 1.4) | 1.8  (1.7, 1.9) | 1.6  (1.5, 1.7) | 1.4  (1.4, 1.5) | 1.3  (1.2, 1.4) |
| Headaches | 2.0  (1.9, 2.0) | 1.9  (1.9, 2.0) | 1.8  (1.7, 1.8) | 2.0  (2.0, 2.1) | 1.7  (1.6, 1.7) | 1.8  (1.8, 1.9) | 1.8  (1.7, 1.8) | 1.8  (1.8, 1.9) | 1.6  (1.5, 1.6) | 1.7  (1.6, 1.7) | 1.3  (1.2, 1.3) | 1.2  (1.2, 1.3) | 1.4  (1.4, 1.5) | 1.6  (1.5, 1.7) | 1.2  (1.2, 1.3) | 1.2  (1.2, 1.3) |
| Gastrointestinal System | 1.8  (1.7, 1.8) | 2.0  (2.0, 2.1) | 1.7  (1.6, 1.7) | 1.9  (1.8, 1.9) | 1.8  (1.8, 1.9) | 2.1  (2.1, 2.2) | 1.6  (1.6, 1.7) | 1.8  (1.8, 1.9) | 1.3  (1.2, 1.3) | 1.5  (1.4, 1.5) | 2.2  (2.1, 2.3) | 2.6  (2.5, 2.7) | 1.6  (1.5, 1.7) | 1.7  (1.6, 1.8) | 1.5  (1.4, 1.6) | 1.7  (1.6, 1.8) |
| Pulmonary System & Allergy | 1.7  (1.6, 1.7) | 1.6  (1.6, 1.6) | 1.6  (1.6, 1.6) | 1.7  (1.7, 1.7) | 1.6  (1.5, 1.6) | 1.5  (1.5, 1.6) | 1.5  (1.5, 1.6) | 1.5  (1.5, 1.5) | 1.4  (1.4, 1.4) | 1.4  (1.4, 1.5) | 1.7  (1.6, 1.8) | 1.3  (1.3, 1.4) | 1.4  (1.3, 1.5) | 1.4  (1.3, 1.4) | 1.2  (1.2, 1.3) | 1.1  (1.1, 1.2) |
| Neurological System | 1.6  (1.6, 1.6) | 1.5  (1.5, 1.6) | 1.6  (1.6, 1.6) | 1.6  (1.6, 1.6) | 1.4  (1.4, 1.4) | 1.4  (1.4, 1.5) | 1.5  (1.5, 1.5) | 1.5  (1.5, 1.6) | 1.3  (1.3, 1.3) | 1.4  (1.4, 1.4) | 1.4  (1.3, 1.4) | 1.3  (1.3, 1.4) | 1.3  (1.3, 1.4) | 1.3  (1.2, 1.3) | 1.1  (1.1, 1.2) | 1.1  (1.0, 1.2) |
| Ear | 1.5  (1.4, 1.5) | 1.5  (1.4, 1.5) | 1.4  (1.4, 1.5) | 1.6  (1.5, 1.6) | 1.5  (1.4, 1.5) | 1.5  (1.4, 1.5) | 1.4  (1.3, 1.4) | 1.4  (1.4, 1.5) | 1.3  (1.2, 1.3) | 1.4  (1.3, 1.4) | 1.0  (1.0, 1.1) | 1.0  (0.9, 1.0) | 1.4  (1.3, 1.5) | 1.4  (1.3, 1.5) | 1.0  (1.0, 1.1) | 1.0  (1.0, 1.1) |
| Hematological System | 1.4  (1.3, 1.4) | 1.8  (1.7, 1.8) | 1.3  (1.3, 1.4) | 1.7  (1.7, 1.8) | 1.4  (1.3, 1.4) | 1.8  (1.7, 1.8) | 1.4  (1.4, 1.4) | 1.7  (1.6, 1.8) | 1.1  (1.1, 1.1) | 1.1  (1.1, 1.2) | 1.2  (1.1, 1.3) | 2.1  (1.9, 2.2) | 1.3  (1.2, 1.4) | 1.7  (1.5, 1.9) | 1.4  (1.3, 1.4) | 2.1  (1.9, 2.3) |
| Skin | 1.4  (1.4, 1.5) | 1.4  (1.4, 1.5) | 1.4  (1.4, 1.5) | 1.5  (1.5, 1.6) | 1.4  (1.4, 1.4) | 1.4  (1.4, 1.5) | 1.4  (1.4, 1.4) | 1.4  (1.4, 1.4) | 1.2  (1.2, 1.3) | 1.3  (1.3, 1.3) | 1.4  (1.3, 1.4) | 1.3  (1.3, 1.4) | 1.4  (1.4, 1.5) | 1.4  (1.4, 1.5) | 1.3  (1.2, 1.4) | 1.3  (1.2, 1.3) |
| Fractures | 1.4  (1.3, 1.4) | 1.5  (1.5, 1.5) | 1.4  (1.4, 1.4) | 1.5  (1.5, 1.5) | 1.3  (1.2, 1.3) | 1.4  (1.3, 1.4) | 1.3  (1.3, 1.4) | 1.5  (1.4, 1.5) | 1.3  (1.2, 1.3) | 1.4  (1.4, 1.5) | 1.9  (1.8, 2.0) | 1.9  (1.9, 2.0) | 1.2  (1.1, 1.2) | 1.2  (1.1, 1.3) | 1.3  (1.2, 1.4) | 1.3  (1.2, 1.4) |
| Urogenital System | 1.6  (1.6, 1.7) | 1.3  (1.3, 1.4) | 1.5  (1.5, 1.6) | 1.4  (1.3, 1.4) | 1.5  (1.4, 1.6) | 1.3  (1.2, 1.4) | 1.5  (1.5, 1.6) | 1.3  (1.3, 1.4) | 1.3  (1.3, 1.4) | 1.1  (1.1, 1.2) | 1.8  (1.6, 1.9) | 1.1  (1.0, 1.2) | 1.2  (1.1, 1.4) | 1.4  (1.2, 1.6) | 1.5  (1.4, 1.6) | 1.2  (1.1, 1.3) |
| Endocrine System | 1.3  (1.3, 1.4) | 1.4  (1.4, 1.4) | 1.3  (1.3, 1.3) | 1.4  (1.4, 1.4) | 1.4  (1.3, 1.4) | 1.3  (1.3, 1.4) | 1.4  (1.3, 1.4) | 1.4  (1.3, 1.4) | 1.1  (1.0, 1.1) | 1.1  (1.1, 1.2) | 1.3  (1.2, 1.3) | 1.3  (1.3, 1.4) | 1.3  (1.2, 1.4) | 1.3  (1.2, 1.4) | 1.8  (1.7, 1.9) | 1.6  (1.5, 1.7) |
| Circulatory System | 1.3  (1.2, 1.3) | 1.4  (1.4, 1.4) | 1.3  (1.3, 1.3) | 1.5  (1.4, 1.5) | 1.4  (1.4, 1.4) | 1.4  (1.4, 1.5) | 1.2  (1.2, 1.2) | 1.4  (1.3, 1.4) | 1.1  (1.1, 1.1) | 1.2  (1.2, 1.2) | 1.4  (1.4, 1.5) | 1.4  (1.3, 1.4) | 1.3  (1.2, 1.3) | 1.3  (1.2, 1.4) | 1.1  (1.1, 1.2) | 1.1  (1.1, 1.2) |
| Eye | 1.3  (1.3, 1.3) | 1.3  (1.3, 1.4) | 1.4  (1.3, 1.4) | 1.3  (1.3, 1.4) | 1.3  (1.3, 1.4) | 1.3  (1.2, 1.4) | 1.2  (1.2, 1.3) | 1.3  (1.2, 1.3) | 1.1  (1.1, 1.2) | 1.2  (1.1, 1.2) | 1.2  (1.1, 1.2) | 1.1  (1.0, 1.2) | 1.3  (1.2, 1.4) | 1.3  (1.2, 1.5) | 1.1  (1.0, 1.2) | 1.1  (1.0, 1.2) |
| Musculoskeletal System | 1.2  (1.1, 1.2) | 1.3  (1.3, 1.4) | 1.4  (1.3, 1.4) | 1.4  (1.3, 1.5) | 1.2  (1.2, 1.2) | 1.3  (1.2, 1.4) | 1.2  (1.1, 1.2) | 1.3  (1.2, 1.4) | 1.0  (0.9, 1.0) | 1.0  (1.0, 1.1) | 1.4  (1.3, 1.5) | 1.3  (1.2, 1.4) | 1.1  (1.0, 1.2) | 1.1  (1.0, 1.4) | 1  (0.9, 1.1) | 1.1  (0.9, 1.2) |
| Cancers | 1.1  (1.1, 1.1) | 1.2  (1.2, 1.2) | 1.2  (1.2, 1.3) | 1.2  (1.1, 1.2) | 1.2  (1.1, 1.2) | 1.2  (1.1, 1.2) | 1.1  (1.1, 1.1) | 1.2  (1.1, 1.2) | 1.0  (0.9, 1.0) | 1.0  (0.9, 1.0) | 1.1  (1.0, 1.2) | 1.2  (1.1, 1.2) | 1.1  (1.0, 1.2) | 1.1  (1.0, 1.3) | 1.1  (1.0, 1.1) | 1.1  (1.0, 1.2) |

**Table S5. Associations between any mental disorder and 16 physical conditions by age (odds ratios and 99.99% CI)**

|  | **Any Mental Health Disorder** | | **Sleep Disturbance** | | **Anxiety** | | **Depression** | | **Acute Stress Reaction** | | **Substance Abuse** | | **Phobia / Compulsive Disorder** | | **Psychosis** | |
| --- | --- | --- | --- | --- | --- | --- | --- | --- | --- | --- | --- | --- | --- | --- | --- | --- |
| **Physical Health Disorder** | **Younger** | **Older** | **Younger** | **Older** | **Younger** | **Older** | **Younger** | **Older** | **Younger** | **Older** | **Younger** | **Older** | **Younger** | **Older** | **Younger** | **Older** |
| Infections | 2.2  (2.2, 2.3) | 2.0  (2.0, 2.0) | 2.3  (2.2, 2.4) | 2.3  (2.3, 2.3) | 1.9  (1.8, 2.0) | 1.8  (1.8, 1.8) | 2.1  (2.0, 2.1) | 1.9  (1.9, 1.9) | 2.3  (2.2, 2.4) | 2.1  (2.1, 2.1) | 1.5  (1.4, 1.6) | 1.3  (1.3, 1.3) | 1.5  (1.4, 1.6) | 1.4  (1.4, 1.4) | 1.0  (1.0, 1.0) | 1.0  (0.9, 1.1) |
| Pain | 2.0  (2.0, 2.1) | 1.9  (1.9, 1.9) | 1.9  (1.8, 1.9) | 2.0  (2.0, 2.0) | 2.1  (2.1, 2.2) | 2.1  (2.1, 2.1) | 2.0  (1.9, 2.0) | 1.9  (1.9, 1.9) | 1.7  (1.7, 1.7) | 1.6  (1.6, 1.6) | 1.6  (1.6, 1.7) | 1.4  (1.4, 1.4) | 1.7  (1.6, 1.8) | 1.7  (1.7, 1.7) | 1.3  (1.3, 1.3) | 1.5  (1.4, 1.6) |
| Headaches | 2.0  (1.9, 2.0) | 2.0  (2.0, 2.0) | 1.8  (1.8, 1.8) | 1.9  (1.9, 1.9) | 1.7  (1.7, 1.8) | 1.8  (1.8, 1.8) | 1.8  (1.8, 1.9) | 1.8  (1.8, 1.8) | 1.6  (1.6, 1.7) | 1.7  (1.7, 1.7) | 1.3  (1.3, 1.4) | 1.3  (1.3, 1.3) | 1.5  (1.4, 1.6) | 1.6  (1.6, 1.6) | 1.3  (1.3, 1.3) | 1.3  (1.2, 1.3) |
| Gastrointestinal System | 2.0  (2.0, 2.1) | 1.8  (1.8, 1.8) | 1.8  (1.8, 1.9) | 1.8  (1.8, 1.8) | 2.1  (2.0, 2.2) | 1.9  (1.9, 1.9) | 1.8  (1.8, 1.9) | 1.6  (1.6, 1.6) | 1.5  (1.4, 1.5) | 1.3  (1.3, 1.3) | 2.6  (2.5, 2.8) | 2.2  (2.2, 2.2) | 1.7  (1.6, 1.8) | 1.6  (1.6, 1.6) | 1.4  (1.4, 1.4) | 1.9  (1.8, 2.1) |
| Pulmonary System & Allergy | 1.6  (1.6, 1.6) | 1.7  (1.7, 1.7) | 1.6  (1.5, 1.6) | 1.7  (1.7, 1.7) | 1.5  (1.5, 1.5) | 1.6  (1.6, 1.6) | 1.5  (1.5, 1.5) | 1.6  (1.6, 1.6) | 1.4  (1.4, 1.4) | 1.4  (1.4, 1.4) | 1.4  (1.3, 1.4) | 1.6  (1.6, 1.6) | 1.3  (1.3, 1.4) | 1.4  (1.4, 1.4) | 1.2  (1.2, 1.2) | 1.2  (1.2, 1.3) |
| Neurological System | 1.6  (1.6, 1.6) | 1.5  (1.5, 1.5) | 1.6  (1.5, 1.6) | 1.6  (1.6, 1.6) | 1.4  (1.4, 1.4) | 1.4  (1.4, 1.4) | 1.6  (1.5, 1.6) | 1.5  (1.5, 1.5) | 1.4  (1.4, 1.4) | 1.3  (1.3, 1.3) | 1.4  (1.3, 1.4) | 1.3  (1.3, 1.3) | 1.3  (1.2, 1.3) | 1.3  (1.3, 1.3) | 1.1  (1.1, 1.1) | 1.2  (1.1, 1.2) |
| Ear | 1.5  (1.5, 1.6) | 1.4  (1.4, 1.4) | 1.5  (1.4, 1.5) | 1.5  (1.5, 1.5) | 1.5  (1.5, 1.6) | 1.4  (1.4, 1.4) | 1.4  (1.4, 1.5) | 1.3  (1.3, 1.3) | 1.3  (1.3, 1.4) | 1.3  (1.3, 1.3) | 1.1  (1.0, 1.1) | 0.9  (0.9, 0.9) | 1.4  (1.4, 1.5) | 1.3  (1.3, 1.3) | 0.9  (0.9, 0.9) | 1.2  (1.1, 1.2) |
| Hematological System | 1.4  (1.4, 1.5) | 1.5  (1.5, 1.5) | 1.3  (1.3, 1.4) | 1.5  (1.5, 1.5) | 1.4  (1.4, 1.5) | 1.6  (1.6, 1.6) | 1.4  (1.4, 1.5) | 1.5  (1.5, 1.5) | 1.1  (1.1, 1.2) | 1.1  (1.1, 1.1) | 1.2  (1.1, 1.3) | 1.6  (1.6, 1.6) | 1.3  (1.3, 1.4) | 1.5  (1.5, 1.5) | 1.7  (1.7, 1.7) | 1.4  (1.3, 1.5) |
| Skin | 1.5  (1.4, 1.5) | 1.4  (1.4, 1.4) | 1.4  (1.4, 1.5) | 1.5  (1.5, 1.5) | 1.4  (1.4, 1.5) | 1.4  (1.4, 1.4) | 1.4  (1.4, 1.4) | 1.4  (1.4, 1.4) | 1.3  (1.3, 1.3) | 1.2  (1.2, 1.2) | 1.4  (1.3, 1.4) | 1.3  (1.3, 1.3) | 1.5  (1.4, 1.6) | 1.4  (1.4, 1.4) | 1.2  (1.2, 1.2) | 1.4  (1.3, 1.4) |
| Fractures | 1.5  (1.5, 1.5) | 1.4  (1.4, 1.4) | 1.5  (1.4, 1.5) | 1.4  (1.4, 1.4) | 1.3  (1.3, 1.4) | 1.3  (1.3, 1.3) | 1.4  (1.4, 1.5) | 1.4  (1.4, 1.4) | 1.4  (1.4, 1.5) | 1.3  (1.3, 1.3) | 1.9  (1.8, 2.0) | 1.9  (1.9, 1.9) | 1.2  (1.1, 1.3) | 1.2  (1.2, 1.2) | 1.2  (1.2, 1.2) | 1.3  (1.2, 1.4) |
| Urogenital System | 1.7  (1.6, 1.8) | 1.3  (1.3, 1.3) | 1.6  (1.5, 1.6) | 1.4  (1.4, 1.4) | 1.5  (1.4, 1.6) | 1.2  (1.2, 1.2) | 1.6  (1.6, 1.7) | 1.3  (1.3, 1.3) | 1.4  (1.4, 1.5) | 1.1  (1.1, 1.1) | 1.8  (1.7, 2.0) | 1.1  (1.1, 1.1) | 1.3  (1.2, 1.5) | 1.1  (1.1, 1.1) | 1.1  (1.1, 1.1) | 1.6  (1.4, 1.8) |
| Endocrine System | 1.5  (1.4, 1.5) | 1.3  (1.3, 1.3) | 1.4  (1.4, 1.5) | 1.3  (1.3, 1.3) | 1.4  (1.3, 1.4) | 1.3  (1.3, 1.3) | 1.5  (1.4, 1.5) | 1.3  (1.3, 1.3) | 1.2  (1.1, 1.2) | 1.0  (1.0, 1.0) | 1.3  (1.2, 1.4) | 1.2  (1.2, 1.2) | 1.3  (1.2, 1.4) | 1.2  (1.2, 1.2) | 1.6  (1.6, 1.6) | 1.8  (1.7, 1.9) |
| Circulatory System | 1.4  (1.4, 1.5) | 1.3  (1.3, 1.3) | 1.5  (1.5, 1.6) | 1.4  (1.4, 1.4) | 1.4  (1.4, 1.5) | 1.4  (1.4, 1.4) | 1.4  (1.3, 1.4) | 1.2  (1.2, 1.2) | 1.2  (1.2, 1.3) | 1.0  (1.0, 1.0) | 1.3  (1.3, 1.4) | 1.3  (1.3, 1.3) | 1.2  (1.1, 1.3) | 1.2  (1.2, 1.2) | 1.0  (1.0, 1.0) | 1.2  (1.1, 1.3) |
| Eye | 1.3  (1.3, 1.4) | 1.3  (1.3, 1.3) | 1.3  (1.3, 1.4) | 1.4  (1.4, 1.4) | 1.3  (1.2, 1.3) | 1.3  (1.3, 1.3) | 1.3  (1.2, 1.3) | 1.2  (1.2, 1.2) | 1.2  (1.2, 1.2) | 1.1  (1.1, 1.1) | 1.1  (1.0, 1.2) | 1.1  (1.1, 1.1) | 1.2  (1.1, 1.3) | 1.2  (1.2, 1.2) | 1.0  (1.0, 1.0) | 1.1  (1.0, 1.2) |
| Musculoskeletal System | 1.3  (1.2, 1.3) | 1.2  (1.2, 1.2) | 1.4  (1.3, 1.4) | 1.4  (1.4, 1.4) | 1.2  (1.1, 1.2) | 1.2  (1.2, 1.2) | 1.3  (1.2, 1.3) | 1.1  (1.1, 1.1) | 1.0  (1.0, 1.1) | 0.9  (0.9, 0.9) | 1.3  (1.2, 1.5) | 1.3  (1.3, 1.3) | 1.0  (0.9, 1.2) | 1.0  (1.0, 1.0) | 1.0  (1.0, 1.0) | 1.1  (0.9, 1.2) |
| Cancers | 1.2  (1.1, 1.2) | 1.1  (1.1, 1.1) | 1.2  (1.2, 1.3) | 1.2  (1.2, 1.2) | 1.1  (1.0, 1.2) | 1.1  (1.1, 1.1) | 1.1  (1.1, 1.2) | 1.1  (1.1, 1.1) | 1.1  (1.0, 1.1) | 0.9  (0.9, 0.9) | 1.0  (0.9, 1.1) | 1.0  (1.0, 1.0) | 1.0  (0.8, 1.1) | 0.9  (0.9, 0.9) | 1.0  (1.0, 1.0) | 1.0  (0.9, 1.1) |

**Table S6. Associations between any mental disorder and 16 physical conditions by education (odds ratios and 99.99% CI)**

|  | **Any Mental Health Disorder** | | **Sleep Disturbance** | | **Anxiety** | | **Depression** | | **Acute Stress Reaction** | | **Substance Abuse** | | **Phobia / Compulsive Disorder** | | **Psychosis** | |
| --- | --- | --- | --- | --- | --- | --- | --- | --- | --- | --- | --- | --- | --- | --- | --- | --- |
| **Physical Health Disorder** | **No College** | **College** | **No College** | **College** | **No College** | **College** | **No College** | **College** | **No College** | **College** | **No College** | **College** | **No College** | **College** | **No College** | **College** |
| Infections | 2.0  (2.0, 2.1) | 2.2  (2.1, 2.3) | 2.3  (2.2, 2.4) | 2.3  (2.2, 2.4) | 1.7  (1.7, 1.8) | 2.0  (1.9, 2.1) | 1.9  (1.9, 2.0) | 2.0  (2.0, 2.1) | 2.1  (2.1, 2.2) | 2.2  (2.1, 2.3) | 1.3  (1.3, 1.4) | 1.4  (1.3, 1.6) | 1.4  (1.3, 1.4) | 1.6  (1.4, 1.8) | 1.0  (0.9, 1.0) | 1.1  (1.0, 1.2) |
| Pain | 2.0  (2.0, 2.0) | 2.0  (2.0, 2.0) | 2.0  (2.0, 2.1) | 1.9  (1.9, 2.0) | 2.1  (2.1, 2.2) | 2.2  (2.1, 2.3) | 1.9  (1.9, 2.0) | 1.9  (1.8, 1.9) | 1.6  (1.6, 1.6) | 1.7  (1.6, 1.7) | 1.6  (1.5, 1.6) | 1.5  (1.4, 1.6) | 1.7  (1.6, 1.8) | 1.8  (1.7, 2.0) | 1.4  (1.3, 1.4) | 1.5  (1.4, 1.6) |
| Headaches | 2.0  (1.9, 2.0) | 2.0  (1.9, 2.0) | 1.9  (1.9, 1.9) | 1.8  (1.8, 1.9) | 1.8  (1.7, 1.8) | 1.8  (1.8, 1.9) | 1.8  (1.8, 1.9) | 1.8  (1.7, 1.8) | 1.6  (1.6, 1.6) | 1.6  (1.6, 1.7) | 1.3  (1.3, 1.4) | 1.3  (1.2, 1.4) | 1.5  (1.5, 1.6) | 1.5  (1.4, 1.7) | 1.3  (1.2, 1.3) | 1.3  (1.2, 1.4) |
| Gastrointestinal System | 1.9  (1.9, 2.0) | 1.8  (1.8, 1.9) | 1.8  (1.8, 1.9) | 1.7  (1.6, 1.8) | 2.1  (2.0, 2.1) | 1.9  (1.8, 2.0) | 1.7  (1.7, 1.8) | 1.8  (1.7, 1.8) | 1.4  (1.3, 1.4) | 1.5  (1.4, 1.6) | 2.6  (2.5, 2.7) | 2.3  (2.1, 2.5) | 1.7  (1.6, 1.8) | 1.7  (1.5, 1.9) | 1.8  (1.7, 1.8) | 1.6  (1.4, 1.8) |
| Pulmonary System & Allergy | 1.7  (1.6, 1.7) | 1.6  (1.6, 1.6) | 1.7  (1.7, 1.7) | 1.6  (1.6, 1.6) | 1.6  (1.6, 1.6) | 1.5  (1.5, 1.6) | 1.6  (1.5, 1.6) | 1.5  (1.5, 1.6) | 1.4  (1.4, 1.4) | 1.5  (1.4, 1.5) | 1.5  (1.5, 1.6) | 1.5  (1.4, 1.6) | 1.4  (1.3, 1.4) | 1.4  (1.3, 1.5) | 1.2  (1.2, 1.3) | 1.2  (1.1, 1.3) |
| Neurological System | 1.6  (1.5, 1.6) | 1.6  (1.6, 1.7) | 1.6  (1.6, 1.6) | 1.6  (1.6, 1.7) | 1.4  (1.4, 1.4) | 1.5  (1.4, 1.6) | 1.5  (1.5, 1.5) | 1.6  (1.6, 1.7) | 1.3  (1.3, 1.4) | 1.4  (1.4, 1.5) | 1.4  (1.3, 1.4) | 1.5  (1.4, 1.6) | 1.3  (1.2, 1.4) | 1.4  (1.3, 1.5) | 1.1  (1.0, 1.1) | 1.3  (1.2, 1.4) |
| Ear | 1.4  (1.4, 1.4) | 1.6  (1.5, 1.6) | 1.4  (1.4, 1.5) | 1.6  (1.5, 1.6) | 1.4  (1.4, 1.4) | 1.6  (1.5, 1.6) | 1.3  (1.3, 1.4) | 1.5  (1.4, 1.5) | 1.3  (1.2, 1.3) | 1.4  (1.4, 1.4) | 0.9  (0.9, 1.0) | 1.1  (1.0, 1.2) | 1.3  (1.2, 1.4) | 1.5  (1.4, 1.7) | 0.9  (0.9, 1.0) | 1.2  (1.1, 1.3) |
| Hematological System | 1.5  (1.5, 1.6) | 1.4  (1.4, 1.5) | 1.5  (1.4, 1.5) | 1.4  (1.3, 1.4) | 1.6  (1.5, 1.6) | 1.4  (1.4, 1.5) | 1.5  (1.5, 1.5) | 1.4  (1.4, 1.5) | 1.1  (1.0, 1.1) | 1.2  (1.1, 1.2) | 1.5  (1.4, 1.6) | 1.3  (1.2, 1.5) | 1.5  (1.4, 1.6) | 1.4  (1.2, 1.6) | 1.6  (1.5, 1.7) | 1.5  (1.3, 1.6) |
| Skin | 1.4  (1.4, 1.5) | 1.5  (1.4, 1.5) | 1.5  (1.5, 1.5) | 1.5  (1.4, 1.5) | 1.4  (1.4, 1.4) | 1.5  (1.4, 1.5) | 1.4  (1.4, 1.4) | 1.4  (1.4, 1.4) | 1.2  (1.2, 1.3) | 1.3  (1.3, 1.3) | 1.3  (1.3, 1.3) | 1.5  (1.4, 1.6) | 1.4  (1.4, 1.5) | 1.6  (1.4, 1.7) | 1.3  (1.2, 1.3) | 1.4  (1.3, 1.4) |
| Fractures | 1.4  (1.4, 1.5) | 1.5  (1.4, 1.5) | 1.5  (1.4, 1.5) | 1.4  (1.4, 1.5) | 1.4  (1.3, 1.4) | 1.3  (1.2, 1.4) | 1.4  (1.4, 1.4) | 1.4  (1.4, 1.5) | 1.3  (1.3, 1.4) | 1.4  (1.4, 1.5) | 1.9  (1.8, 1.9) | 2.0  (1.9, 2.2) | 1.2  (1.2, 1.3) | 1.2  (1.1, 1.4) | 1.2  (1.2, 1.3) | 1.5  (1.4, 1.6) |
| Urogenital System | 1.4  (1.3, 1.4) | 1.4  (1.4, 1.5) | 1.4  (1.4, 1.4) | 1.4  (1.4, 1.5) | 1.3  (1.3, 1.4) | 1.3  (1.2, 1.4) | 1.4  (1.3, 1.4) | 1.4  (1.3, 1.5) | 1.2  (1.1, 1.2) | 1.3  (1.2, 1.4) | 1.2  (1.2, 1.3) | 1.5  (1.3, 1.7) | 1.2  (1.1, 1.4) | 1.3  (1.0, 1.6) | 1.2  (1.1, 1.3) | 1.4  (1.2, 1.6) |
| Endocrine System | 1.3  (1.3, 1.3) | 1.5  (1.5, 1.6) | 1.3  (1.3, 1.4) | 1.4  (1.4, 1.5) | 1.3  (1.3, 1.3) | 1.4  (1.3, 1.4) | 1.3  (1.3, 1.3) | 1.6  (1.5, 1.6) | 1.0  (1.0, 1.1) | 1.3  (1.3, 1.4) | 1.1  (1.1, 1.2) | 1.6  (1.5, 1.7) | 1.3  (1.2, 1.3) | 1.4  (1.2, 1.5) | 1.5  (1.5, 1.6) | 2.2  (2.0, 2.4) |
| Circulatory System | 1.3  (1.2, 1.3) | 1.5  (1.4, 1.5) | 1.3  (1.3, 1.4) | 1.4  (1.4, 1.5) | 1.3  (1.3, 1.4) | 1.4  (1.4, 1.5) | 1.2  (1.2, 1.2) | 1.4  (1.4, 1.5) | 1.1  (1.0, 1.1) | 1.3  (1.3, 1.4) | 1.1  (1.1, 1.1) | 1.7  (1.6, 1.8) | 1.2  (1.1, 1.2) | 1.4  (1.2, 1.5) | 1.0  (0.9, 1.0) | 1.4  (1.2, 1.4) |
| Eye | 1.2  (1.2, 1.3) | 1.4  (1.4, 1.4) | 1.3  (1.3, 1.3) | 1.4  (1.3, 1.4) | 1.2  (1.2, 1.3) | 1.4  (1.3, 1.4) | 1.2  (1.2, 1.2) | 1.4  (1.3, 1.4) | 1.1  (1.1, 1.1) | 1.3  (1.2, 1.4) | 1.0  (1.0, 1.1) | 1.3  (1.1, 1.4) | 1.2  (1.2, 1.3) | 1.4  (1.2, 1.6) | 1.0  (1.0, 1.1) | 1.3  (1.1, 1.4) |
| Musculoskeletal System | 1.2  (1.1, 1.2) | 1.4  (1.3, 1.5) | 1.3  (1.3, 1.4) | 1.5  (1.4, 1.5) | 1.2  (1.2, 1.2) | 1.3  (1.2, 1.4) | 1.1  (1.1, 1.2) | 1.4  (1.3, 1.5) | 0.9  (0.9, 1.0) | 1.2  (1.1, 1.3) | 1.2  (1.2, 1.3) | 1.5  (1.4, 1.8) | 1.1  (1.0, 1.2) | 1.2  (1.0, 1.5) | 0.9  (0.8, 1.0) | 1.4  (1.1, 1.6) |
| Cancers | 1.1  (1.0, 1.1) | 1.2  (1.2, 1.3) | 1.1  (1.1, 1.2) | 1.3  (1.2, 1.3) | 1.1  (1.1, 1.1) | 1.1  (1.1, 1.2) | 1.1  (1.0, 1.1) | 1.2  (1.2, 1.3) | 0.9  (0.9, 0.9) | 1.1  (1.1, 1.2) | 1.0  (1.0, 1.0) | 1.2  (1.1, 1.3) | 1.0  (0.9, 1.1) | 1.1  (1.0, 1.3) | 0.9  (0.8, 0.9) | 1.2  (1.1, 1.4) |

**Table S7. Multimorbidity: Number of different mental health disorders as a function of the number of different physical health conditions**

|  | Number of Physical Health Conditions | | | | | | | | | | |
| --- | --- | --- | --- | --- | --- | --- | --- | --- | --- | --- | --- |
| Number of Mental Health Disorders | 0 | 1 | 2 | 3 | 4 | 5 | 6 | 7 | 8 | 9 | 10+ |
| 0 | 55,493 | 124,378 | 190,616 | 213,978 | 191,898 | 143,824 | 90,880 | 50,684 | 24,731 | 10,375 | 5,281 |
|  | (80.2%) | (70.6%) | (63.1%) | (55.8%) | (49%) | (43.1%) | (37.5%) | (33.0%) | (29.1%) | (25.7%) | (20.8%) |
| 1 | 9,282 | 33,177 | 67,582 | 95,747 | 104,656 | 92,378 | 68,342 | 42,912 | 22,924 | 10,634 | 6,182 |
|  | (13.4%) | (18.8%) | (22.4%) | (25.0%) | (26.8%) | (27.7%) | (28.2%) | (27.9%) | (26.9%) | (26.4%) | (24.3%) |
| 2 | 3,009 | 12,209 | 27,777 | 45,079 | 55,348 | 54,318 | 44,336 | 30,242 | 17,782 | 8,604 | 5,480 |
|  | (4.3%) | (6.9%) | (9.2%) | (11.8%) | (14.2%) | (16.3%) | (8.3%) | (19.7%) | (20.9%) | (21.3%) | (21.5%) |
| 3 | 1,020 | 4,530 | 11,081 | 19,447 | 25,640 | 27,543 | 24,324 | 17,914 | 11,257 | 5,886 | 4,187 |
|  | (1.5%) | (2.6%) | (3.7%) | (5.1%) | (6.5%) | (8.2%) | (10.0%) | (11.6%) | (13.2%) | (14.6%) | (16.5%) |
| 4 | 426 | 1,932 | 4,984 | 9,419 | 13,727 | 15,793 | 14,749 | 11,963 | 8,444 | 4,840 | 4,309 |
|  | (0.6%) | (1.1%) | (1.6%) | (2.4%) | (3.5%) | (4.7%) | (6.1%) | (7.8%) | (9.9%) | (12.0%) | (16.9%) |

**Table S8. Multimorbidity: Number of different physical health conditions as a function of the number of different mental health disorders.**

|  | Number of Mental Health Disorders | | | | |
| --- | --- | --- | --- | --- | --- |
| Number of Physical Health Conditions | 0 | 1 | 2 | 3 | 4+ |
| 0 | 55,493 (5.0%) | 9,282 (1.7%) | 3,009 (1.0%) | 1,020 (0.7%) | 426 (0.5%) |
| 1 | 124,378 (11.3%) | 33,177 (6.0%) | 12,209 (4.0%) | 4,530 (3.0%) | 1,932 (2.1%) |
| 2 | 190,616 (17.3%) | 67,582 (12.2%) | 27,777 (9.1%) | 11,081 (7.2%) | 4,984 (5.5%) |
| 3 | 213,978 (19.4%) | 95,747 (17.3%) | 45,079 (14.8%) | 19,447 (12.7%) | 9,419 (10.4%) |
| 4 | 191,898 (17.4%) | 104,656 (18.9%) | 55,348 (18.2%) | 25,640 (16.8%) | 13,727 (15.2%) |
| 5 | 143,824 (13.1%) | 92,378 (16.7%) | 54,318 (17.9%) | 27,543 (18.0%) | 15,793 (17.4%) |
| 6 | 90,880 (8.2%) | 68,342 (12.3%) | 44,336 (14.6%) | 24,324 (15.9%) | 14,749 (16.3%) |
| 7 | 50,684 (4.6%) | 42,912 (7.8%) | 30,242 (9.9%) | 17,914 (11.7%) | 11,963 (13.2%) |
| 8 | 24,731 (2.2%) | 22,924 (4.1%) | 17,782 (5.8%) | 11,257 (7.4%) | 8,444 (9.3%) |
| 9 | 10,375 (0.9%) | 10,634 (1.9%) | 8,604 (2.8%) | 5,886 (3.8%) | 4,840 (5.3%) |
| 10 | 5,281 (0.5%) | 6,182 (1.1%) | 5,480 (1.8%) | 4,187 (2.7%) | 4,309 (4.8%) |

**Table S9. Time from first observed diagnosis of any mental disorder to first subsequent diagnosis of physical health conditions.**

| Physical Health Condition | Within 1 month | 1-6 months | 6-12 months | 1-2 years | 2+ years |
| --- | --- | --- | --- | --- | --- |
| Infections | 55,130 (6.4%) | 173,364 (20.2%) | 127,351 (14.8%) | 164,884 (19.2%) | 338,411 (39.4%) |
| Pain | 38,443 (6.6%) | 71,185 (12.2%) | 55,466 (9.5%) | 87,250 (15.0%) | 330,589 (56.7%) |
| Headaches | 10,389 (5.2%) | 17,563 (8.8%) | 15,003 (7.5%) | 25,292 (12.7%) | 131,201 (65.8%) |
| Gastrointestinal System | 6,417 (5.6%) | 11,177 (9.7%) | 7,838 (6.8%) | 13,140 (11.4%) | 76,922 (66.6%) |
| Pulmonary System and Allergy | 25,767 (5.2%) | 65,918 (13.4%) | 50,231 (10.2%) | 74,356 (15.1%) | 276,306 (56.1%) |
| Neurological System | 23,965 (7.3%) | 37,841 (11.6%) | 27,597 (8.4%) | 42,303 (12.9%) | 195,719 (59.8%) |
| Ear | 6,135 (3.2%) | 12,663 (6.5%) | 11,231 (5.8%) | 20,916 (10.8%) | 142,552 (73.7%) |
| Hematological System | 4,178 (4.6%) | 8,390 (9.3%) | 5,950 (6.6%) | 9,721 (10.8%) | 61,948 (68.7%) |
| Skin | 9,840 (3.1%) | 24,700 (7.9%) | 22,265 (7.1%) | 38,069 (12.1%) | 219,311 (69.8%) |
| Fractures | 4,279 (2.6%) | 11,170 (6.7%) | 10,200 (6.1%) | 18,851 (11.3%) | 122,012 (73.3%) |
| Urogenital System | 1,074 (2.3%) | 2,968 (6.4%) | 2,546 (5.5%) | 4,794 (10.3%) | 35,282 (75.6%) |
| Endocrine System | 24,685 (12.7%) | 32,845 (16.9%) | 16,511 (8.5%) | 21,080 (10.9%) | 99,016 (51.0%) |
| Circulatory System | 47,512 (12.4%) | 59,739 (15.5%) | 33,643 (8.8%) | 44,491 (11.6%) | 198,975 (51.8%) |
| Eye | 1,704 (2.2%) | 5,411 (7.1%) | 4,737 (6.2%) | 7,876 (10.3%) | 56,361 (74.1%) |
| Musculoskeletal System | 6,641 (10.1%) | 7,389 (11.3%) | 4,433 (6.8%) | 6,271 (9.6%) | 40,752 (62.2%) |
| Cancers | 9,046 (9.3%) | 9,051 (9.3%) | 5,312 (5.4%) | 8,335 (8.6%) | 65,731 (67.4%) |

**Table S10. Time from first observed diagnosis of a physical health condition to first subsequent diagnosis of a mental health disorder**

| Physical Health Condition | Within 1 month | 1-6 months | 6-12 months | 1-2 years | 2+ years |
| --- | --- | --- | --- | --- | --- |
| Infections | 74,734 (8.4%) | 134,828 (15.1%) | 81,759 (9.2%) | 116,336 (13.0%) | 486,048 (54.4%) |
| Pain | 72,605 (12.4%) | 107,778 (18.4%) | 60,162 (10.3%) | 81,131 (13.8%) | 264,615 (45.1%) |
| Headaches | 28,731 (13.4%) | 43,604 (20.3%) | 23,205 (10.8%) | 30,690 (14.3%) | 88,508 (41.2%) |
| Gastrointestinal System | 20,379 (18.1%) | 24,516 (21.7%) | 12,217 (10.8%) | 15,414 (13.7%) | 40,327 (35.7%) |
| Pulmonary System and Allergy | 54,068 (10.9%) | 88,768 (17.9%) | 50,819 (10.2%) | 69,936 (14.1%) | 233,081 (46.9%) |
| Neurological System | 37,194 (11.5%) | 58,566 (18.2%) | 34,441 (10.7%) | 46,193 (14.3%) | 146,055 (45.3%) |
| Ear | 23,643 (12.9%) | 38,281 (20.9%) | 20,992 (11.5%) | 26,915 (14.7%) | 73,298 (40.0%) |
| Hematological System | 15,293 (16.9%) | 17,587 (19.5%) | 9,418 (10.4%) | 12,399 (13.7%) | 35,650 (39.5%) |
| Skin | 38,178 (12.6%) | 58,300 (19.2%) | 32,924 (10.8%) | 43,944 (14.4%) | 130,775 (43.0%) |
| Fractures | 20,940 (12.8%) | 33,860 (20.7%) | 18,458 (11.3%) | 24,033 (14.7%) | 66,319 (40.5%) |
| Urogenital System | 5,739 (13.7%) | 9,384 (22.4%) | 4,857 (11.6%) | 6,195 (14.8%) | 15,641 (37.4%) |
| Endocrine System | 26,592 (15.8%) | 30,439 (18.0%) | 17,271 (10.2%) | 22,672 (13.4%) | 71,806 (42.5%) |
| Circulatory System | 51,910 (15.3%) | 61,973 (18.3%) | 34,246 (10.1%) | 45,356 (13.4%) | 144,622 (42.8%) |
| Eye | 9,827 (12.6%) | 15,884 (20.4%) | 8,622 (11.1%) | 11,318 (14.5%) | 32,210 (41.4%) |
| Musculoskeletal System | 6,501 (12.0%) | 10,699 (19.7%) | 6,202 (11.4%) | 7,800 (14.4%) | 23,071 (42.5%) |
| Cancers | 11,522 (16.2%) | 16,413 (23.0%) | 8,663 (12.2%) | 10,364 (14.6%) | 24,283 (34.1%) |

**Figure S1. Associations between sleep disturbance and 16 physical-health conditions. Panel A** shows associations (odds ratios and 99.99% confidence intervals) between sleep disturbance and 16 physical-health conditions in primary-care practices across 14 years. **Panel B** shows the risk of 16 physical-health conditions among patients who did and did not present with sleep disturbance. Inverse probability weighting was used to balance on four demographic variables: age at baseline, sex assigned at birth, educational attainment, and county of residence.

| **A. Associations between sleep disturbance and 16 physical-health conditions** | **B. Risk of 16 physical-health conditions for patients with and without sleep disturbance** |
| --- | --- |
| 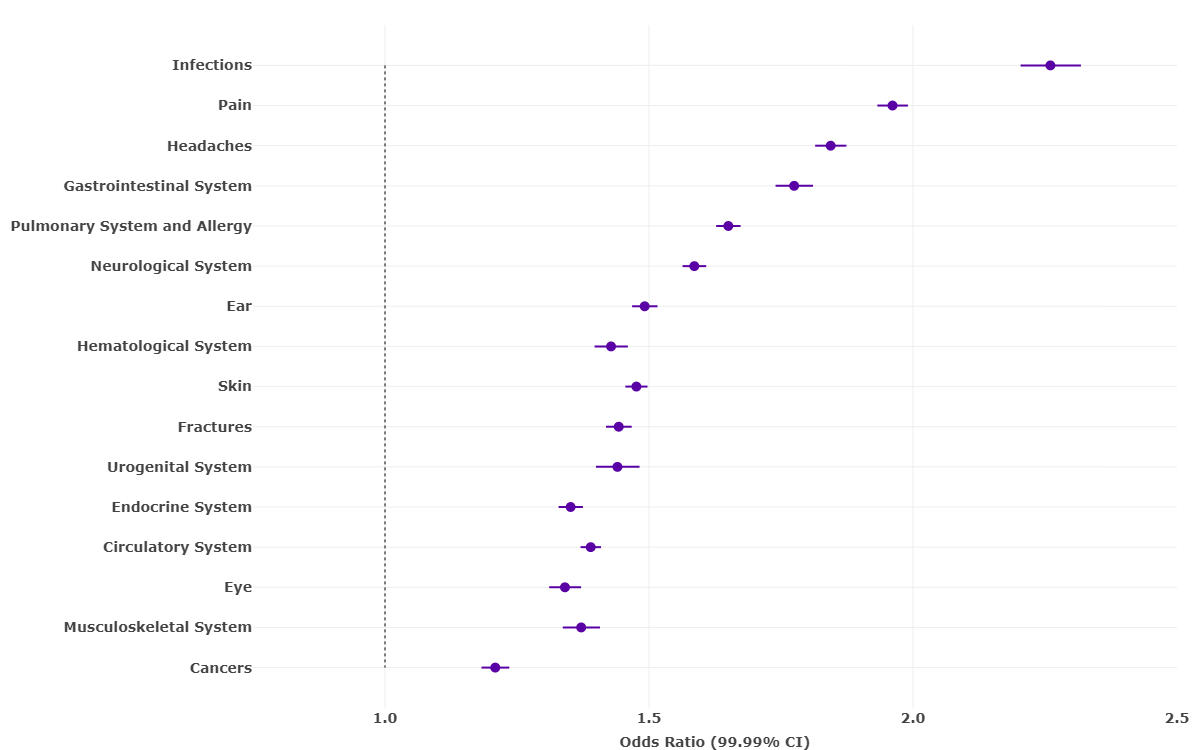 | 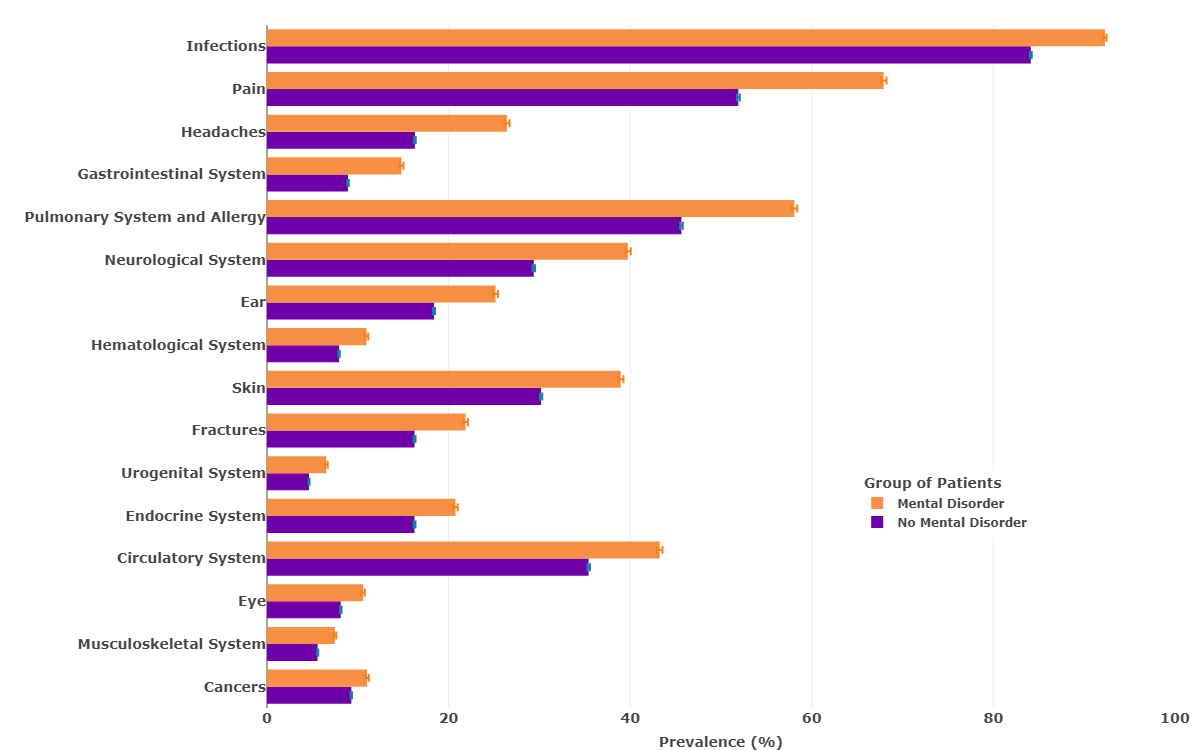 |

**Figure S2. Associations between anxiety and 16 physical-health conditions**. **Panel A** shows associations (odds ratios and 99.99% confidence intervals) between anxiety and 16 physical-health conditions in primary-care practices across 14 years. **Panel B** shows the risk of 16 physical-health conditions among patients who did and did not present with anxiety. Inverse probability weighting was used to balance on four demographic variables: age at baseline, sex assigned at birth, educational attainment, and county of residence.

| **A. Associations between anxiety and 16 physical-health conditions** | **B. Risk of 16 physical-health conditions for patients with and without anxiety** |
| --- | --- |
| 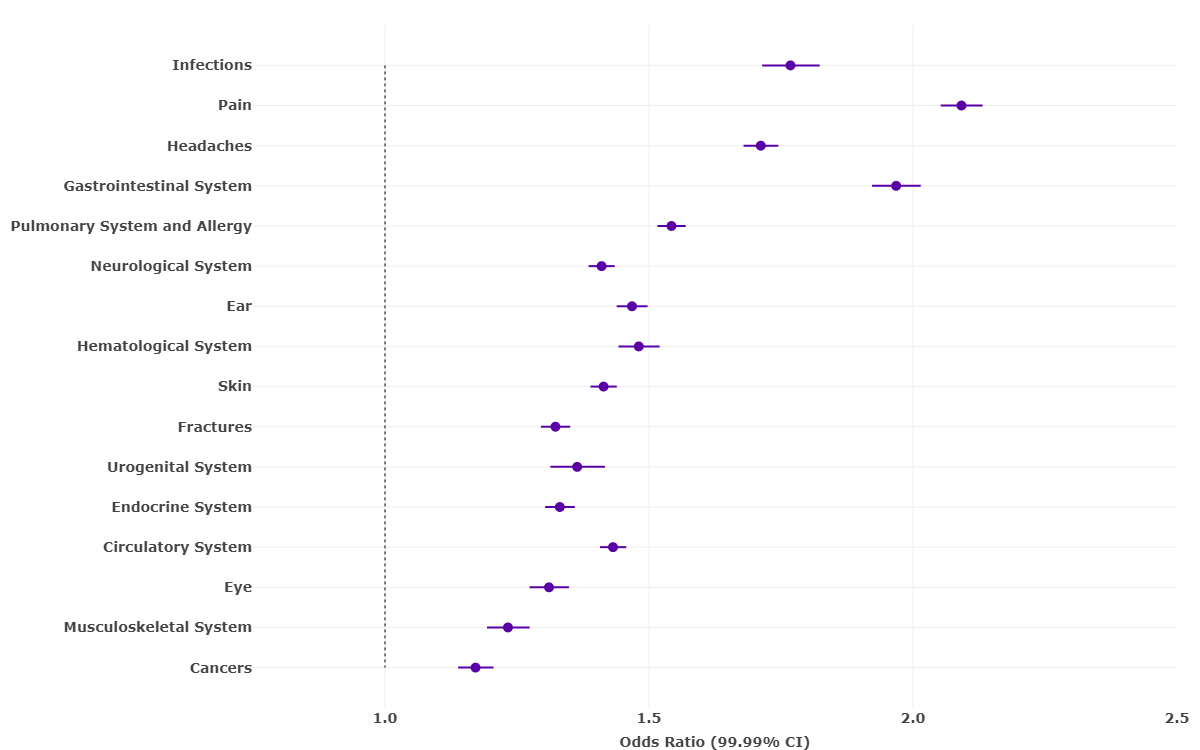 | 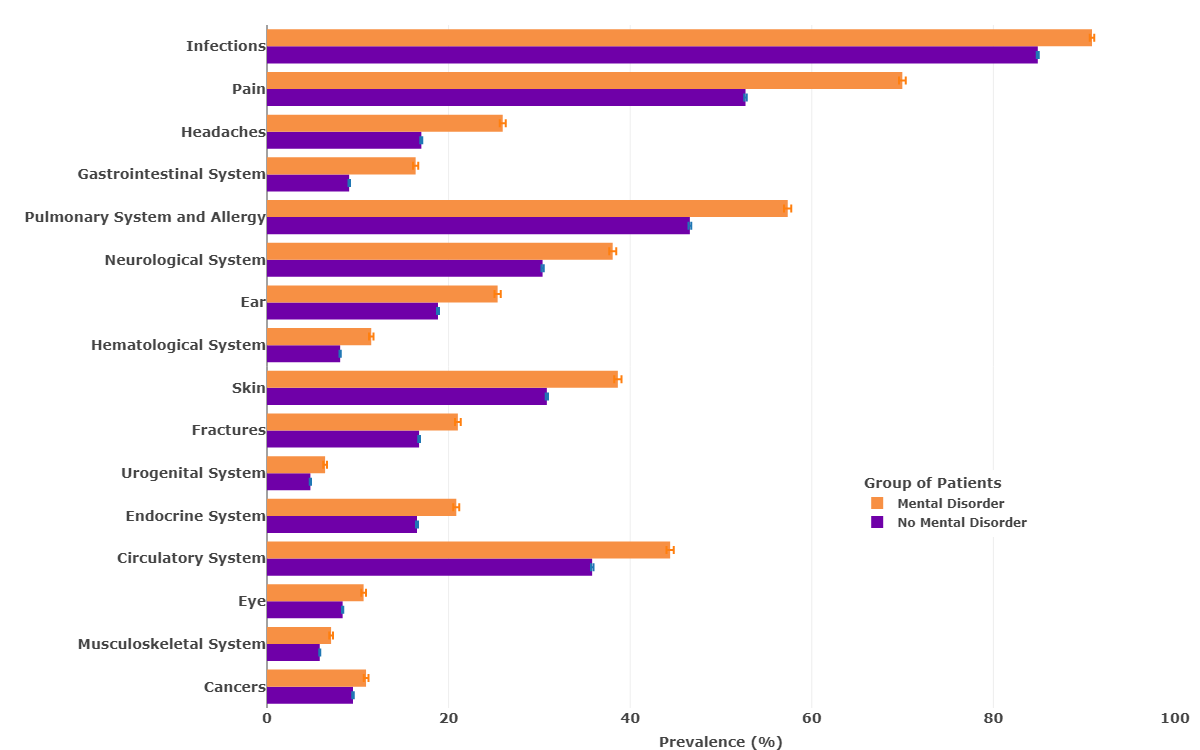 |

**Figure S3. Associations between depression and 16 physical-health conditions. Panel A** shows associations (odds ratios and 99.99% confidence intervals) between depression and 16 physical-health conditions in primary-care practices across 14 years. **Panel B** shows the risk of 16 physical-health conditions among patients who did and did not present with depression. Inverse probability weighting was used to balance on four demographic variables: age at baseline, sex assigned at birth, educational attainment, and county of residence.

| **A. Associations between depression and 16 physical-health conditions** | **B. Risk of 16 physical-health conditions for patients with and without depression** |
| --- | --- |
| 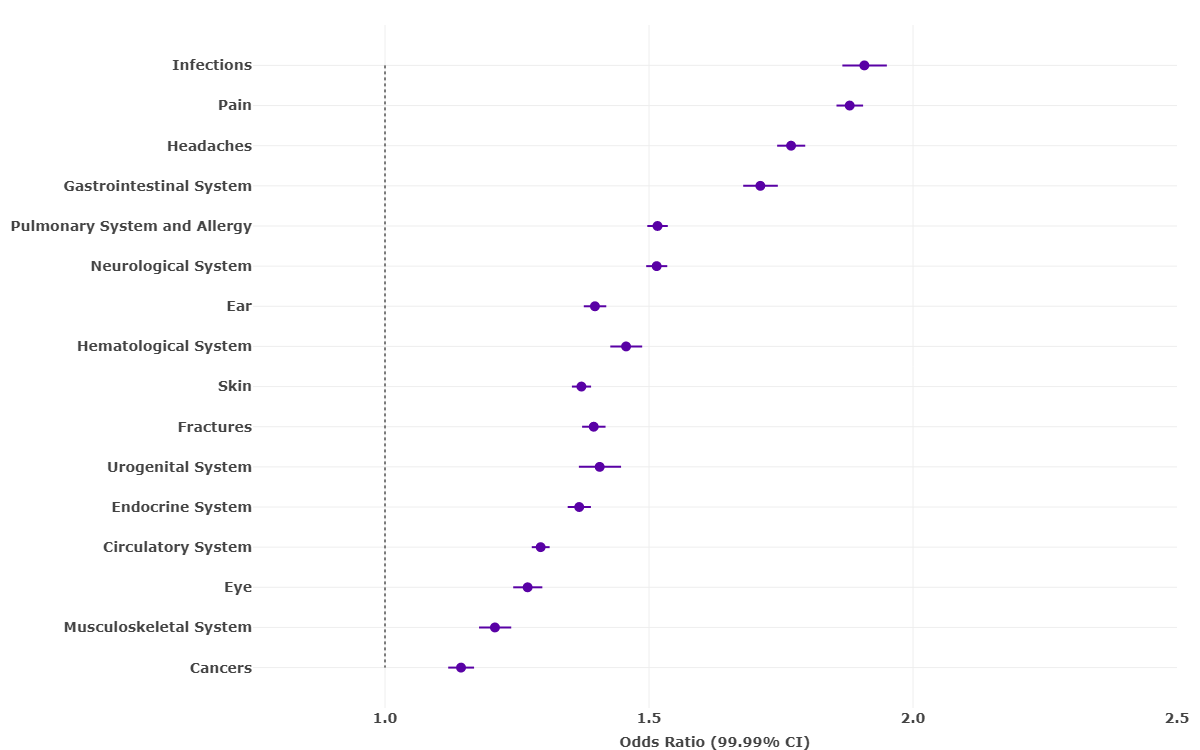 | 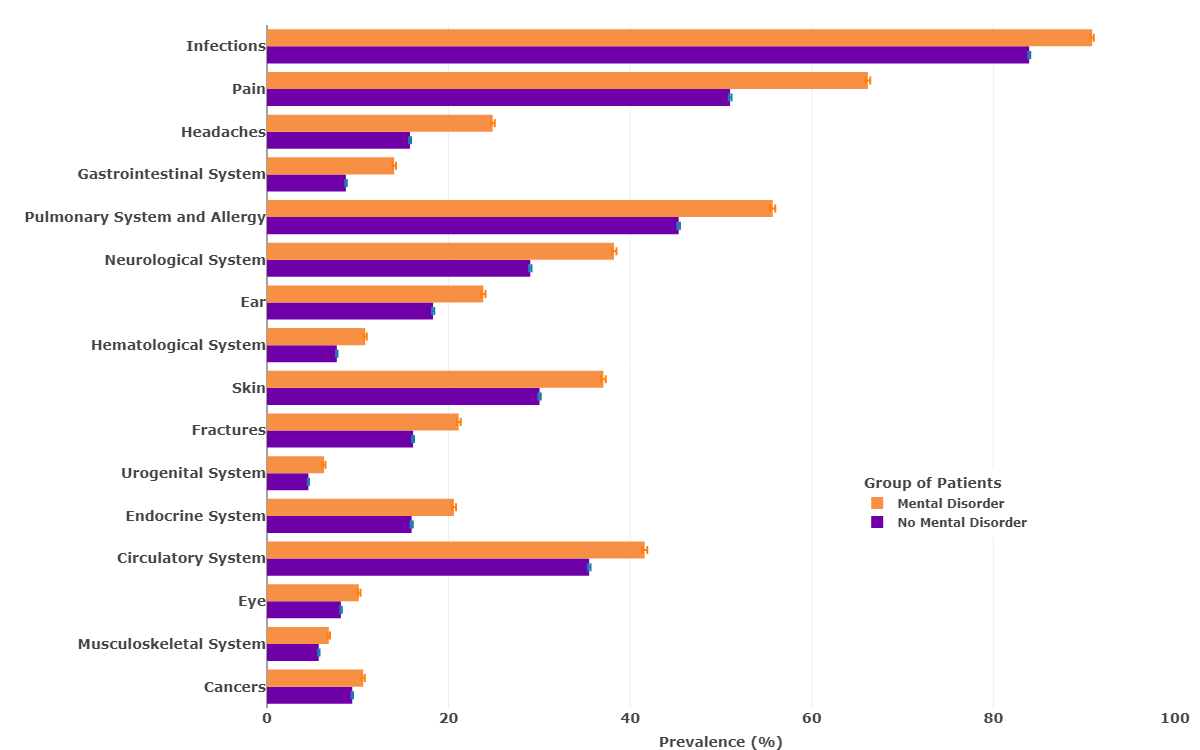 |

**Figure S4. Associations between acute stress reaction and 16 physical-health conditions. Panel A** shows associations (odds ratios and 99.99% confidence intervals) between acute stress reaction and 16 physical-health conditions in primary-care practices across 14 years. **Panel B** shows the risk of 16 physical-health conditions among patients who did and did not present with acute stress reaction. Inverse probability weighting was used to balance on four demographic variables: age at baseline, sex assigned at birth, educational attainment, and county of residence.

| **A. Associations between acute stress reaction and 16 physical-health conditions** | **B. Risk of 16 physical-health conditions for patients with and without acute stress reaction** |
| --- | --- |
| 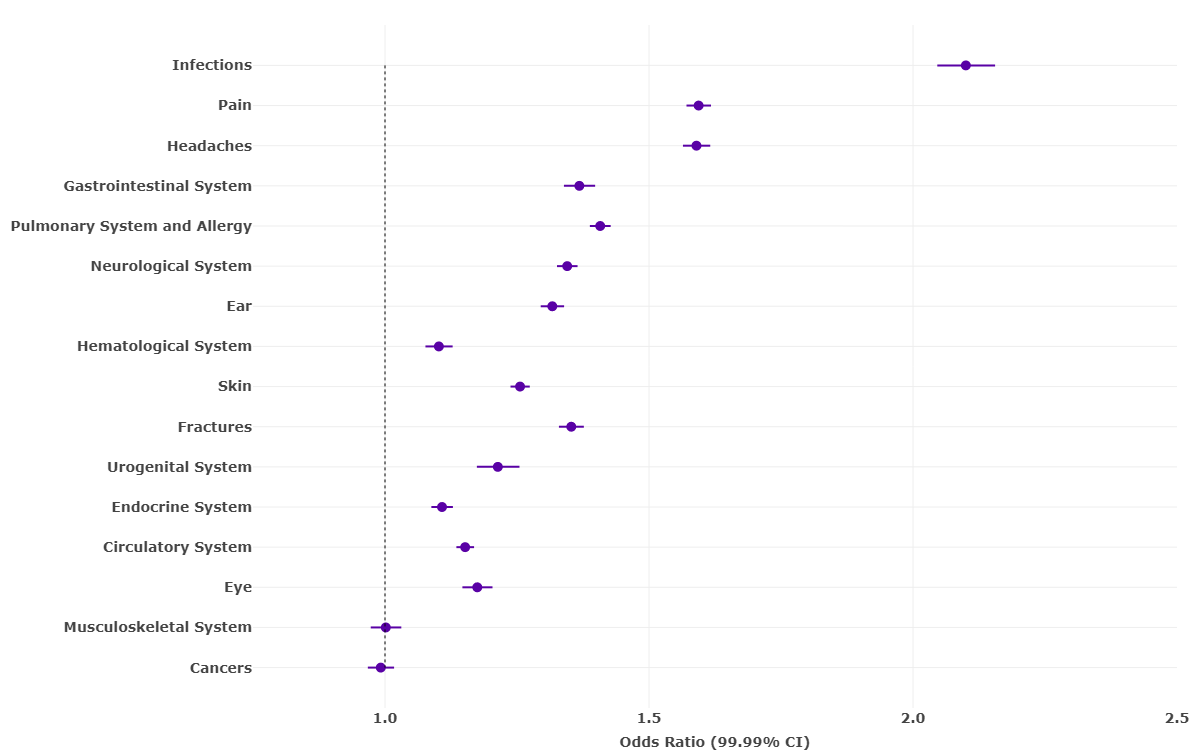 | 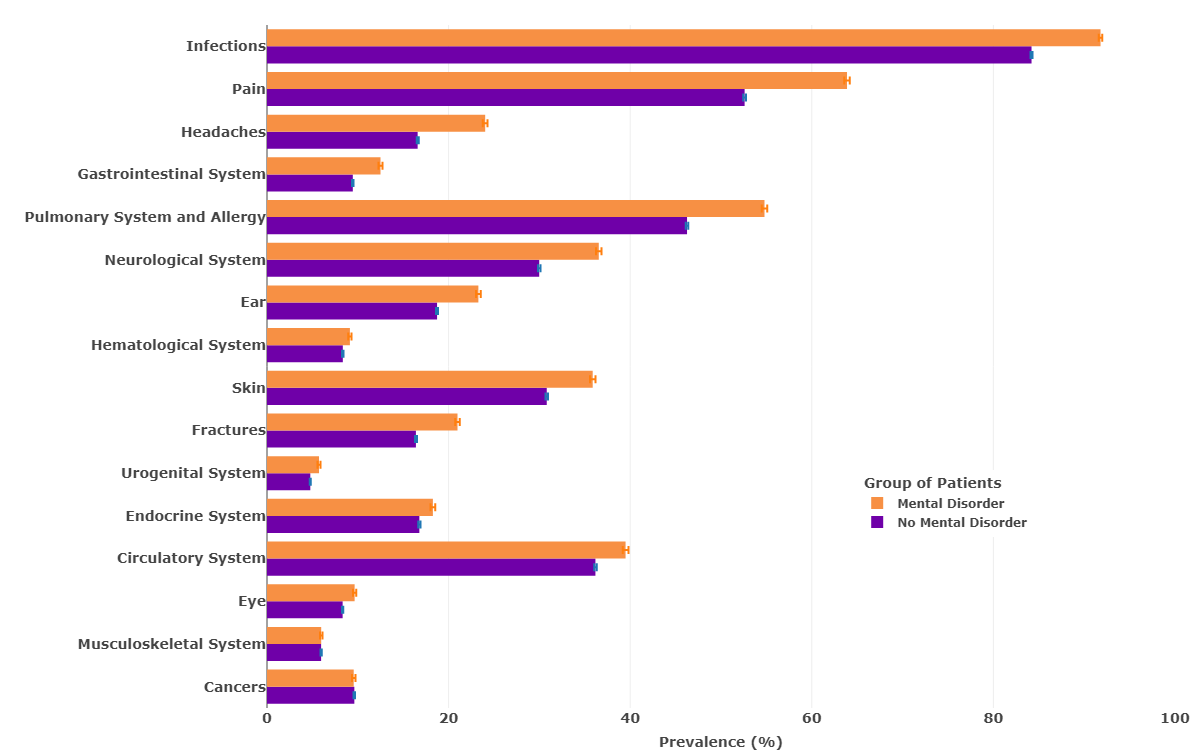 |

**Figure S5. Associations between substance abuse and 16 physical-health conditions**. **Panel A** shows associations (odds ratios and 99.99% confidence intervals) between substance abuse and 16 physical-health conditions in primary-care practices across 14 years. **Panel B** shows the risk of 16 physical-health conditions among patients who did and did not present with substance abuse. Inverse probability weighting was used to balance on four demographic variables: age at baseline, sex assigned at birth, educational attainment, and county of residence.

| **A. Associations between substance abuse and 16 physical-health conditions** | **B. Risk of 16 physical-health conditions for patients with and without substance abuse** |
| --- | --- |
| 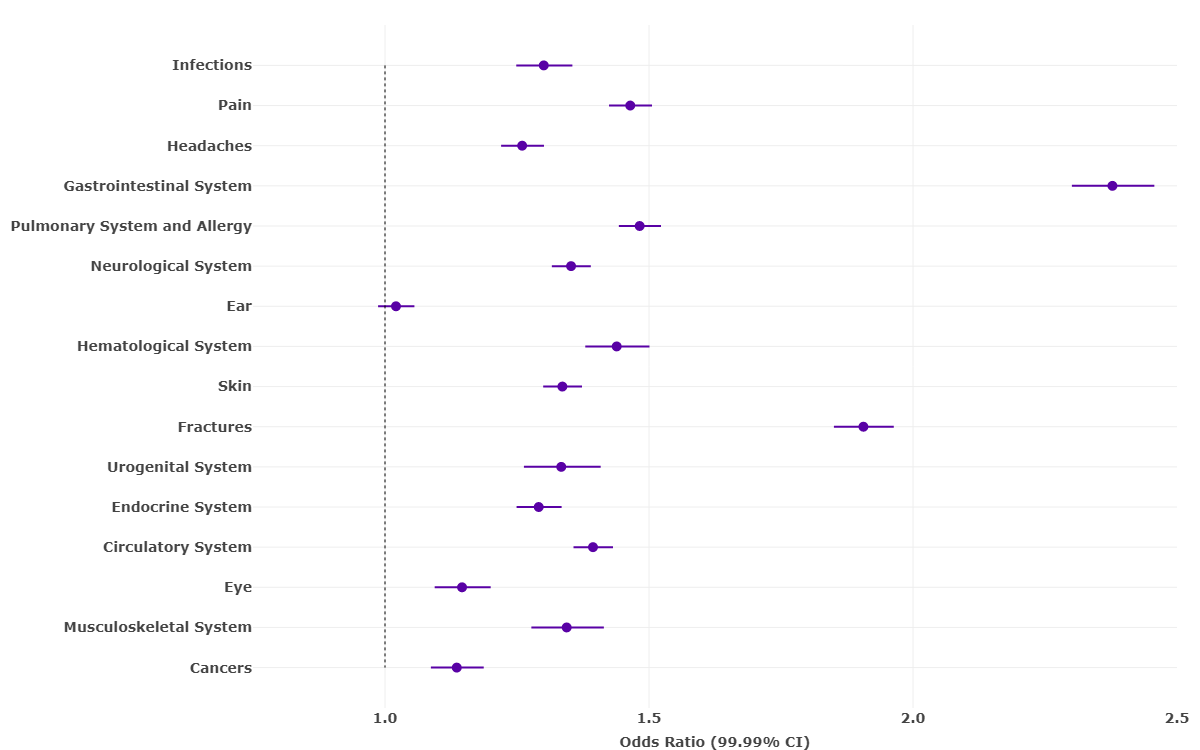 | 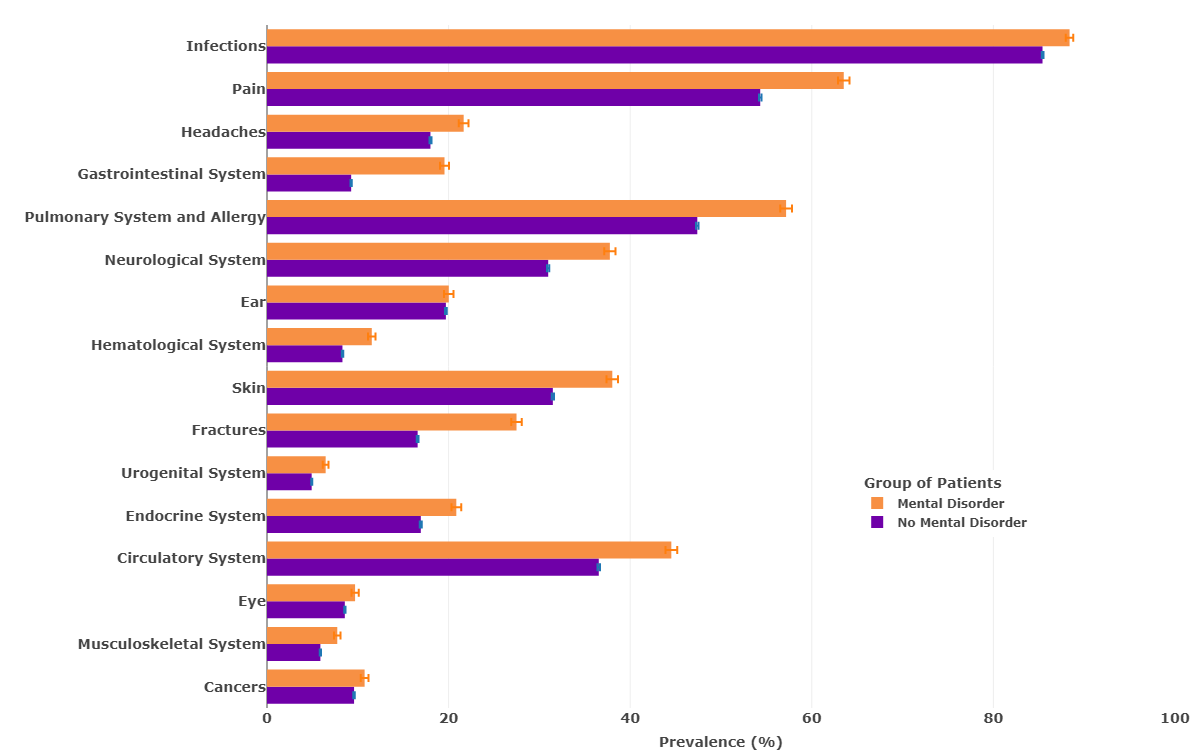 |

**Figure S6. Associations between phobia/compulsive disorder and 16 physical-health conditions**. **Panel A** shows associations (odds ratios and 99.99% confidence intervals) between phobia/compulsive disorder and 16 physical-health conditions in primary-care practices across 14 years. **Panel B** shows the risk of 16 physical-health conditions among patients who did and did not present with phobia/compulsive disorder. Inverse probability weighting was used to balance on four demographic variables: age at baseline, sex assigned at birth, educational attainment, and county of residence.

| **A. Associations between phobia/compulsive disorder and 16 physical-health conditions** | **B. Risk of 16 physical-health conditions for patients with and without phobia/compulsive disorder** |
| --- | --- |
| 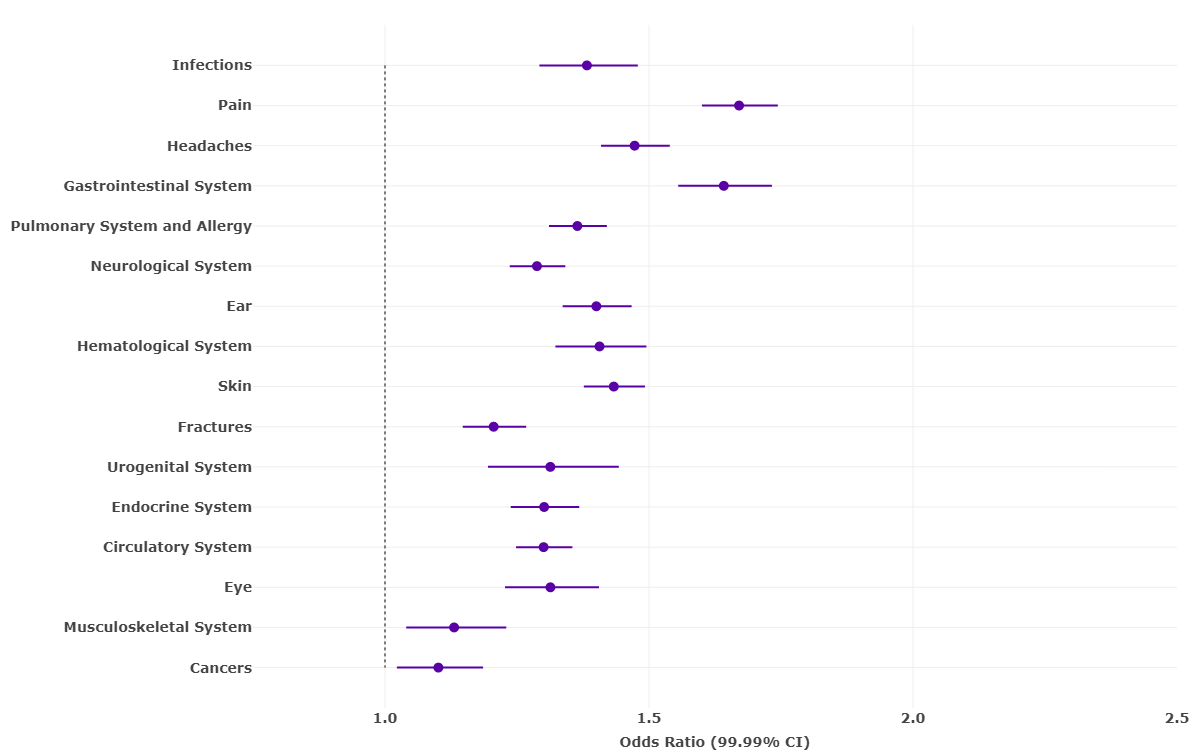 | 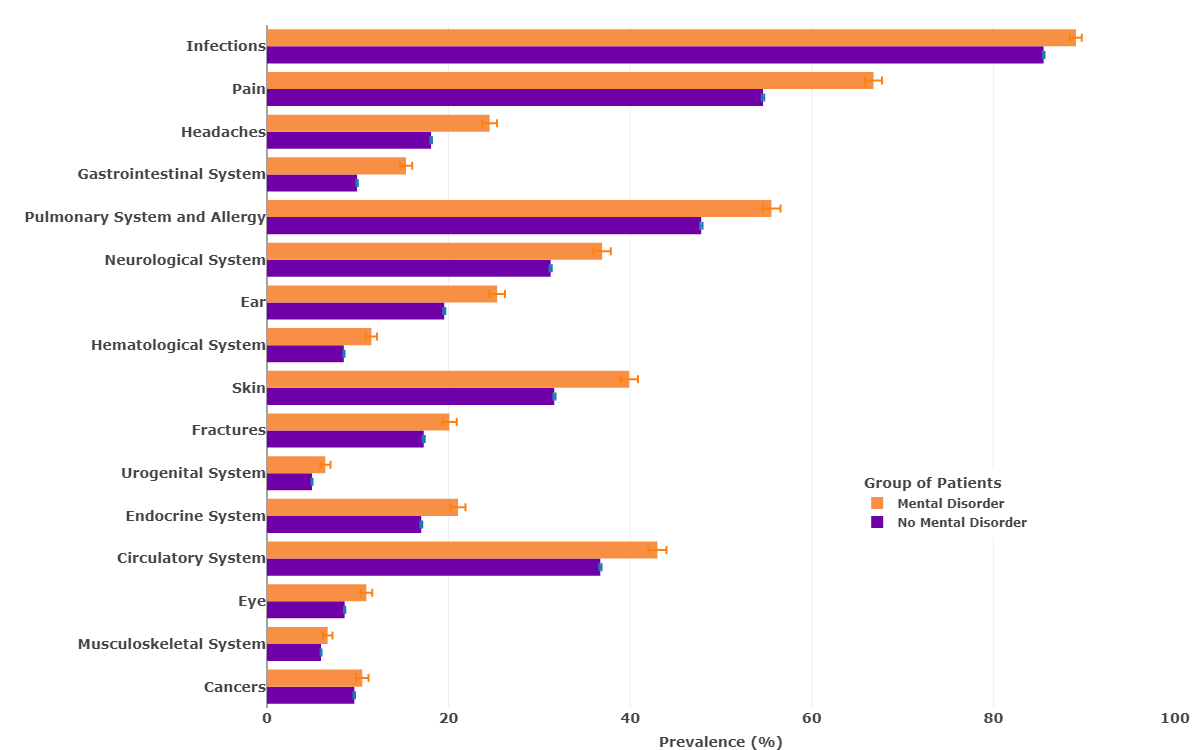 |

**Figure S7. Associations between psychosis and 16 physical-health conditions**. **Panel A** shows associations (odds ratios and 99.99% confidence intervals) between psychosis and 16 physical-health conditions in primary-care practices across 14 years. **Panel B** shows the risk of 16 physical-health conditions among patients who did and did not present with psychosis. Inverse probability weighting was used to balance on four demographic variables: age at baseline, sex assigned at birth, educational attainment, and county of residence.

| **A. Associations between psychosis and 16 physical-health conditions** | **B. Risk of 16 physical-health conditions for patients with and without psychosis** |
| --- | --- |
| 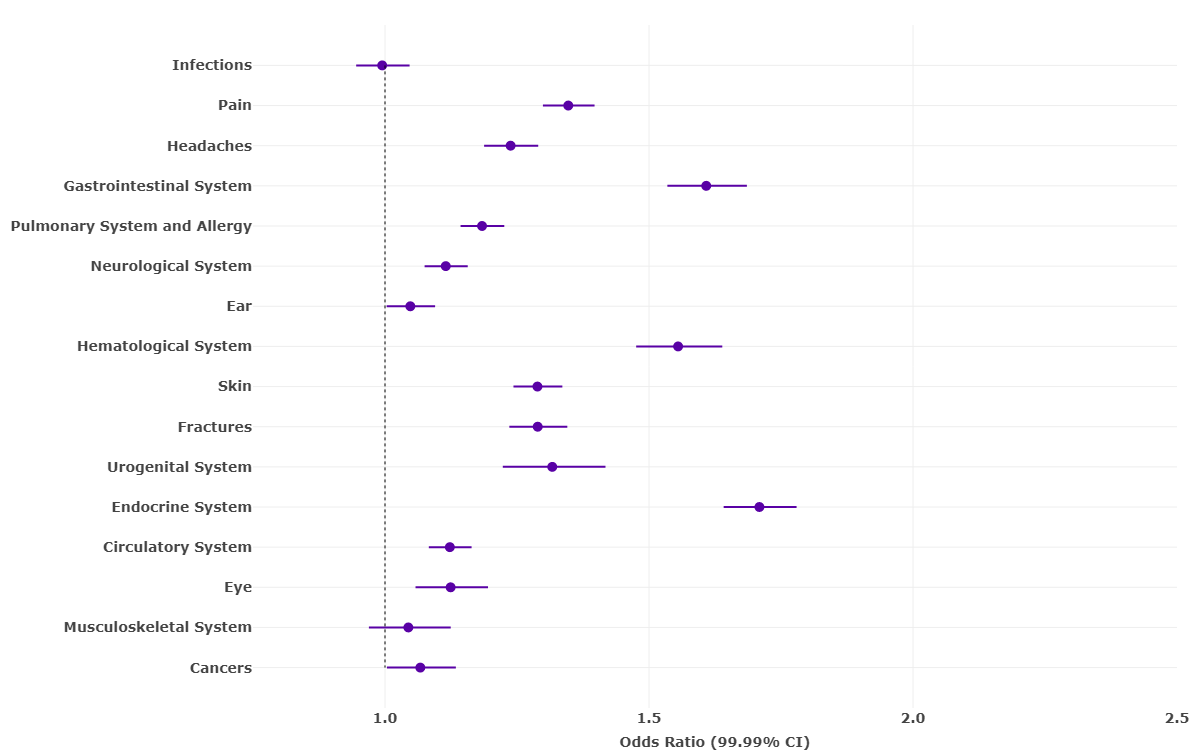 | 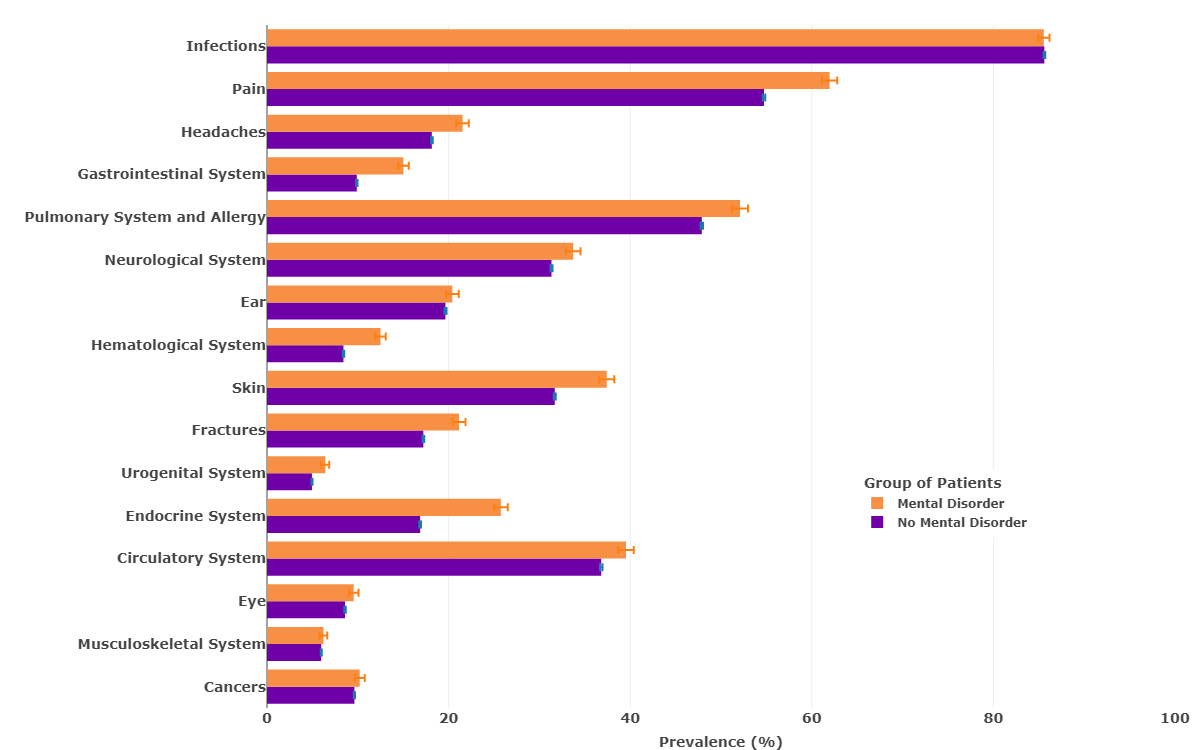 |

**Figure S8. Associations (odds ratios) between any mental disorder and 16 physical-health conditions across a 14-year period in male and female primary-care patients.** Inverse probability weighting was used to balance on three demographic variables: age at baseline, educational attainment, and county of residence. Connecting lines highlight the differences in odds ratios between females and males for each physical-health condition, visually representing the gender disparity in association strength. Point estimates and confidence intervals are provided in Supplemental Table S4.


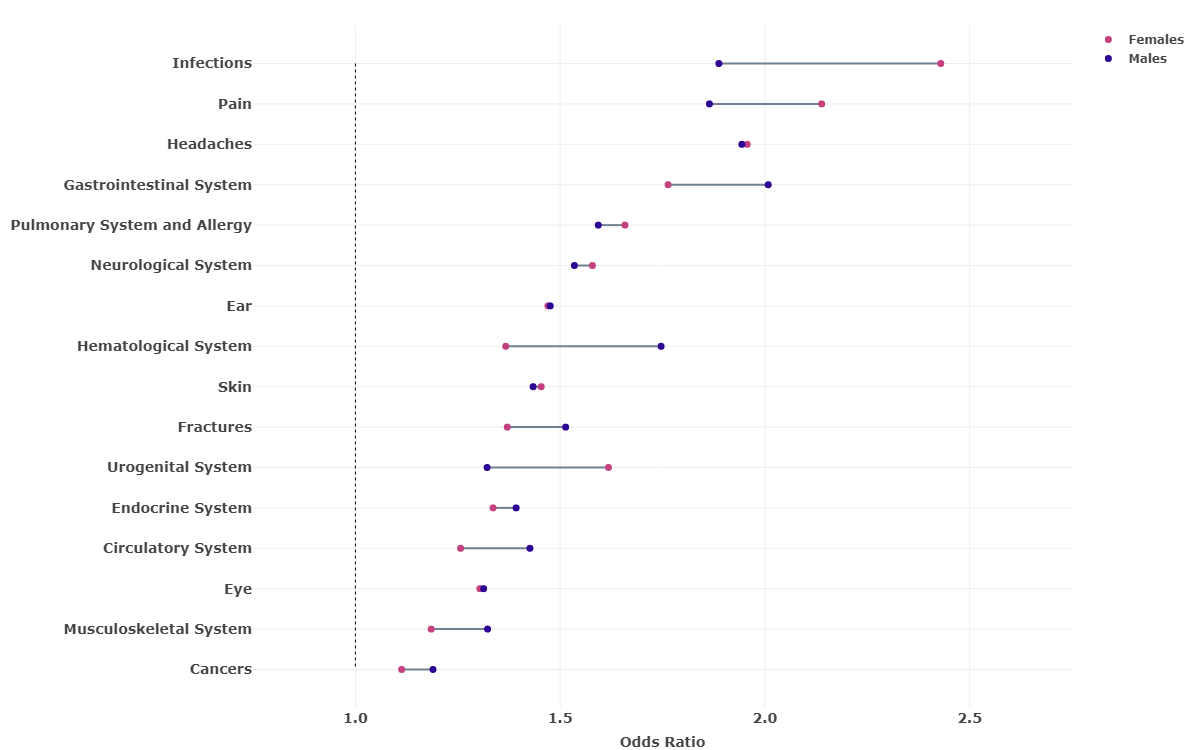


**Figure S9. Associations (odds ratios) between seven specific mental disorders and 16 physical-health conditions across a 14-year period in male and female primary-care patients.** Inverse probability weighting was used to balance on three demographic variables: age at baseline, educational attainment, and county of residence. Connecting lines highlight the differences in odds ratios between females and males for each physical-health condition, visually representing the gender disparity in association strength. Point estimates and confidence intervals are provided in Supplemental Table S4.

| **A. Sleep Disturbance** |
| --- |
| 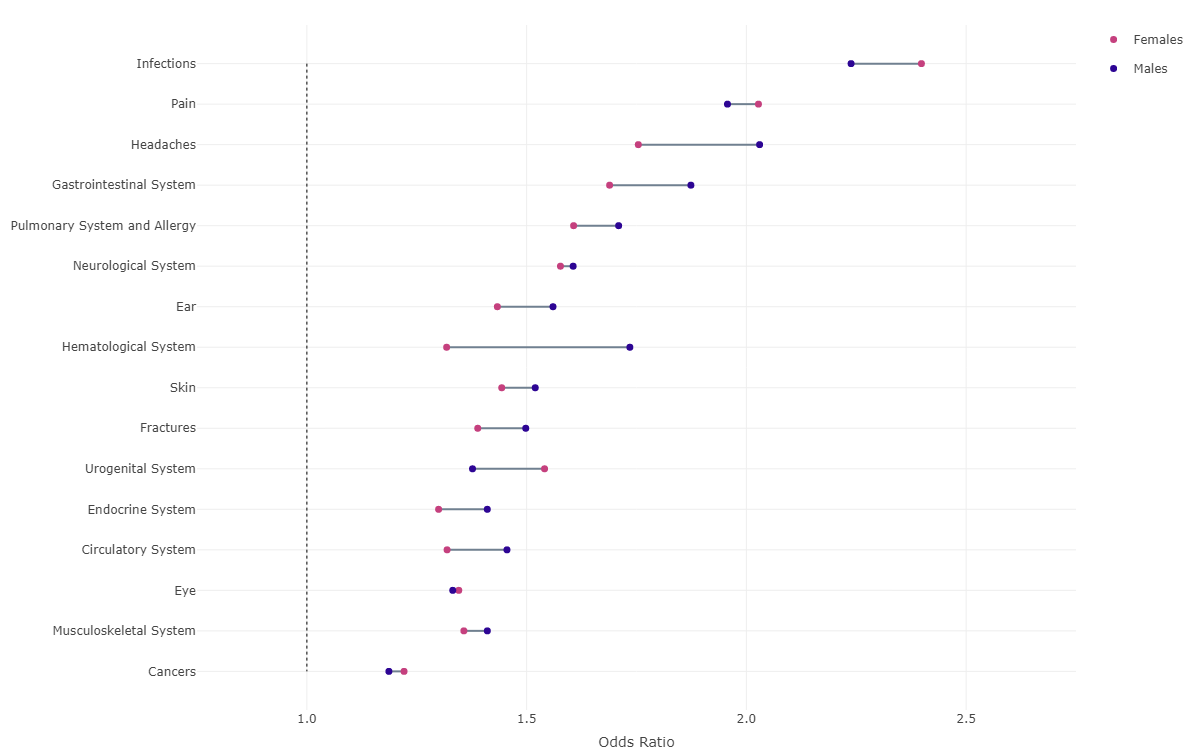 |

| **B. Anxiety** |
| --- |
| 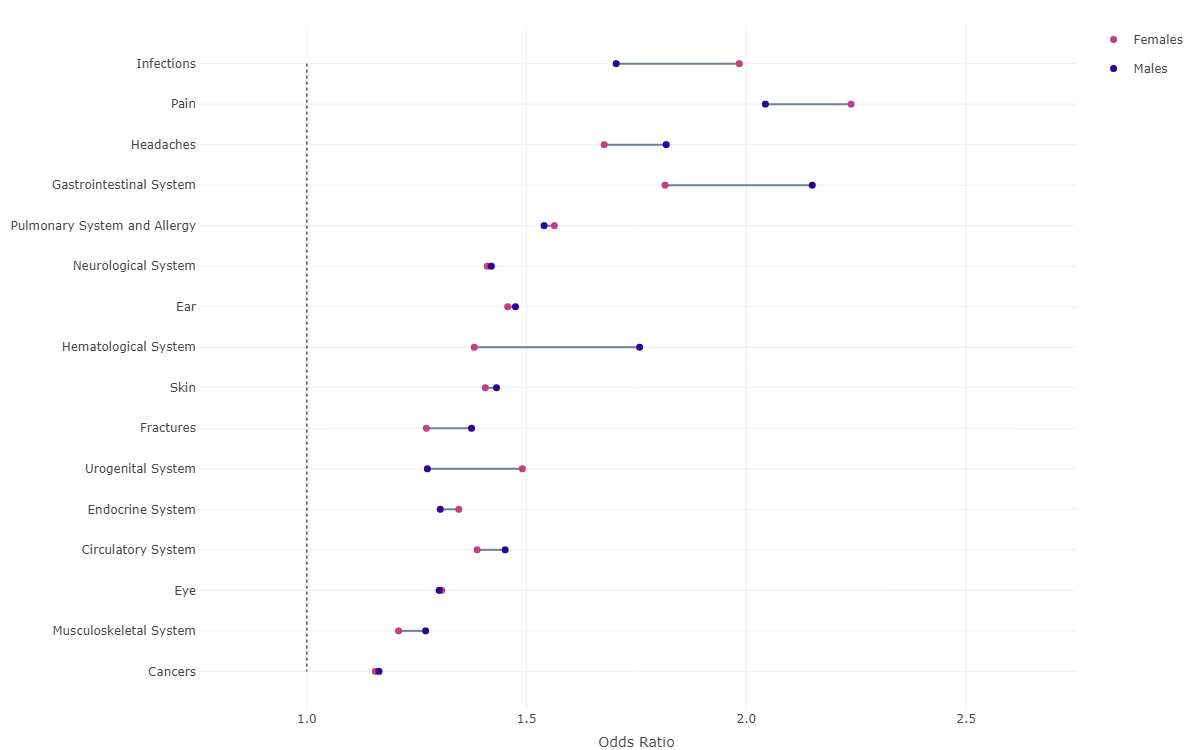 |
| **C. Depression** |
| 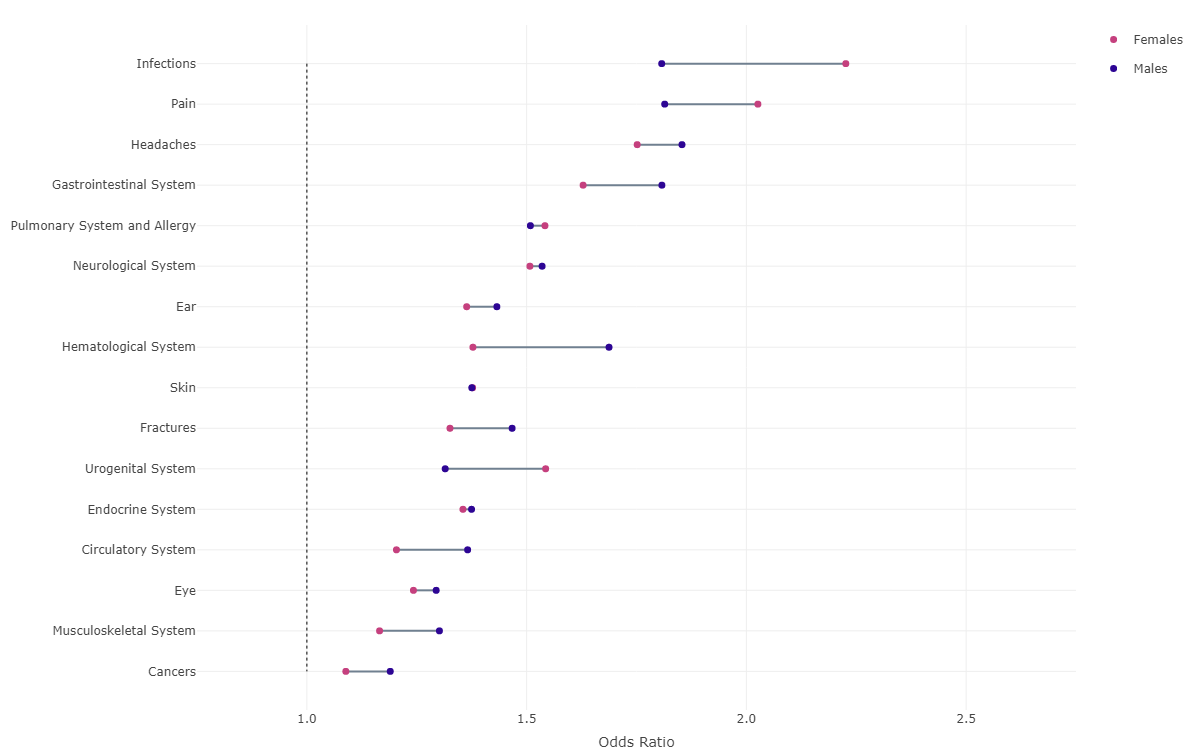 |

| **D. Acute Stress Reaction** |
| --- |
| 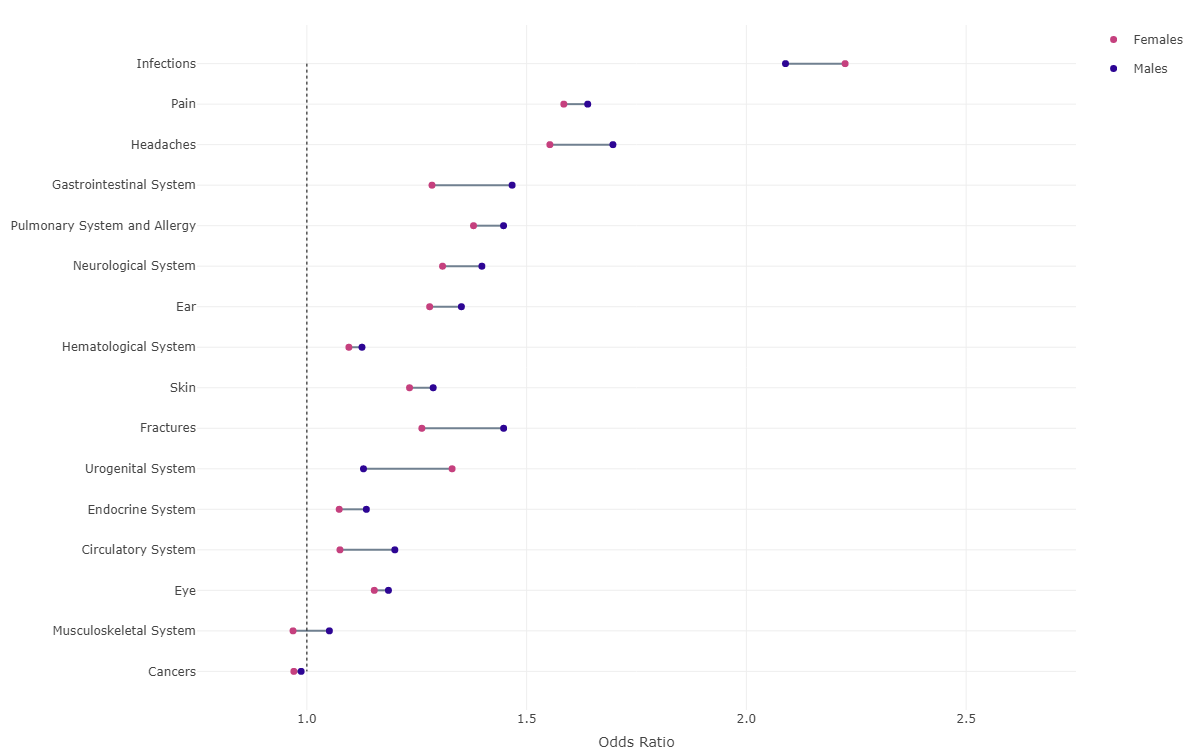 |
| **E. Substance Abuse** |
| 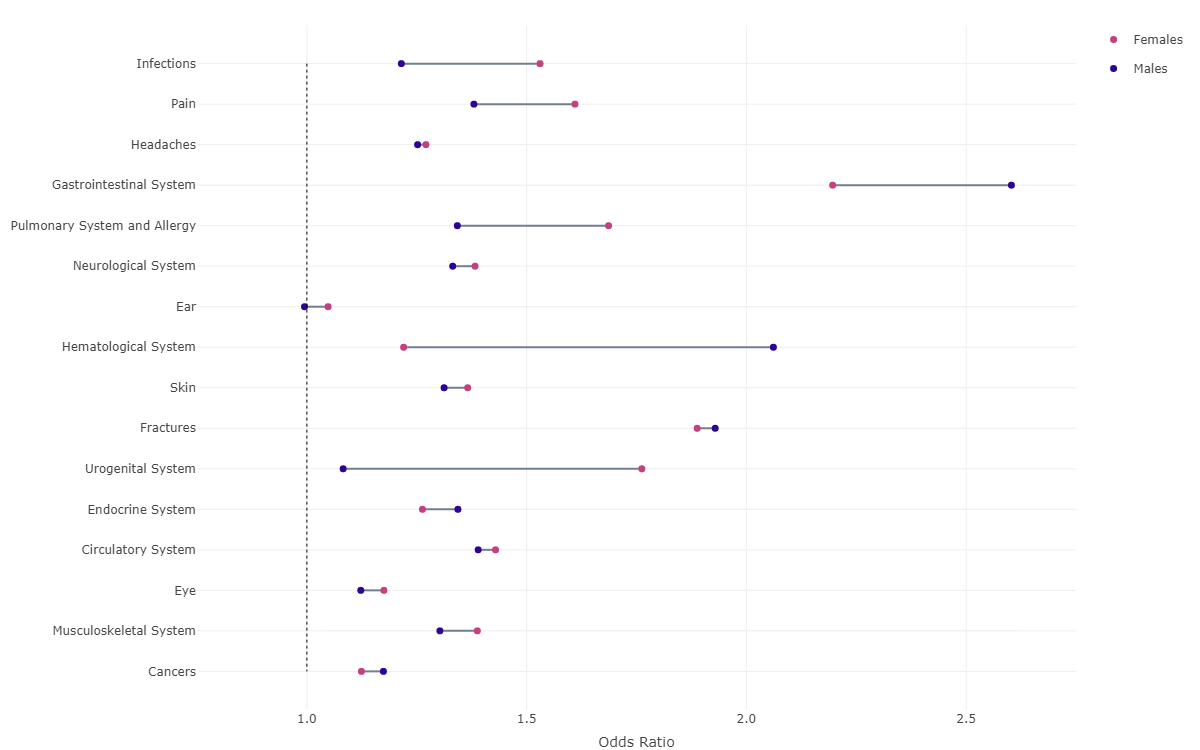 |

| **F. Phobia / Compulsive Disorder** |
| --- |
| 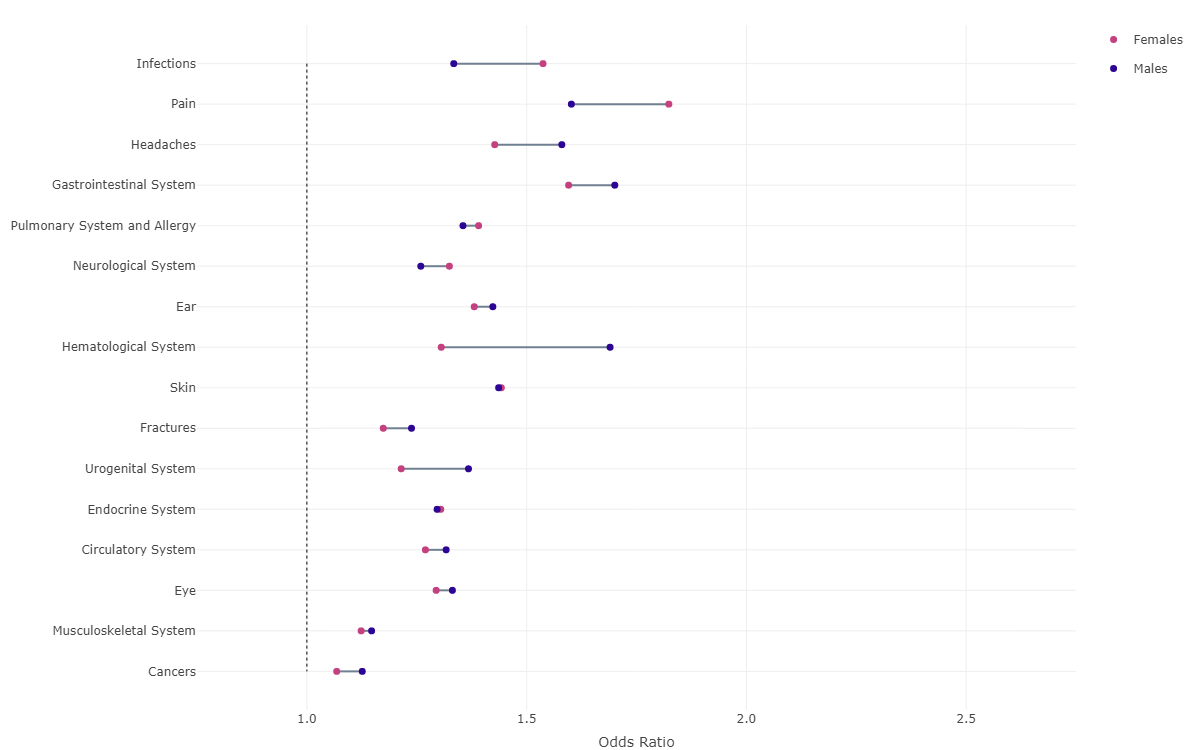 |
| **G. Psychosis** |
| 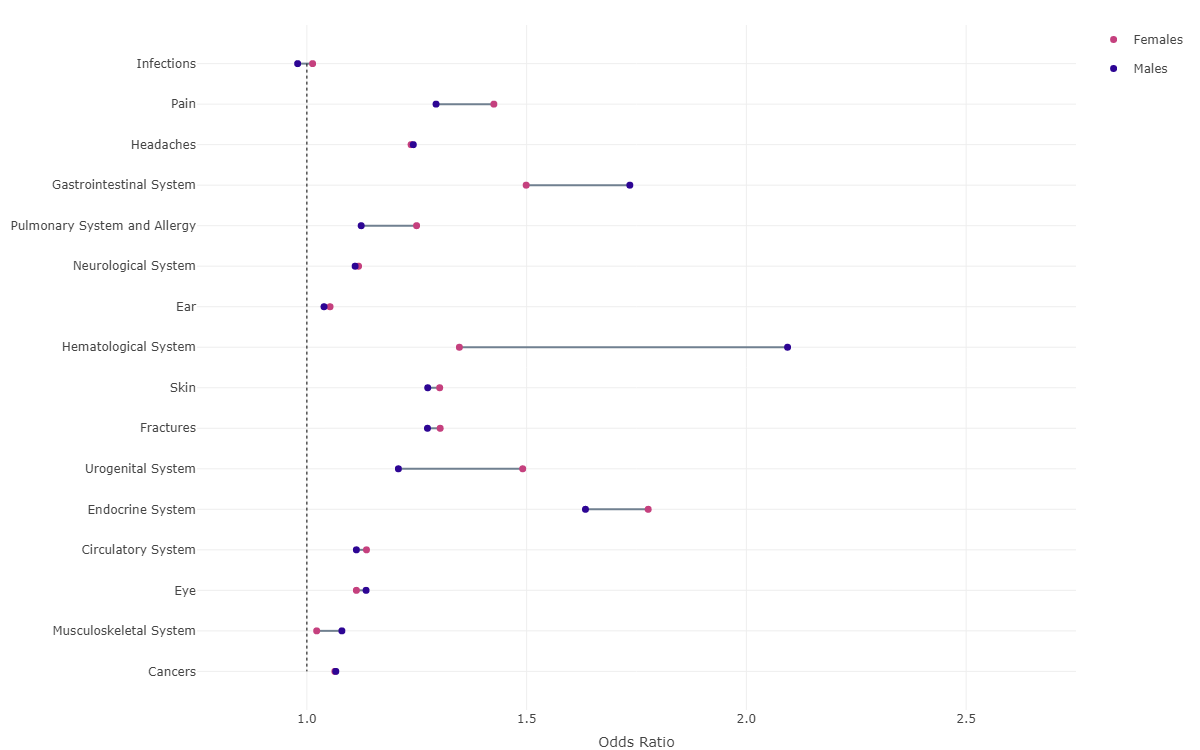 |

**Figure S10. Associations (odds ratios) between any mental disorders and 16 physical-health conditions across a 14-year period in younger and older (20-40 years vs 40-60 years at baseline) primary-care patients.** Inverse probability weighting was used to balance on three demographic variables: sex assigned at birth, educational attainment, and county of residence. Connecting lines highlight the differences in odds ratios between younger and older groups for each physical-health condition, visually representing the age disparity in association strength. Point estimates and confidence intervals are provided in Supplemental Table S5.


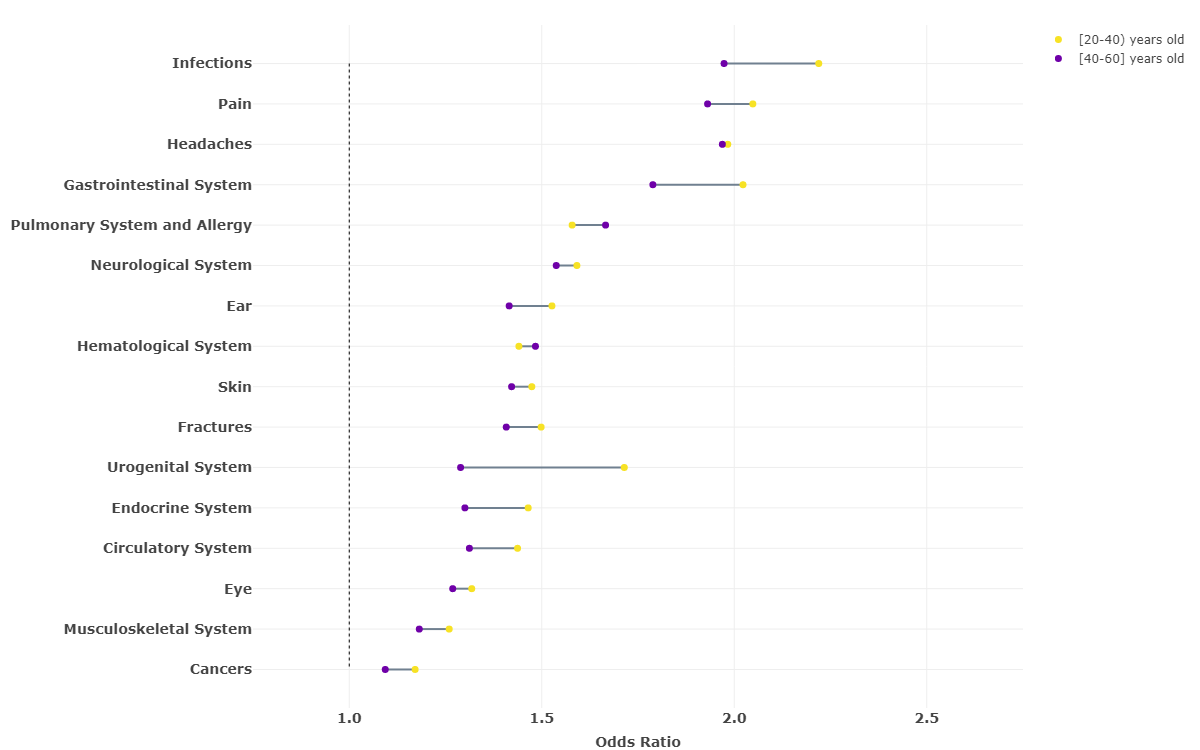


**Figure S11. Associations (odds ratios) between seven specific mental disorders and 16 physical-health conditions across a 14-year period in younger and older (20-40 years vs 40-60 years at baseline) primary-care patients.** Inverse probability weighting was used to balance on three demographic variables: sex assigned at birth, educational attainment, and county of residence. Connecting lines highlight the differences in odds ratios between younger and older groups for each physical-health condition, visually representing the age disparity in association strength. Point estimates and confidence intervals are provided in Supplemental Table S5.

| **A. Sleep Disturbance** |
| --- |
| 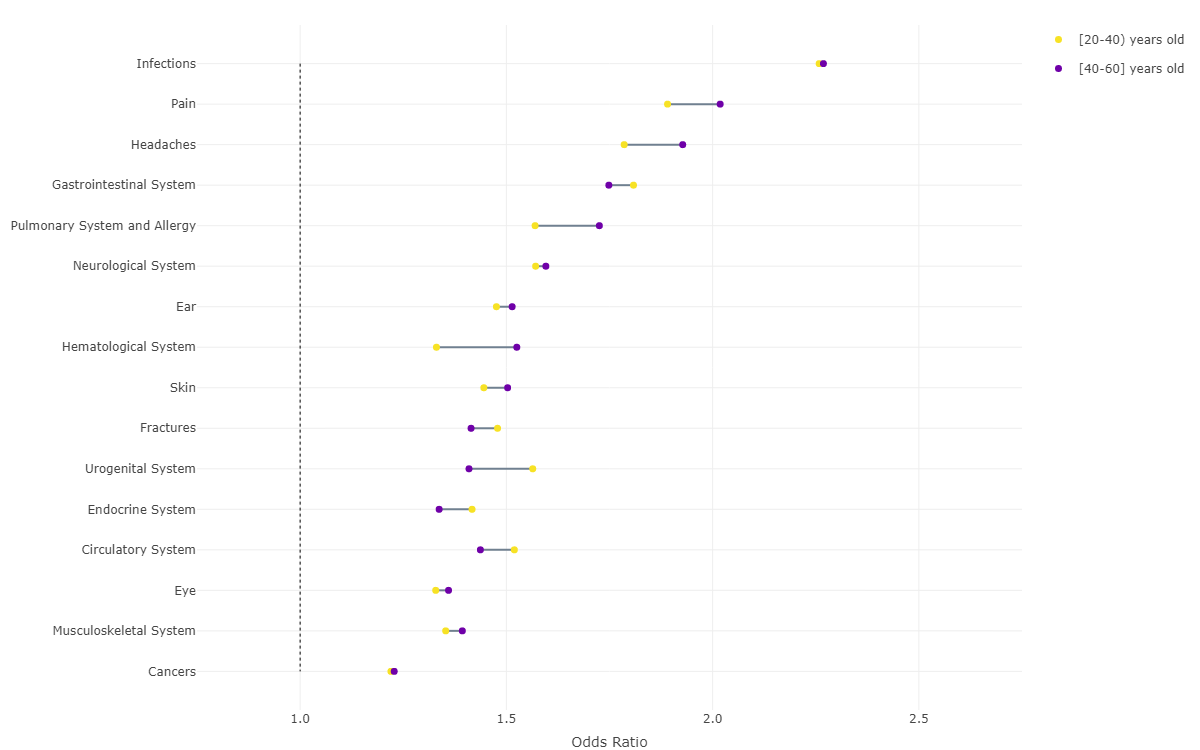 |

| **B. Anxiety** |
| --- |
| 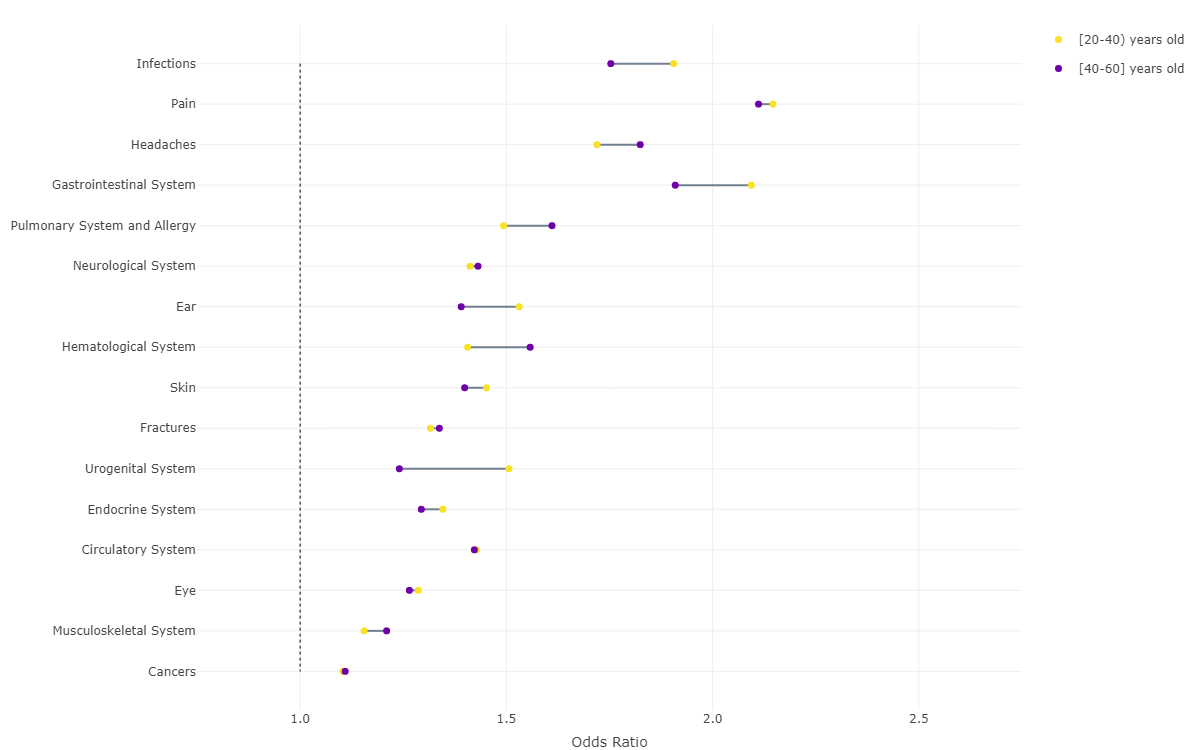 |
| **C. Depression** |
| 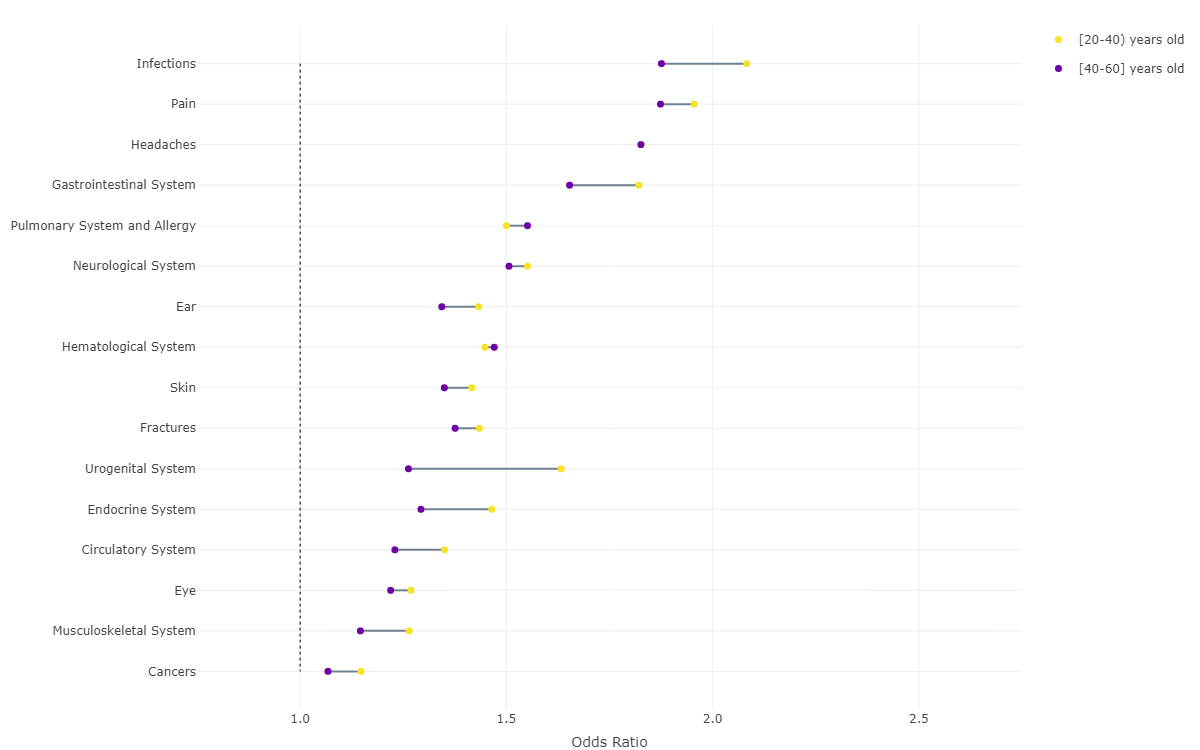 |

| **D. Acute Stress Reaction** |
| --- |
| 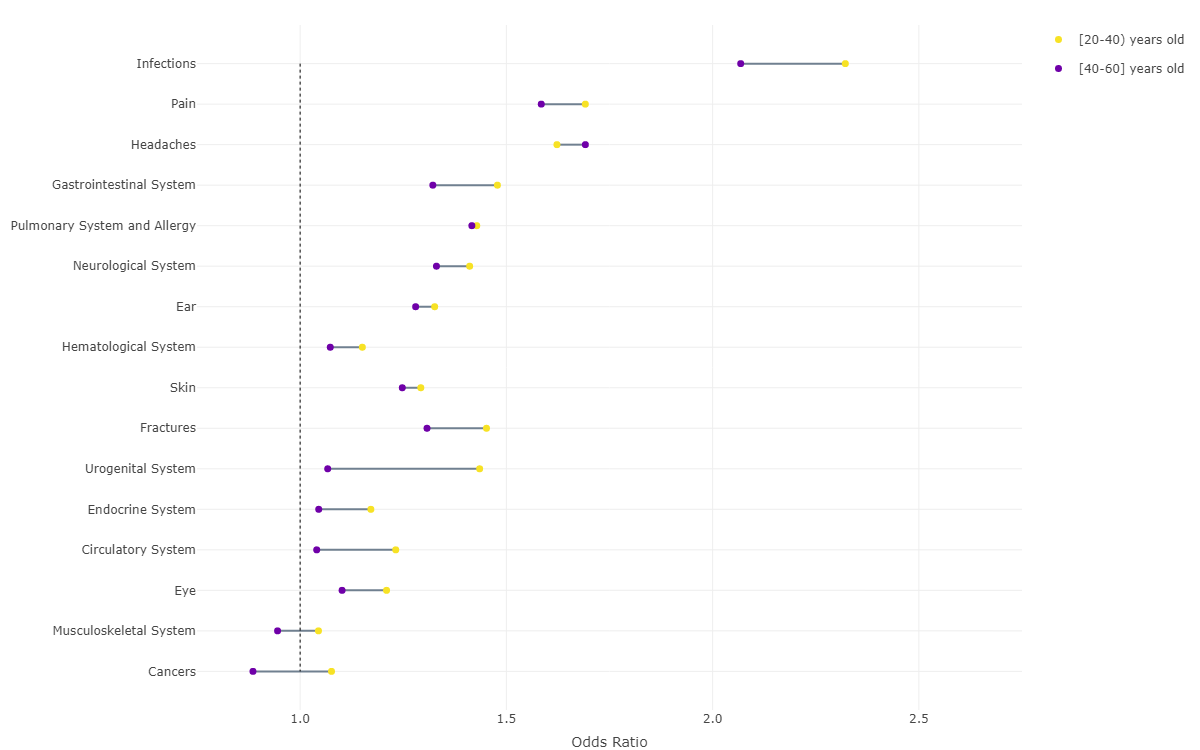 |
| **E. Substance Abuse** |
| 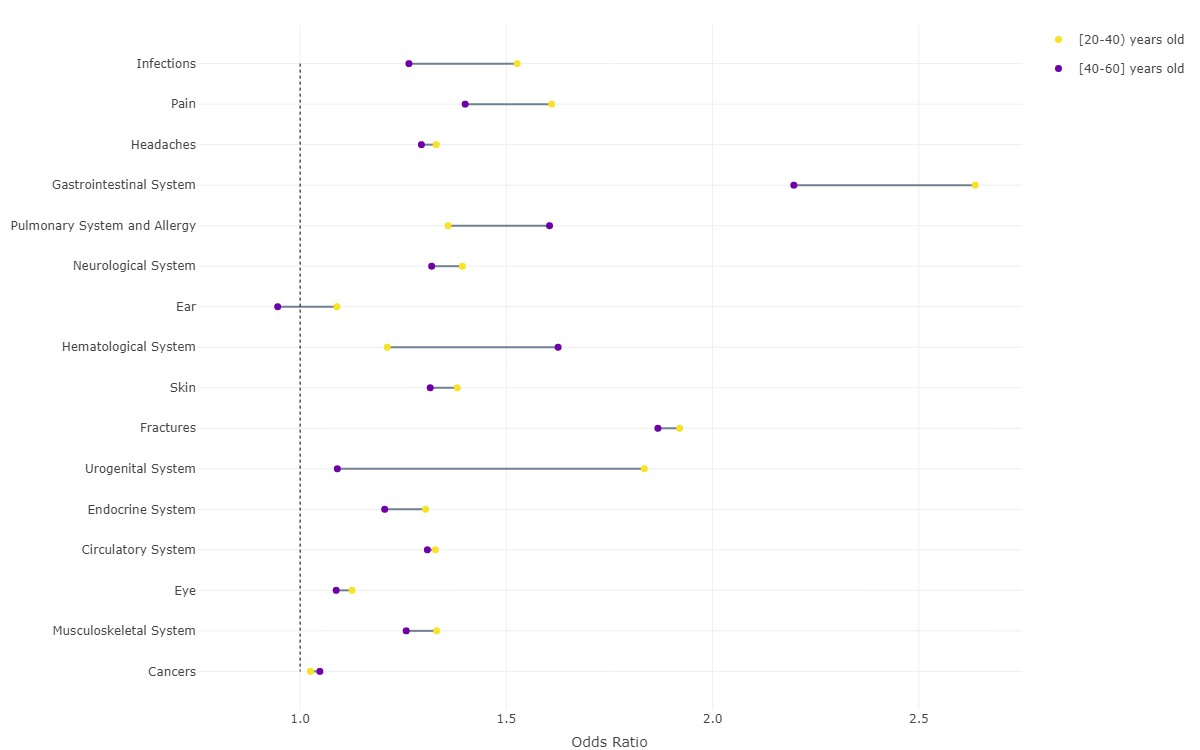 |

| **F. Phobia / Compulsive Disorder** |
| --- |
| 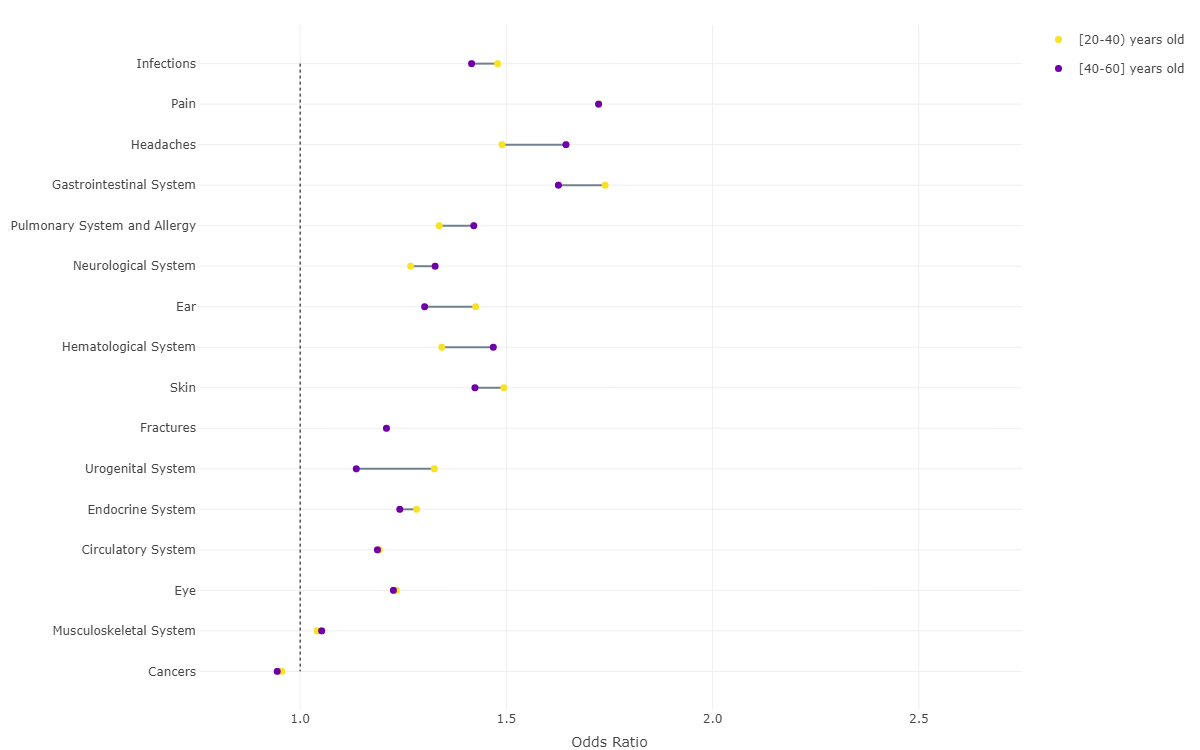 |
| **G. Psychosis** |
| 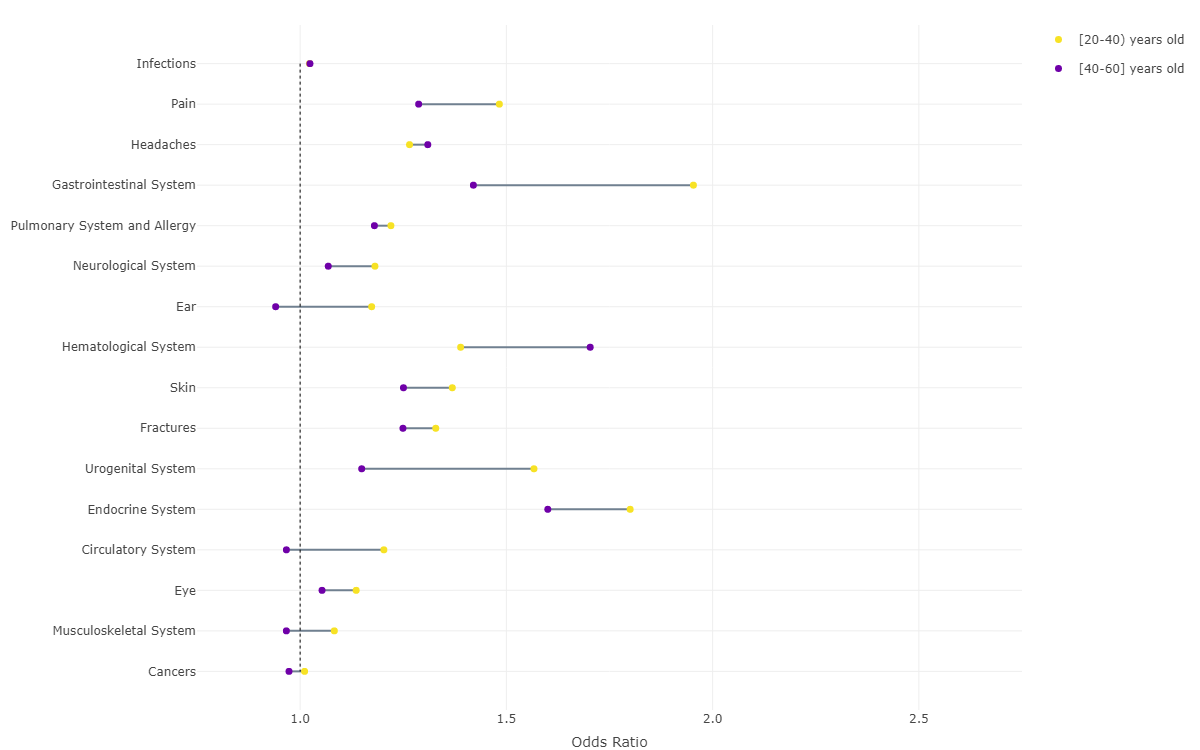 |

**Figure S12. Associations (odds ratios) between any mental disorder and 16 physical-health conditions across a 14-year period in primary-care patients who did and did not attend college.** Inverse probability weighting was used to balance on three demographic variables: sex assigned at birth, age at baseline, and county of residence. Connecting lines highlight the differences in odds ratios between groups for each physical-health condition, visually representing the education disparity in association strength. Point estimates and confidence intervals are provided in Supplemental Table S6.


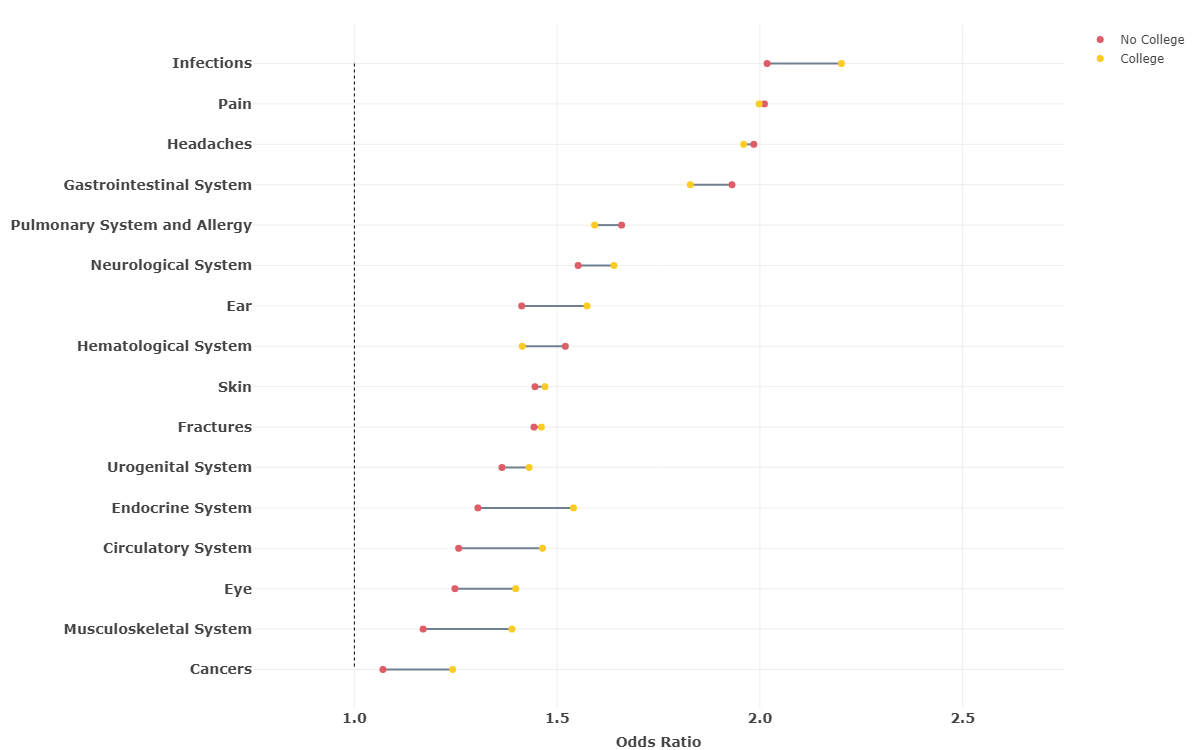


**Figure S13. Associations (odds ratios) between seven specific mental disorders and 16 physical-health conditions across a 14-year period in primary-care patients who did and did not attend college.** Inverse probability weighting was used to balance on three demographic variables: sex assigned at birth, age at baseline, and county of residence. Connecting lines highlight the differences in odds ratios between groups for each physical-health condition, visually representing the education disparity in association strength. Point estimates and confidence intervals are provided in Supplemental Table S6.

| **A. Sleep Disturbance** |
| --- |
| 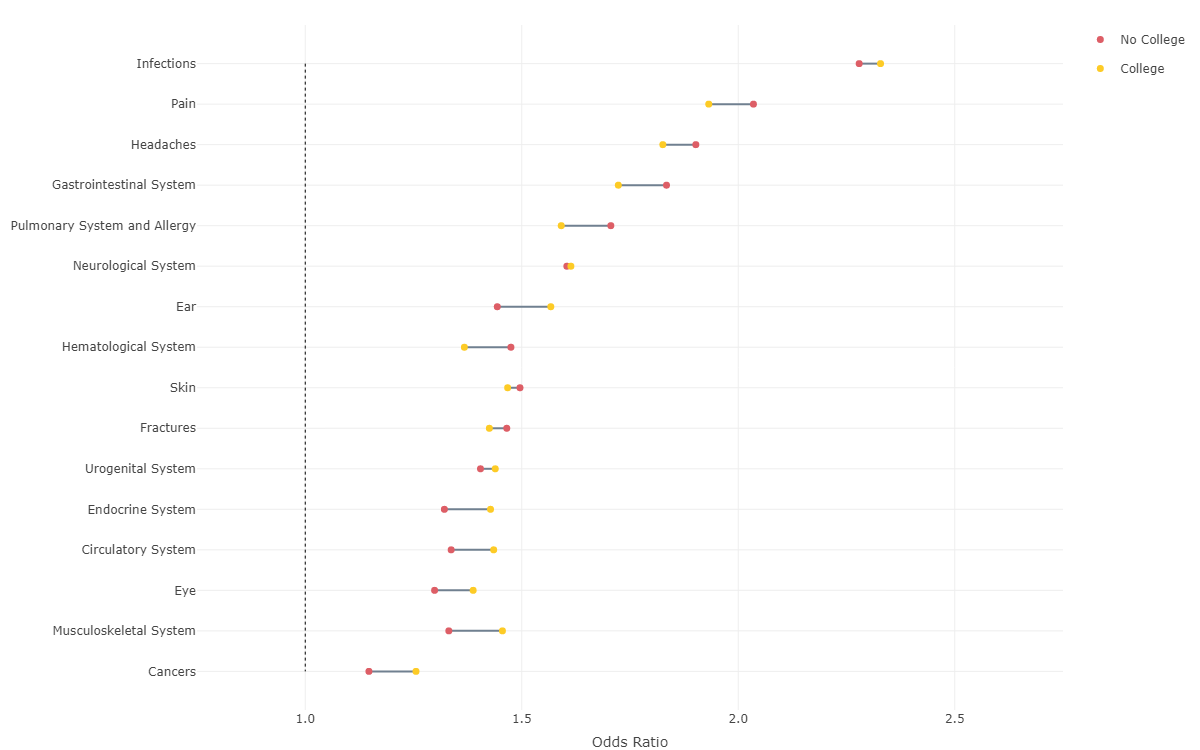 |

| **B. Anxiety** |
| --- |
| 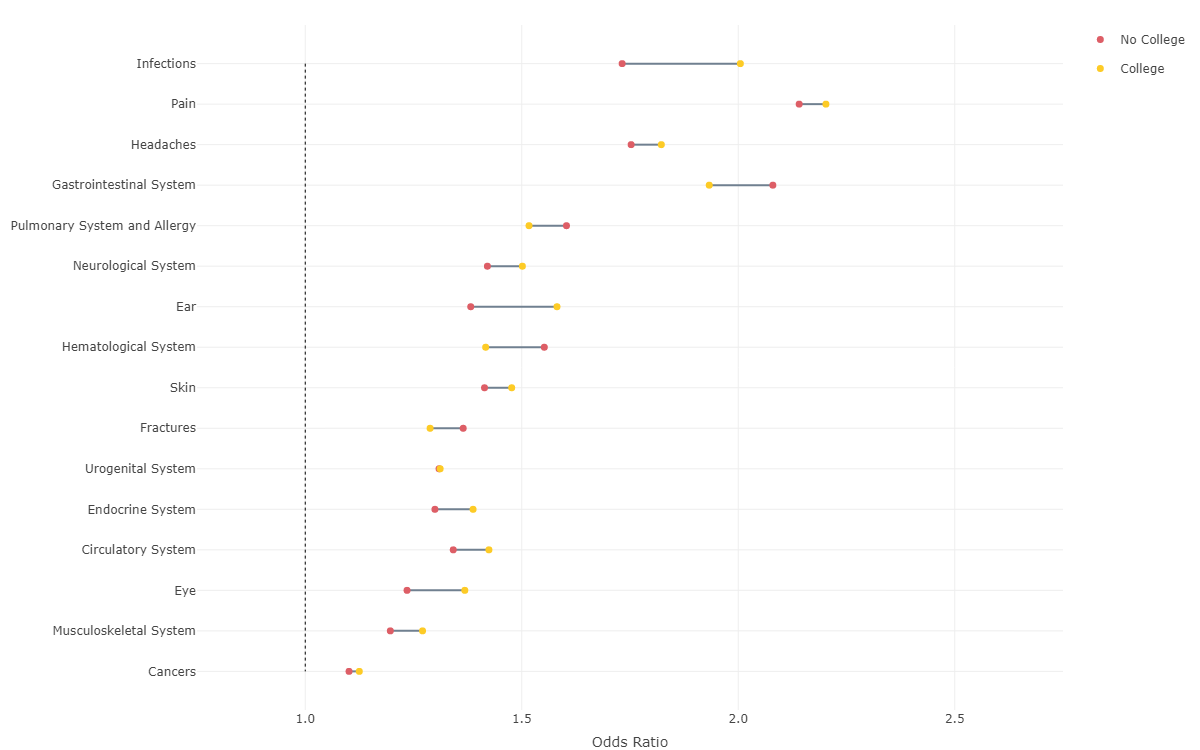 |
| **C. Depression** |
| 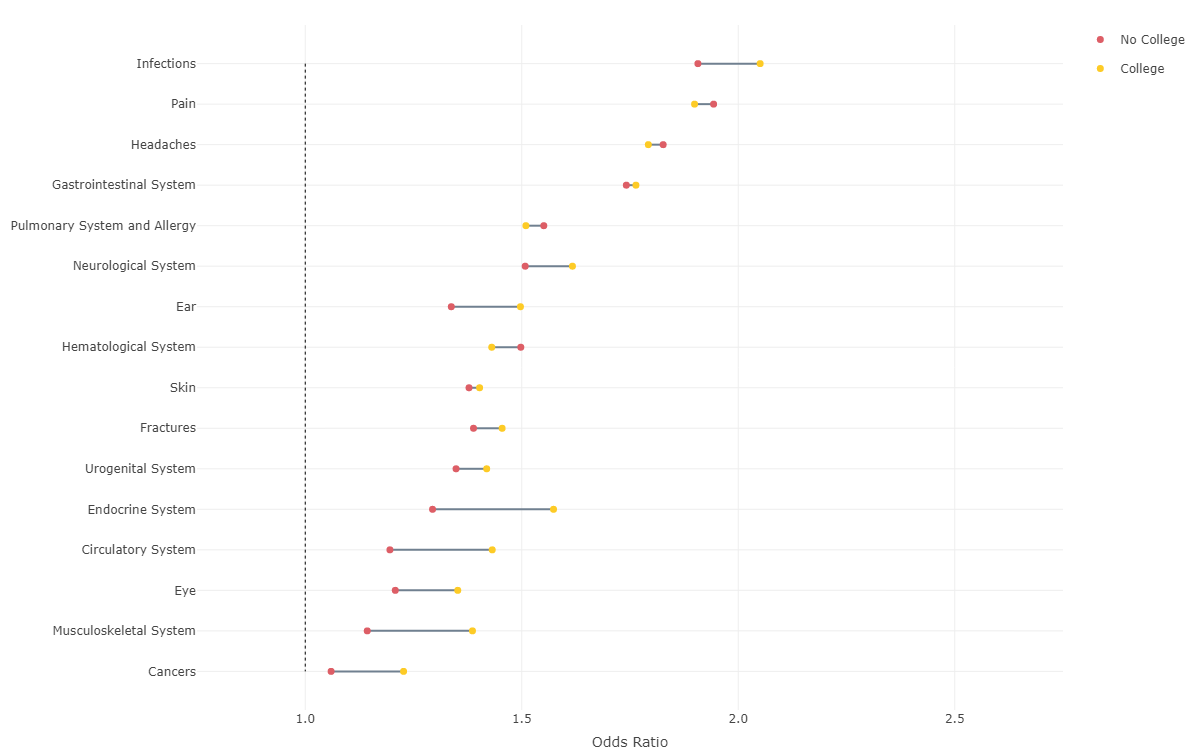 |

| **D. Acute Stress Reaction** |
| --- |
| 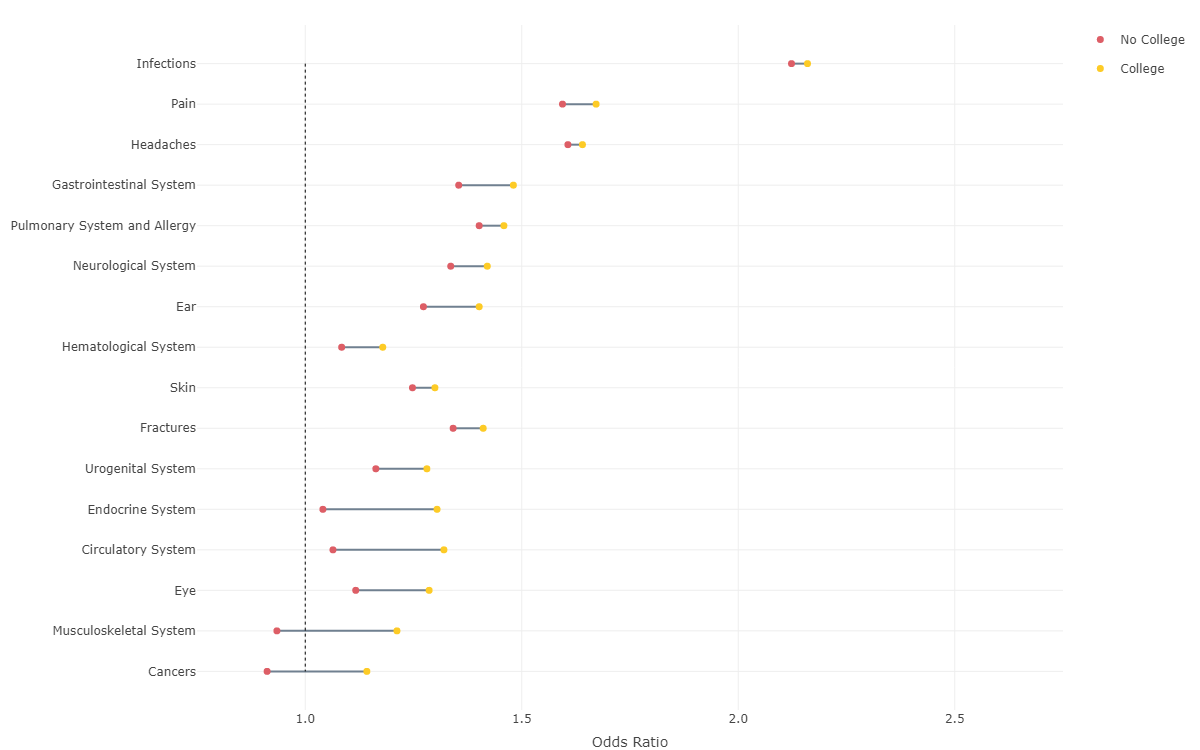 |
| **E. Substance Abuse** |
| 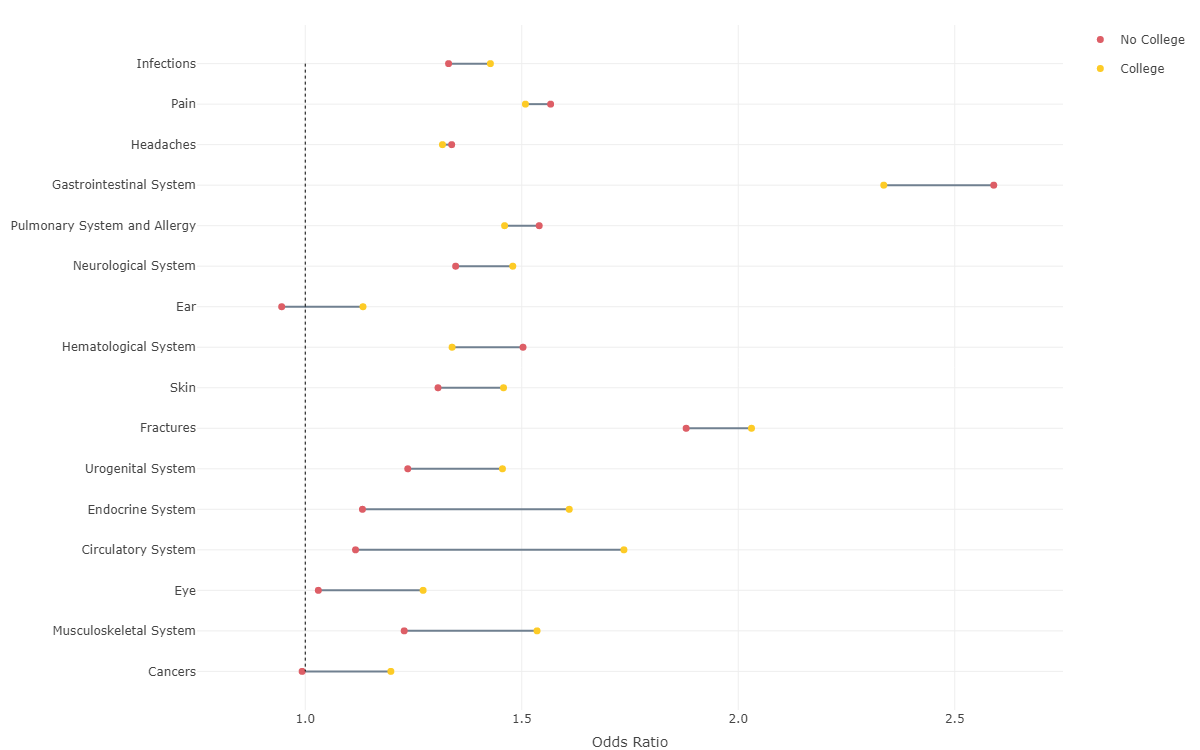 |

| **F. Phobia / Compulsive Disorder** |
| --- |
| 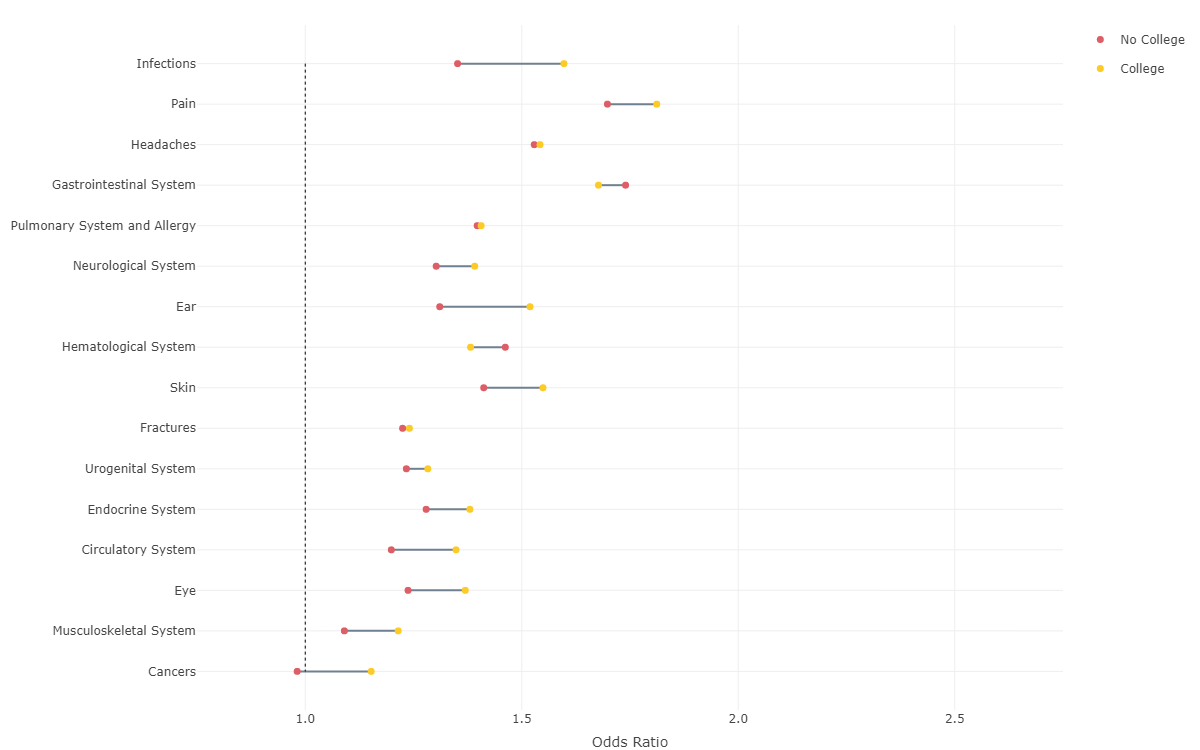 |
| **G. Psychosis** |
| 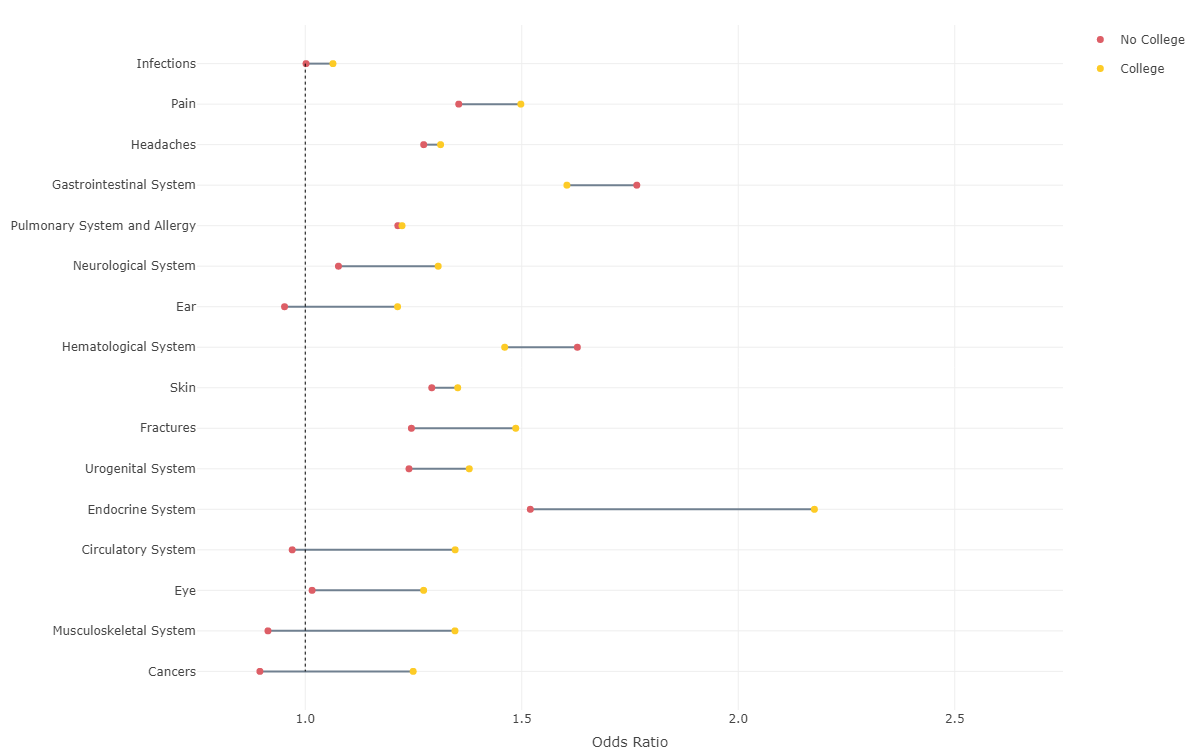 |

**Figure S14. Risk (hazard ratios) of 16 physical-health conditions after diagnosis of seven specific mental disorders across a 14-year period in primary-care patients.** Inverse probability weights were used to balance on four demographic variables: age at baseline, sex assigned at birth, educational attainment, and county of residence. Within each month from January 2006 through December 2019, encounters with primary-care providers were assessed for mental disorders and physical-health conditions. Extended Cox proportional hazards models were used, treating dependent time-to-event variables and independent exposures as recurrent and time-varying, respectively.

| **A. Sleep Disturbance** |
| --- |
| 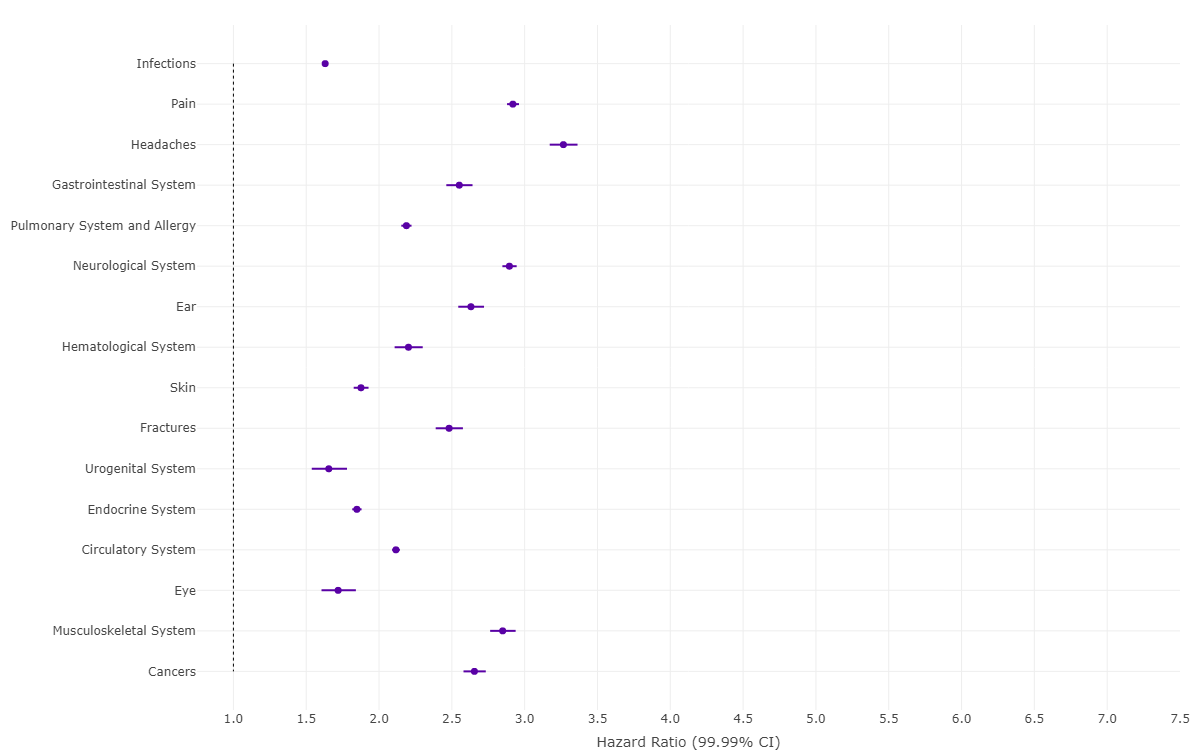 |

| **B. Anxiety** |
| --- |
| 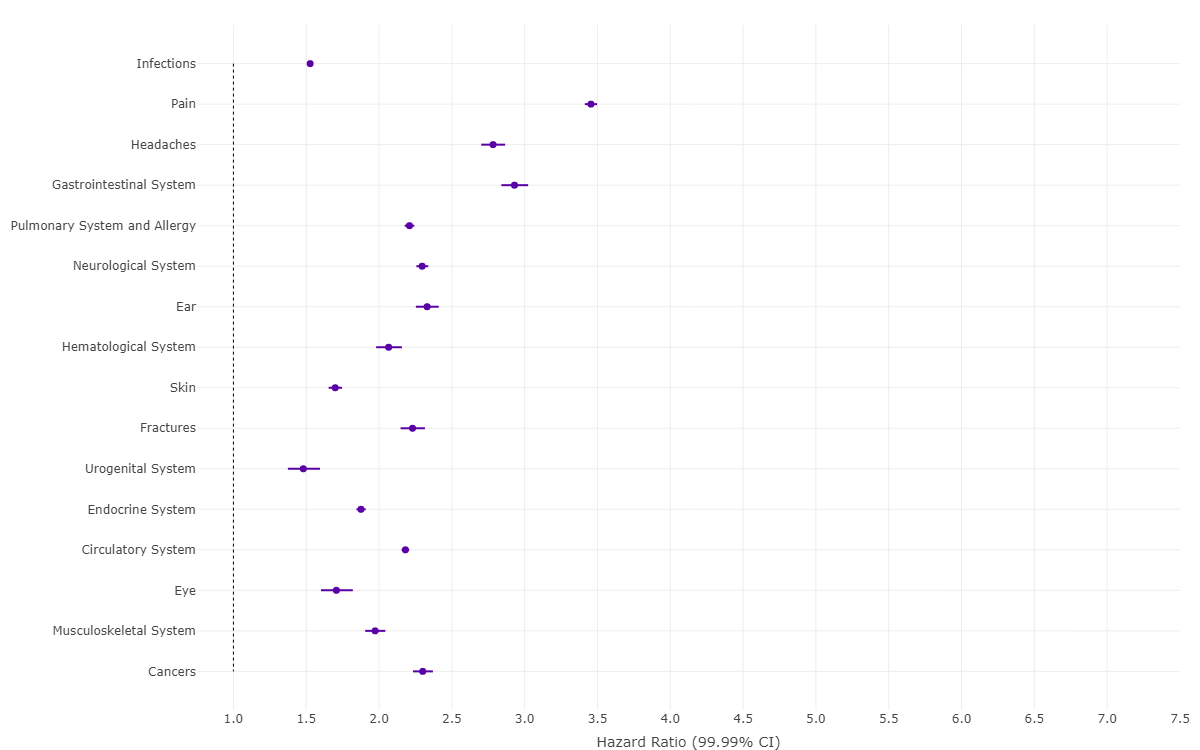 |
| **C. Depression** |
| 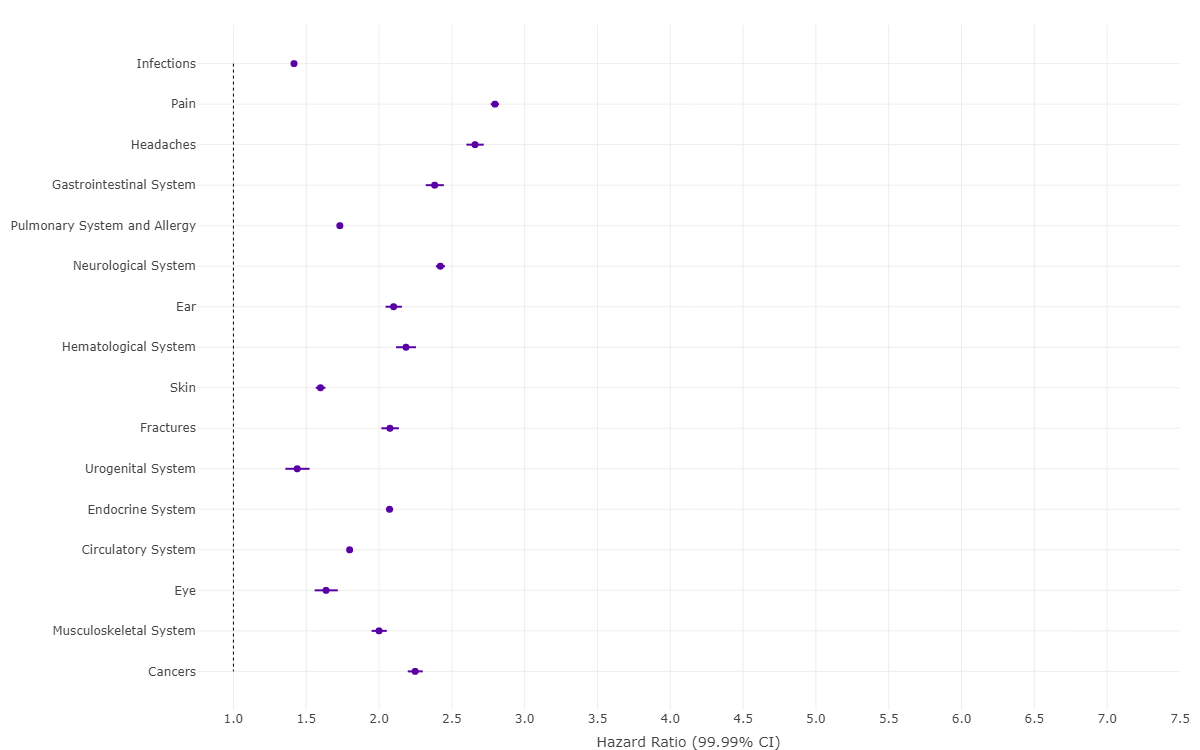 |

| **D. Acute Stress Reaction** |
| --- |
| 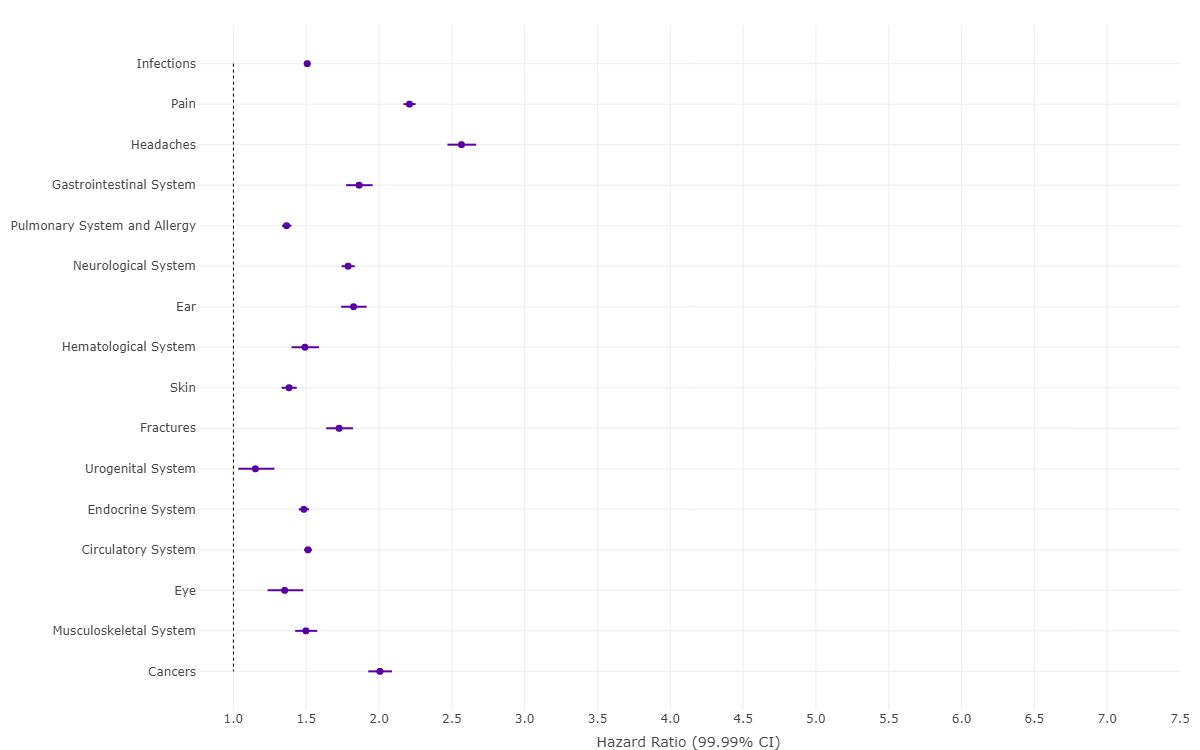 |
| **E. Substance Abuse** |
| 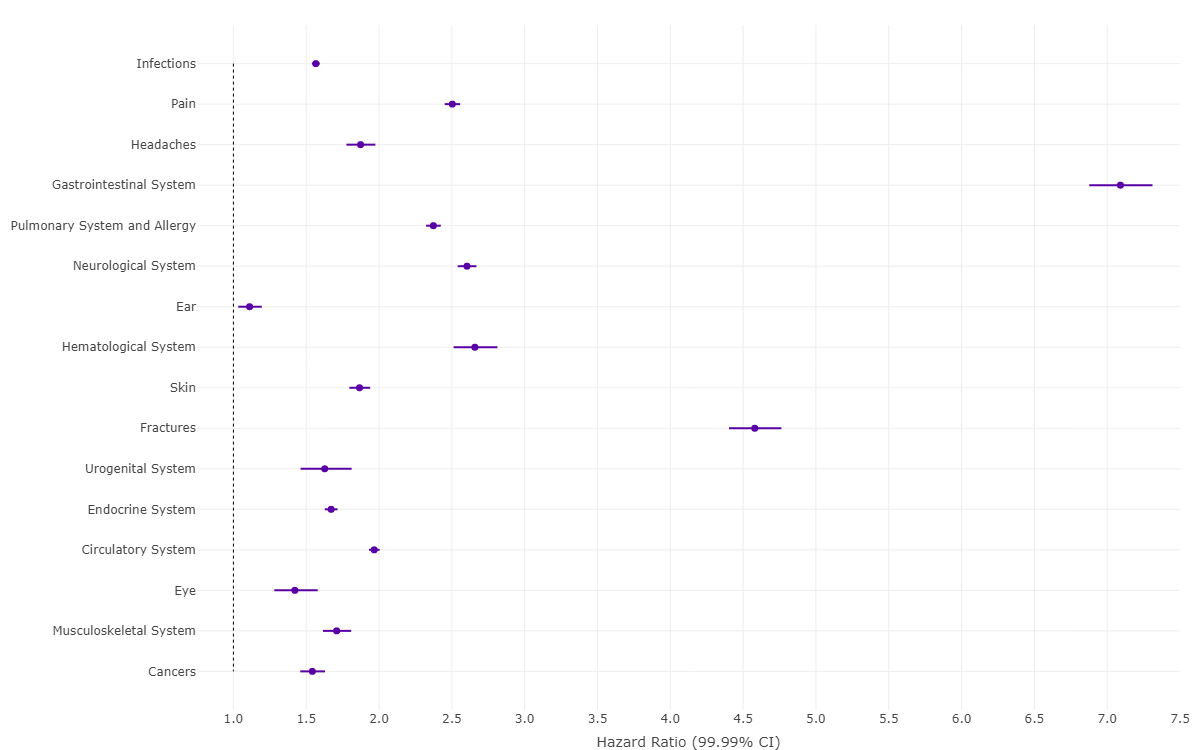 |

| **F. Phobia / Compulsive Disorder** |
| --- |
| 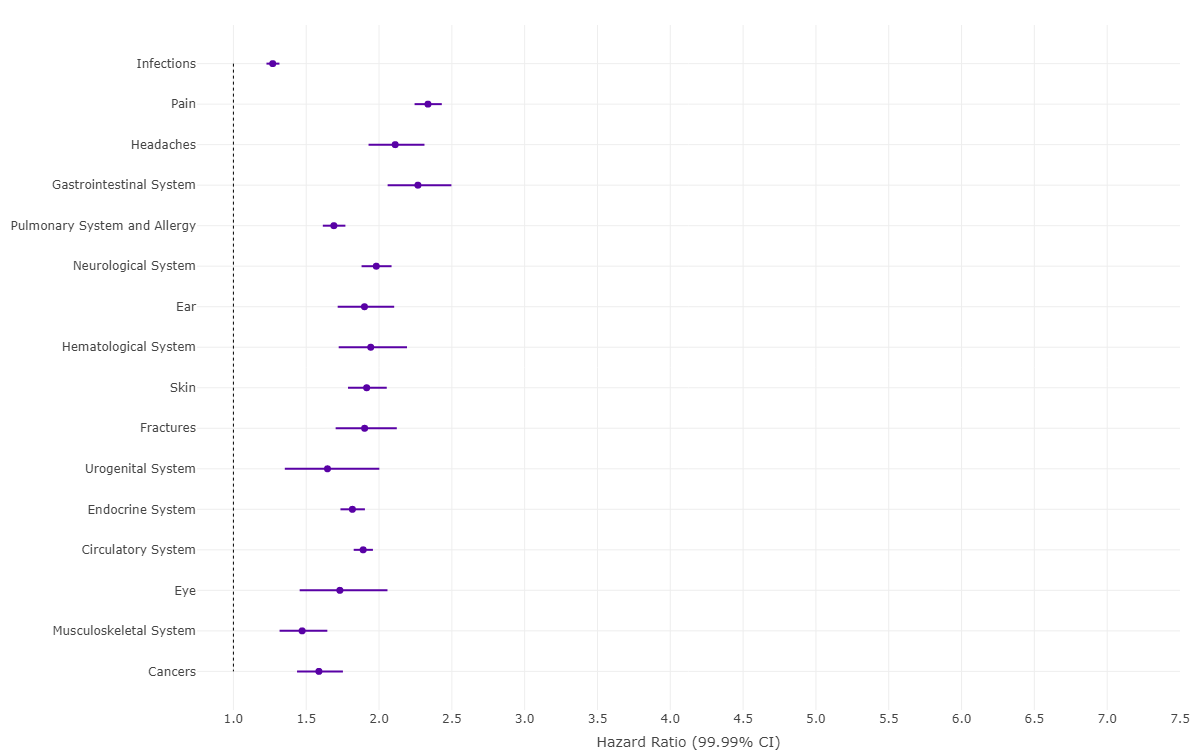 |
| **G. Psychosis** |
| 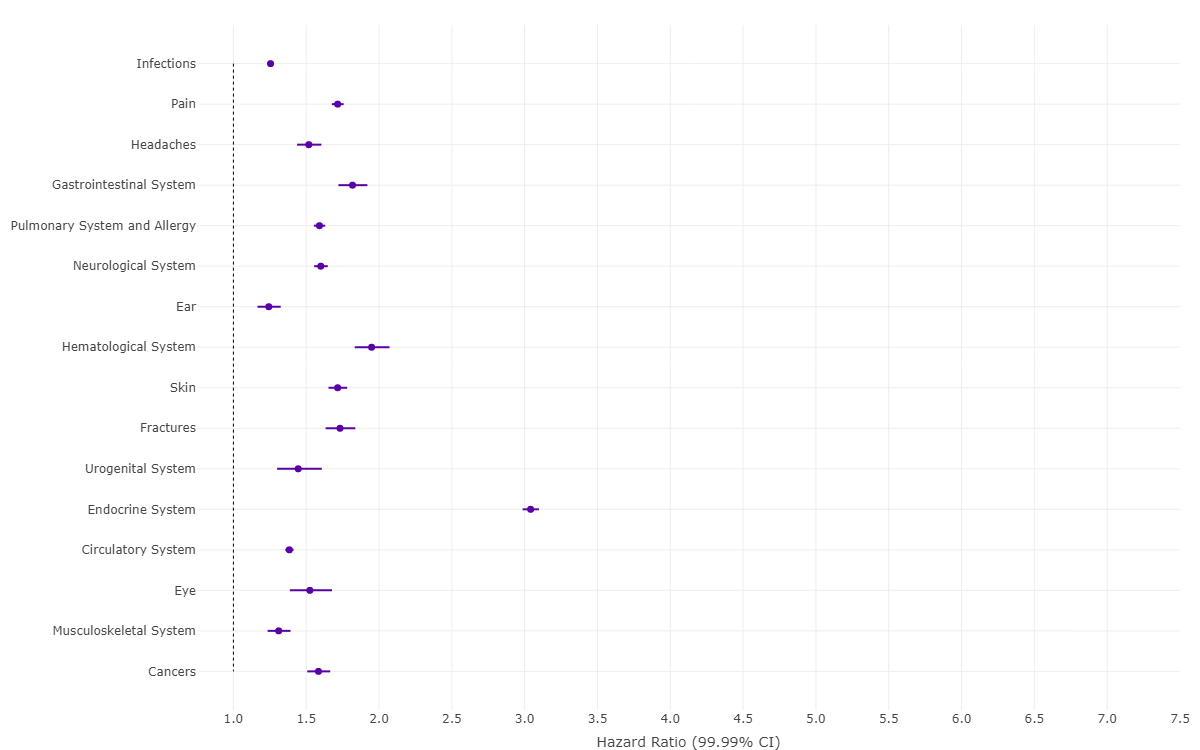 |

**Figure S15. Risk (hazard ratios) of seven specific mental disorders after diagnosis of 16 physical-health conditions across a 14-year period in primary-care patients.** Inverse probability weights were used to balance on four demographic variables: age at baseline, sex assigned at birth, educational attainment, and county of residence. Within each month from January 2006 through December 2019, encounters with primary-care providers were assessed for mental disorders and physical-health conditions. Extended Cox proportional hazards models were used, treating dependent time-to-event variables and independent exposures as recurrent and time-varying, respectively.

| **A. Sleep Disturbance** |
| --- |
| 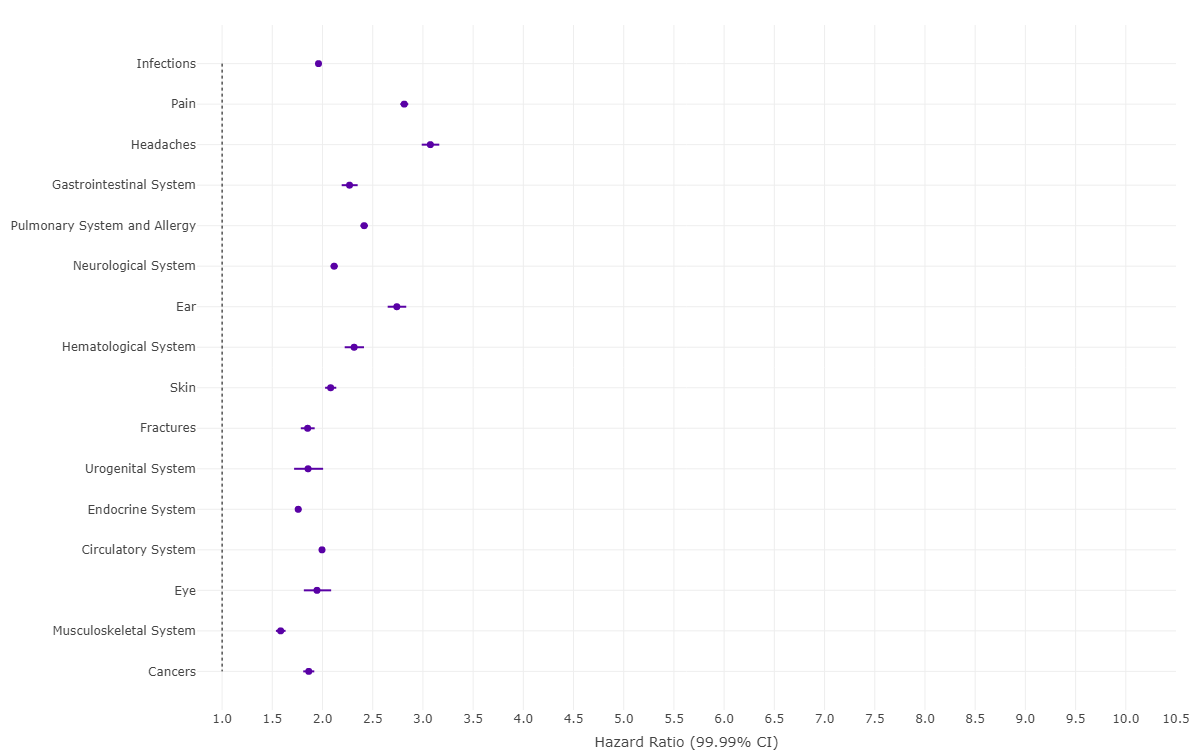 |

| **B. Anxiety** |
| --- |
| 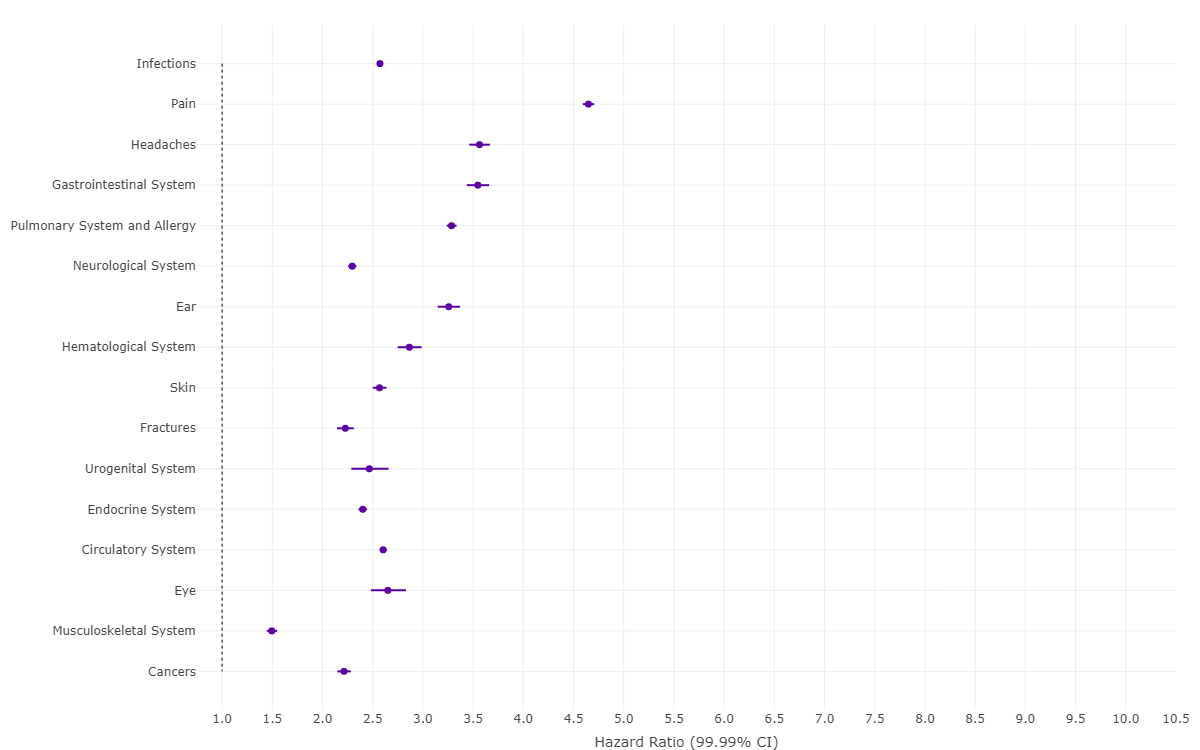 |
| **C. Depression** |
| 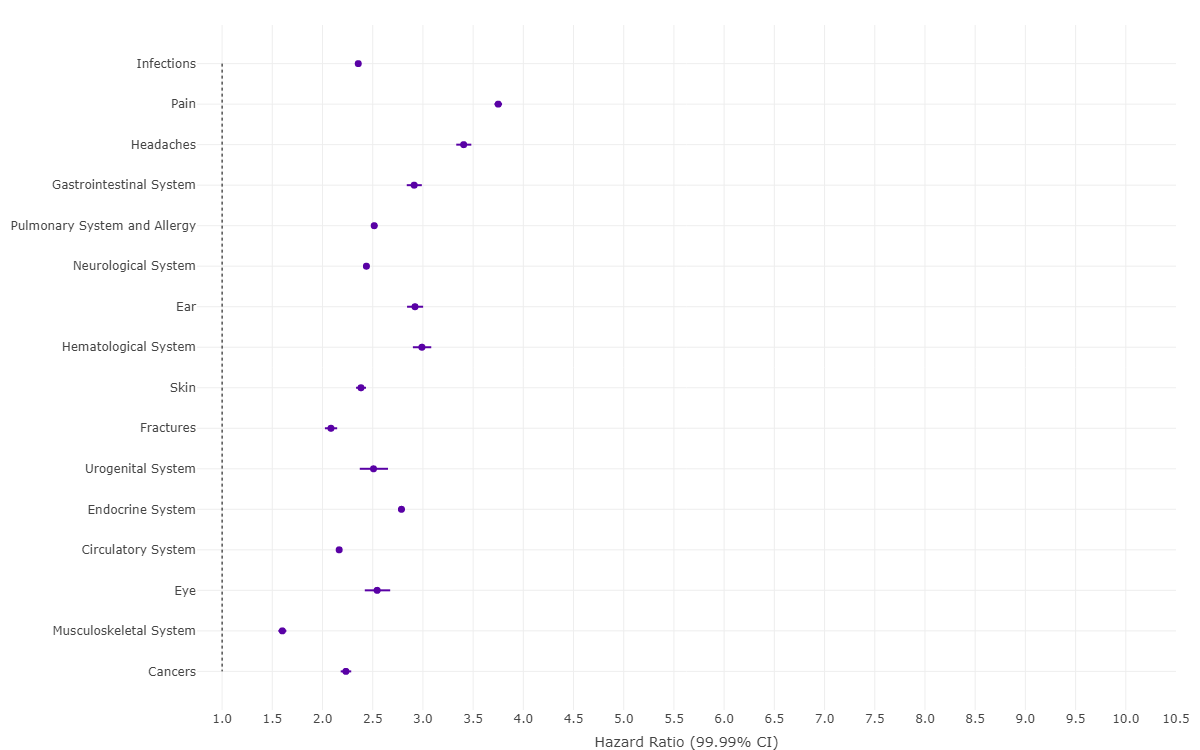 |

| **D. Acute Stress Reaction** |
| --- |
| 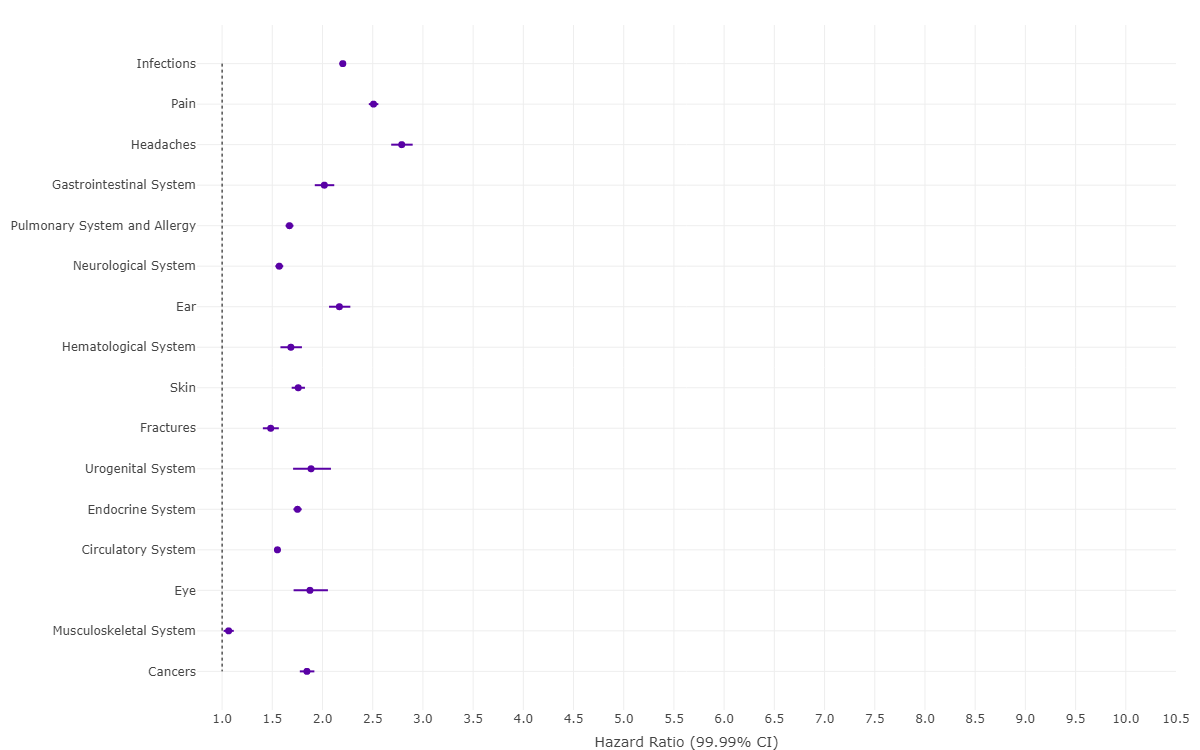 |
| **E. Substance Abuse** |
| 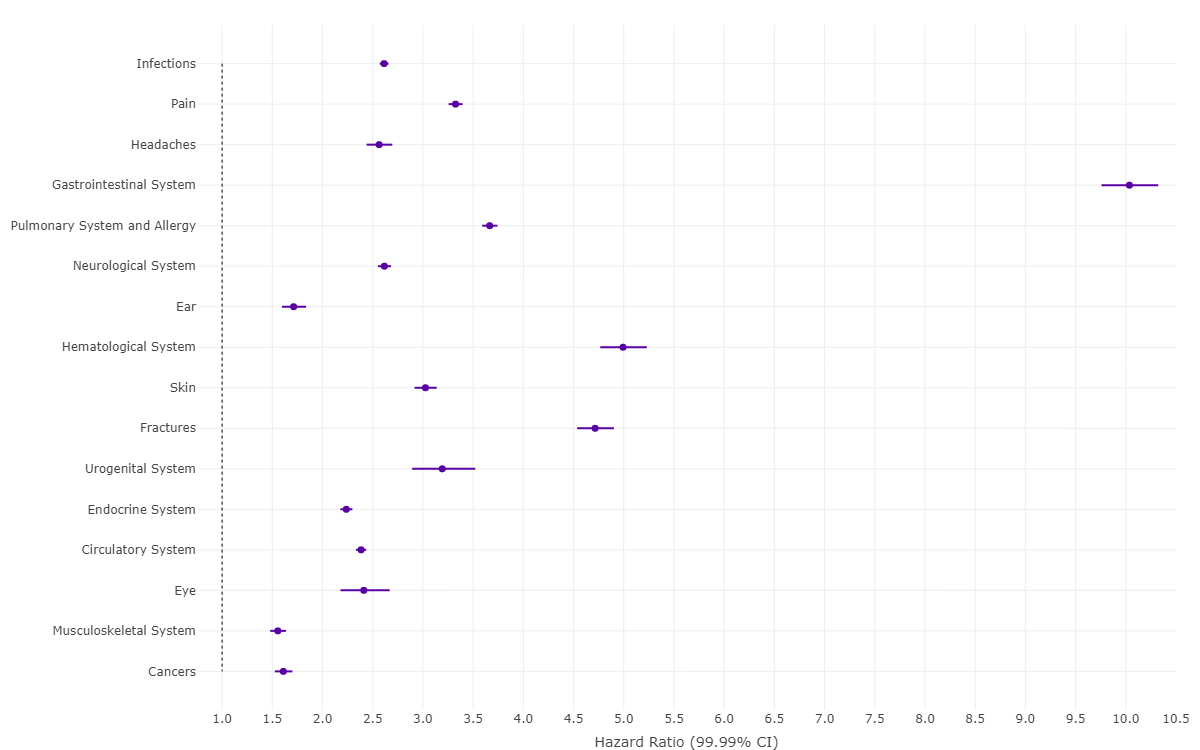 |

| **F. Phobia / Compulsive Disorder** |
| --- |
| 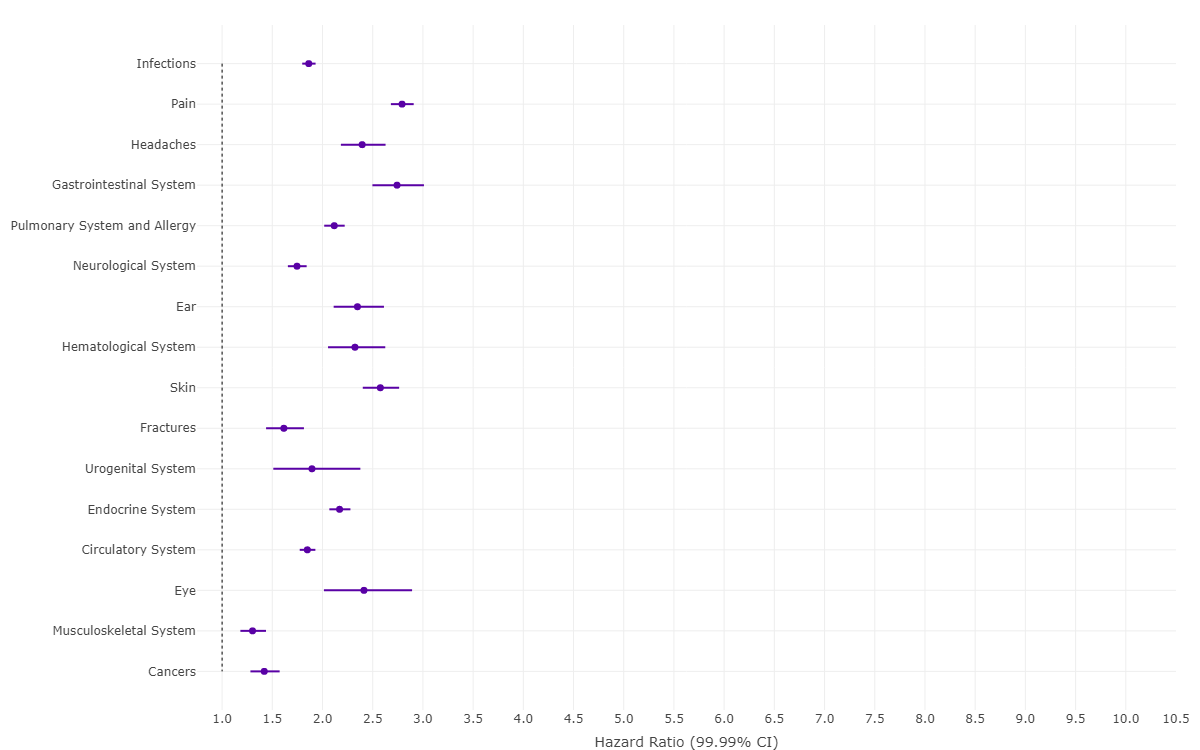 |
| **G. Psychosis** |
| 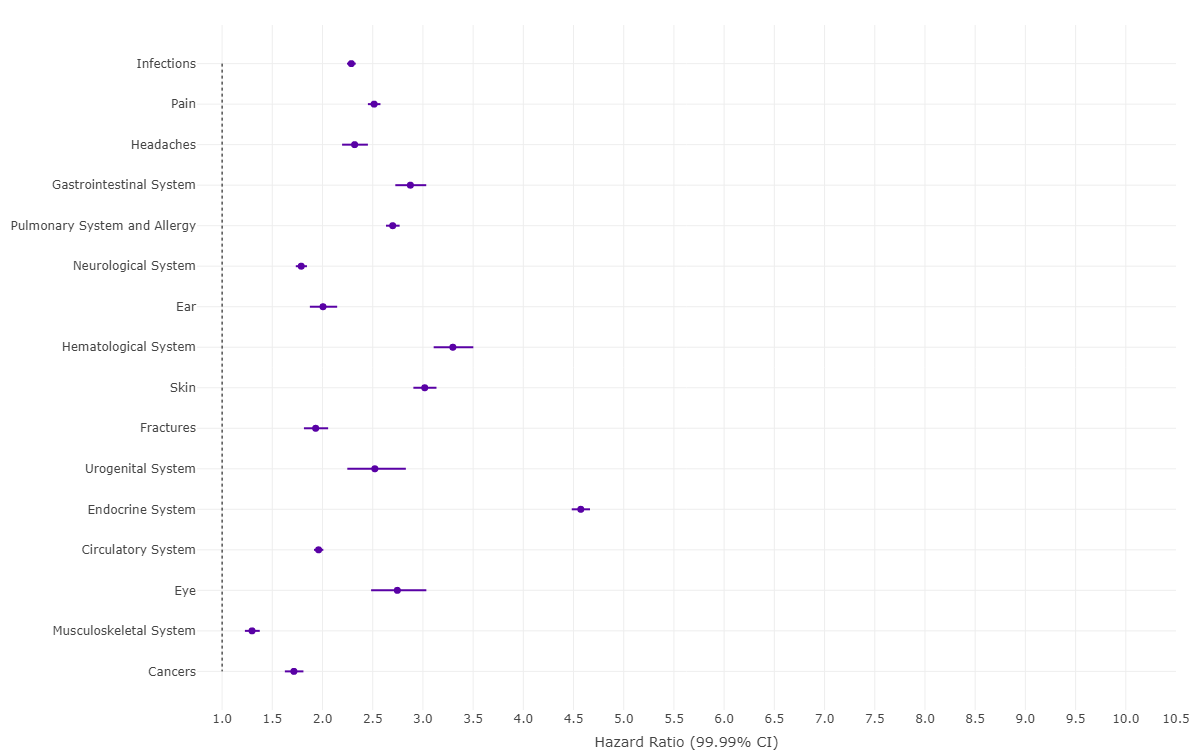 |

# **References**

1. Momen NC, Plana-Ripoll O, Agerbo E, Benros ME, Børglum AD, Christensen MK, Dalsgaard S, Degenhardt L, de Jonge P, Debost JPG, Fenger-Grøn M, Gunn JM, Iburg KM, Kessing LV, Kessler RC, Laursen TM, Lim CCW, Mors O, Mortensen PB, Musliner KL, Nordentoft M, Pedersen CB, Petersen LV, Ribe AR, Roest AM, Saha S, Schork AJ, Scott KM, Sievert C, Sørensen HJ, Stedman TJ, Vestergaard M, Vilhjalmsson B, Werge T, Weye N, Whiteford HA, Prior A, McGrath JJ. Association between Mental Disorders and Subsequent Medical Conditions. N Engl J Med. 2020;382:1721-1731.
2. The Directorate of e-health. ICPC-2e – English version: The Directorate of e-health. <https://www.ehelse.no/kodeverk-terminologi/icpc-2e--english-version>
3. Launders N, Kirsh L, Osborn DPJ, Hayes JF. The temporal relationship between severe mental illness diagnosis and chronic physical comorbidity: a UK primary care cohort study of disease burden over 10 years. Lancet Psychiatry. 2022;9:725-735.
